# DNA Methylation Diversification at the Integrated Organellar DNA-Like Sequence

**DOI:** 10.3390/genes9120602

**Published:** 2018-12-03

**Authors:** Takanori Yoshida, Yoshiaki Tarutani, Tetsuji Kakutani, Akira Kawabe

**Affiliations:** 1 Faculty of Life Science, Kyoto Sangyo University, Motoyama, Kamigamo, Kita-Ku, Kyoto 603-8555, Japan; akiraka@cc.kyoto-su.ac.jp; 2 Department of Integrated Genetics, National Institute of Genetics, Mishima, Shizuoka 411-8540, Japan; ytarutan@nig.ac.jp (Y.T.); tkakutan@nig.ac.jp (T.K.); 3 Department of Genetics, School of Life Science, The Graduate University for Advanced Studies (SOKENDAI), Mishima, Shizuoka 411-8540, Japan; 4 Faculty of Science, The University of Tokyo, Bunkyo-ku, Tokyo 113-0033, Japan

**Keywords:** DNA methylation, amplicon sequencing, NUMTs, *Arabidopsis thaliana*, epigenetic diversity

## Abstract

Plants have a lot of diversity in epigenetic modifications such as DNA methylation in their natural populations or cultivars. Although many studies observing the epigenetic diversity within and among species have been reported, the mechanisms how these variations are generated are still not clear. In addition to the de novo spontaneous epi-mutation, the intra- and inter-specific crossing can also cause a change of epigenetic modifications in their progenies. Here we report an example of diversification of DNA methylation by crossing and succeeding selfing. We traced the inheritance pattern of epigenetic modification during the crossing experiment between two natural strains Columbia (Col), and Landsberg *electa* (L*er*) in model plant *Arabidopsis thaliana* to observe the inheritance of DNA methylation in two organellar DNA-like sequence regions in the nuclear genome. Because organellar DNA integration to the nuclear genome is common in flowering plants and these sequences are occasionally methylated, such DNA could be the novel source of plant genome evolution. The amplicon sequencing, using bisulfite-converted DNA and a next-generation auto-sequencer, was able to efficiently track the heredity of DNA methylation in F_1_ and F_2_ populations. One region showed hypomethylation in the F_1_ population and succeeding elevation of DNA methylation with large variance in the F_2_ population. The methylation level of Col and L*er* alleles in F_2_ heterozygotes showed a significant positive correlation, implying the trans-chromosomal effect on DNA methylation. The results may suggest the possible mechanism causing the natural epigenetic diversity within plant populations.

## 1. Introduction

Recently, in addition to the genetic variations, a large amount of epigenetic diversity has been reported for plants [1,2,3,4]. Because it is easy to analyze the cytosine DNA methylation, many studies are focused on analyzing the status of DNA methylation. In the model plant *Arabidopsis thaliana*, DNA methylation is de novo established and/or maintained by several methyltransferases: DNA Methyltransferase 1 (MET1), Chromomethylase 3 & 2 (CMT3, CMT2), and Domains Rearranged Methyltransferase 2 (DRM2) [5,6]. DRM2 is involved in the RNA–directed DNA methylation (RdDM) with other factors such as Argonaute 4 (AGO4) and Dicer-like 2/3/4 (DCL2/3/4) [7]. In addition, there is a positive feedback of histone H3K9 methylation by SU(VAR)3-9 HOMOLOG 4/KRYPTONITE (SUVH4/KYP) and non–CpG (i.e., CpHpG and CpHpH, where H is A, T, or C) methylation by CMT3 and CMT2 [8]. The DNA methylation shows considerable within-species variations [1,4] and large divergences between different taxa [3], or even between close relatives [2]. For the analysis of DNA methylation, there are several methods that differ in their resolutions. While the approach of whole-genome methylome by next-generation sequencing (NGS) has the highest comprehensiveness and resolution for entire genomes (e.g., [9,10]), techniques such as methylation-sensitive amplified polymorphism are used as a cheaper way to analyze the whole-genome epigenetic status for multiple samples [11]. The Sanger sequencing of clones from bisulfite-converted DNA is also used for analyzing the DNA methylation status of a particular region in the genome.

Although there is a growing number of reports concerning diversity within plant genomes, the mechanism that causes epigenetic diversity within/among species is still not clear. The DNA methylation and histone modification patterns are associated with the genome architecture [12,13]. Epigenetic states induced by environmental stress can be reset and be prevented from transgenerational transmission [14]. A new spontaneous epi-mutation is brought about in the nuclear genome with relatively high forward and backward epi-mutation rates [15,16]. In addition to spontaneous epi-mutation on a single cytosine site, DNA methylation can be altered at the progeny of crossing between different strains of a species [17,18,19] or at the interspecific hybrid [20]. The ‘genome shock’ by these crossing and succeeding epigenetic and/or transcriptomic change in the progeny might be the cause of intriguing biological phenomena such as hybrid vigor, paramutations, and de novo epi-alleles. The examples of quantitative evaluation of epigenetic diversification brought about in the progenies’ population are, however, still poor, partly because of the methodology used to evaluate the level of variation.

For evaluation of epigenetic change by crossing, endogenous and/or foreign DNAs in plant nuclear genomes such as transposable elements (TEs), retrovirus-derived DNAs, and organellar DNA-like sequences that are recently derived from organellar genomes can be a good target for analysis, because they are prone to be methylated and also bring new materials for genome evolution [21,22,23,24]. While it is often difficult to estimate the methylation level of particular TE regions because of their high copy number, organellar DNA-like elements, so-called nuclear plastid DNA (NUPT), and nuclear mitochondrial DNA (NUMT) are lower copy number in the genome and can also be methylated, possibly as a result of genome defense response [25,26,27]. In this study, we attempted to trace the epigenetic modification change at organellar DNA-like sequences by crossing two natural strains of *A. thaliana* (Columbia and Landsberg *electa*). Using a next-generation sequencer, amplicon sequencing (amplicon-seq) with bisulfite-converted genomic DNA (gDNA) was conducted for F_1_ and F_2_ populations to analyze the heritability of DNA methylations on organellar DNA-like sequences. We observed a contrasting pattern of transgenerational DNA methylation change: in one organellar DNA-like sequence, the level of DNA methylation was decreased in individuals of the F_1_ population, then diversified in the F_2_ population. The results may suggest a trans-chromosomal interaction that could diversify epigenetic modifications in succeeding generations.

## 2. Materials and Methods

### 2.1. Experimental Procedures

**Figure 1:**
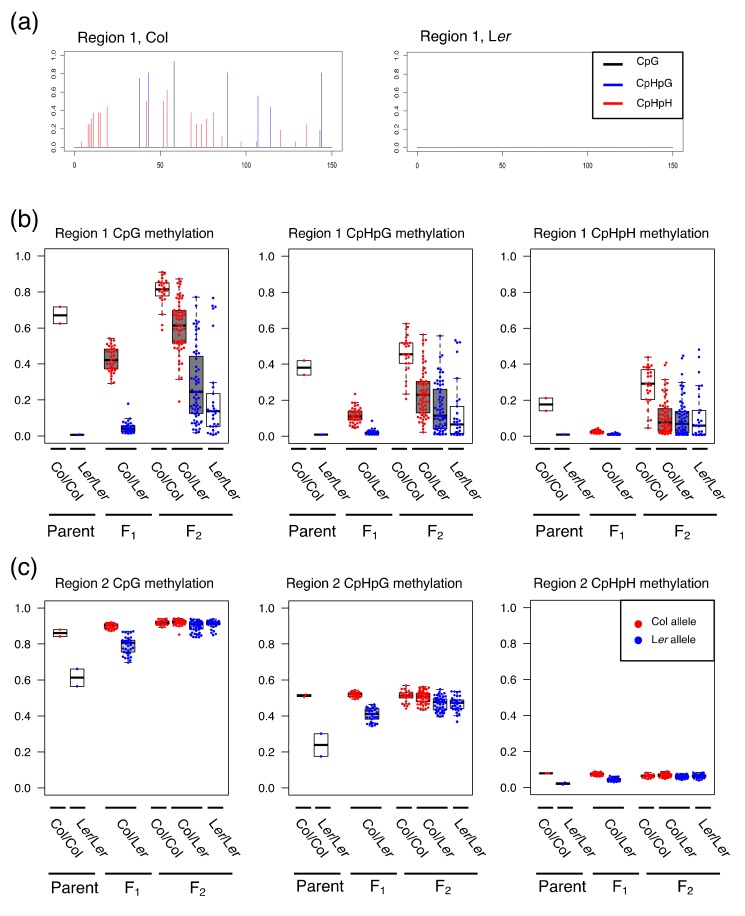
Level of DNA methylations in parents, F_1_, and F_2_. (**a**) Bar plots of DNA methylation level of Col and L*er* alleles in region 1; (**b**,**c**) The DNA methylation level of each individual for CpG, CpHpG, and CpHpH is plotted as boxplots and dots for region 1 (**b**) and region 2 (**c**). In each box, the thick line represents the median. Red and blue dots represent the methylation level of the Col and L*er* alleles, respectively.

To design the primer sets for amplification of organellar DNA-like sequences, NUPTs and NUMTs in the *A. thaliana* reference genome analyzed in previous studies [28,29] were manually inspected. In this study, nuclear genome sequences with more than 80% homology to organellar genomes were considered as candidates for the analysis of DNA methylation. We sought to find differentially methylated regions (DMR) of NUPTs/NUMTs between two strains of *A. thaliana*, Columbia (Col) and Landsberg *electa* (L*er*), to track the DNA methylation inheritance for Col and L*er* alleles separately. Using 1001 project database ([4], available in: http://neomorph.salk.edu/1001_epigenomes.html), 49 NUPTs and 36 NUMTs were inspected for methylation level difference and Single Nucleotide Polymorphisms (SNPs) sites between Col and L*er* strains. To avoid mis-priming of primers to TEs or the organellar genome during PCR, NUPTs/NUMTs located inside the TEs and those longer than 250 bp were filtered out. In total, 15 primer sets were tested for Bisulfite PCR and 2 primer sets that amplify NUMTs (region 1; Chr3: 16574335..16574467, region 2; Chr4: 6341088..6341288) were used in this study. The two regions are homologous to mitochondrial genome sequences (region 1; ChM: 15459..15600, region 2; ChM: 236742..236943). The level of DNA methylation in these regions is shown in Figure S1. Because there is no DNA methylation in L*er* allele of region 1 by the inspection of 1001 epigenome data, we checked the DNA methylation level by bisulfite Sanger sequencing to confirm the DNA methylation in the L*er* allele (Figure 1a).

Seeds of Col and L*er* were sown in Murashige and Skoog medium (MS) and grown in a plant growth chamber with the conditions of 23 °C and 16 h daylight. Reciprocal crosses were conducted and F_1_ seeds were collected from both crosses. F_1_ seeds were sown with the same conditions and fresh leaves of 18 F_1 (Col × L*er*)_ and 19 F_1 (L*er* × Col)_ individuals were collected. Two individuals from both F_1_ populations were selected to collect seeds by self-pollination. For each, 28 to 30 individuals were selected as F_2_ populations (F_2 (Col × L*er* ind.2 Self)_, F_2 (Col × L*er* ind.4 Self)_, F_2 (L*er* × Col ind.3 Self)_, and F_2 (L*er* × Col ind.4 Self)_). The number of collected samples is shown in Table 1. Genomic DNA was extracted from fresh leaves of F_1_ and F_2_ individuals using a DNeasy Plant Mini Kit (QIAGEN K.K., Tokyo, Japan). Extracted gDNAs were bisulfite-converted by a MethylCode Bisulfite Conversion Kit (Thermo Fisher Scientific K.K., Tokyo, Japan). Bisulfite-converged DNAs were used for PCR to construct the sequencing library for MiSeq autosequencer (Illumina K.K., Tokyo, Japan). We modified the protocol of 16S Metagenomic Sequencing Library Preparation (available from http://support.illumina.com) for amplicon-seq of two regions in the nuclear genome of *A. thaliana*. In brief, PCR primers with overhang adapters were designed for target regions (Table S1). Then, eight cycles of PCR were conducted to attach indices (Nextera XT Index Kit, Illumina) that enable us to distinguish each individual. Products with indices were normalized and pooled in the sequencing library. To improve the sequencing quality, PhiX Control (30% of sequencing library volume) was added by following the instructions from Illumina. The sequencing library was sequenced by a MiSeq autosequencer (Illumina) with 300 cycles (150 × 2). Reads were classified into individuals by attached indices.

### 2.2. Data Analysis

Quality of sequenced reads was inspected by program FastQC v. 0.11.5 (available from https://www.bioinformatics.babraham.ac.uk/). Reads with low quality were discarded by program Trimmomatic ver. 3 ([30], available in: http://www.usadellab.org/). Sequences of amplified regions (Supplementary File 1) were used for preparing reference sequence by script *bismark_genome_preparatopm* in Bismark suite v. 0.14.5 ([31], available from https://www.bioinformatics.babraham.ac.uk/). Then, treated reads were mapped to the reference sequences by program *bismark*. For F_1_ and F_2_ individuals, reads from Col and L*er* alleles were distinguished by four and two SNP sites for region 1 and 2. The level of DNA methylation in cytosine sites was extracted by script *bismark_methylation_extractor* in Bismark suite. The commands used in data analysis are described in Supplementary File 2. For analysis of DNA methylations, cytosine sites shared by both Col and L*er* alleles were selected and used for subsequent analyses of DNA methylations (region 1: two sites with CpG, five sites with CpHpG, and 23 sites with CpHpH contexts, region 2: 10 sites with CpG, six sites with CpHpG, and 33 sites with CpHpH contexts, Table S2). From the extracted methylation data, the weighted methylation level [32] was calculated for each allele of each individual and each cytosine motif by the following equation:

(1) $$M_{weighted}=\frac{\sum_{i=1}^{n}C_{i}}{\sum_{i=1}^{n}\left(C_{i}+T_{i}\right)}\;,$$

in which *C*_i_ and *T*_i_ represent methylated (*C*_i_) and unmethylated (*T*_i_) cytosine counts of the ith cytosine site with CpG/CpHpG/CpHpH context.

To estimate the epigenetic regulation genes possibly involved in DNA methylation in the regions, genome-wide methylome data from mutant lines of epigenetic regulation genes [33] were analyzed. The data of single base-pair methylation level were downloaded from Gene Expression Omnibus (GEO, available in: https://www.ncbi.nlm.nih.gov/geo/) for mutants associated with epigenetic regulations (GSE39901). The fractional methylation level [32] was calculated for each region to compare the DNA methylation level between the wild type and mutants. 

## 3. Results

### 3.1. Summary of Amplicon Sequencing

For individuals, over 40,000 reads were mapped (Table S3, minimum mapped reads; 43,686, maximum mapped reads; 507,516). The number of reads mapped to each allele was compared with each other. If the number of reads mapped to one allele was smaller than that mapped to the other allele (less than 5% of total mapped reads), the reads were regarded as mapping error and not used in the following analysis. The rate of probable mapping error was small enough (median = 0.8%). For individuals in F_1_ populations, nearly equal amounts of reads were mapped for Col and L*er* alleles, suggesting the mapping/distinguishing strategy of Col and L*er* reads by SNPs works well and mapping errors at heterozygotes can be ignored. The genotype of individuals in F_2_ (Col/Col; CC, Col/L*er*; CL, L*er*/L*er*; LL) was detected from mapped reads and the results are summarized in Table S4. F_2_ populations showed no deviation from the expected segregation ratio (CC = 0.25, CL = 0.5, LL = 0.25), except for region 2 of the F_2(Col × L*er* ind.2 Self)_ population showing a weak significance (Chi-square = 7.9, *p* = 0.02). These results indicate that the process of sequencing, mapping, and distinguishing Col/L*er* reads is appropriate and there is no distortion of segregation in regions used. For Col and/or L*er* alleles of each individual, the DNA methylation levels with different sequence motifs (CpG, CpHpG, CpHpH) were estimated (Table S5).

### 3.2. Fluctuation of DNA Methylation Level in F_1_ and F_2_ Populations in Region 1

The DNA methylation could be differently reset and re-initiated in maternal and paternal gametogenesis, resulting in a different level of DNA methylation in each Col and L*er* allele between reciprocal crosses. For each allele in an F_1_ individual, we inspected the distribution of methylation level and tested the difference of distributions between reciprocal crosses (Figures S2 and S3). There were no strong significant differences in distribution (*p* < 0.001). Thus, in the following analyses, we merged data of reciprocal crosses in F_1_ and F_2_ populations. Although there might be a difference of inheritance between male/female gametes for the analyzed regions, the effects could be relatively small compared to other factors affecting the methylation levels in these regions.

In region 1, we observed a drastic change of DNA methylation pattern from parents through F_2_ offspring. The medians of DNA methylation level of parent Col and L*er* in region 1 were 0.67/0.01 for CpG, 0.38/0.01 for CpHpG, and 0.18/0.01 for CpHpH motifs, respectively (Figure 1, Table 2). In the F_1_ population, a decrease of DNA methylation was observed in region 1 for all motifs (CpG, CPHpG, and CpHpH). In the F_1_ population, the values in the Col allele were 0.42 for CpG, 0.11 for CpHpG, and 0.02 for CpHpH, while the values in the L*er* allele remained largely unchanged (0.04 for CpG, 0.02 for CpHpG, 0.01 for CpHpH). In the F_2_ population, in contrast to the F_1_ population, the DNA methylation level of both Col and L*er* alleles was increased and largely diversified for all three motifs. The standard deviations of Col and L*er* alleles in F_2_ were larger than those in F_1_ for all motifs (Table 2). In particular, the difference was prominent in L*er* alleles, because their methylation levels were small in the F_1_ population. In contrast to the change of DNA methylation in region 1, the DNA methylation level of both Col and L*er* alleles in region 2 showed convergence to a certain level of DNA methylation for all motifs. The medians of parent Col and L*er* were 0.86/0.61 for CpG, 0.51/0.24 for CpHpG, and 0.08/0.02 for CpHpH, respectively. The methylation level of L*er* alleles was then increased in succeeding generations (Table 2). Compared to region 1, region 2 showed a relatively small difference of dispersion for DNA methylation level between the F_1_ and F_2_ populations.

### 3.3. Correlation of Region 1 Methylation Level between Alleles in F_2_ Heterogyzotes

**Figure 2:**
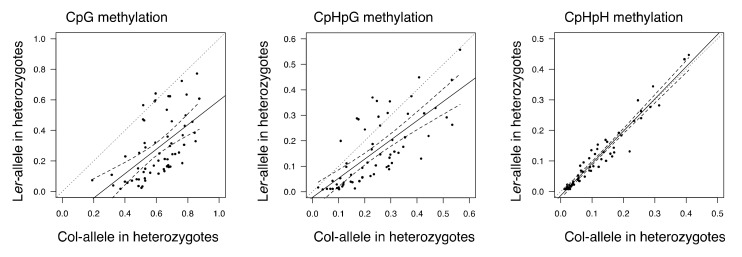
Methylation level correlation between Col and L*er* alleles in F_2_ heterozygotes. Plot of methylation level correlation between Col and L*er* alleles in F_2_ heterogyzotes. Solid lines represent best-fit linear regression lines. Dashed lines represent 95% lower and upper bounds of regression.

In region 1, the DNA methylation level of Col allele in F_1_ and F_2_ populations were higher than those of L*er* alleles (Figure 1 and Figure S2). The increase of DNA methylation in L*er* alleles in the F_2_ population could be caused by *trans*-chromosomal effect of Col allele. The DNA methylation levels in F_2_ heterozygotes showed significant correlation between Col and L*er* alleles (CpG; *ρ* = 0.57, *p* = 8.50E-7, CpHpG; *ρ* = 0.75, *p* = 2.15E-12, CpHpH; *ρ* = 0.97, *p* < 2.20E-16). The DNA methylation level with each motif showed positive regression coefficients (0.800 for CpG, 0.751 for CpHpG, and 1.054 for CpHpH, Figure 2). The result may indicate a *trans* effect, possibly from a chromosome derived from Col to that from L*er*, that influences the methylation level of region 1 in the F_2_ population. The trans-chromosomal effect can be caused by small interference RNA (siRNA) and the RNA-directed DNA methylation (RdDM) machinery. There are several reads of small RNA in both region 1 and region 2 (Figure S4). Whether siRNA will be expressed from other regions in the Col nuclear genome, the sequence having similarity to region 1 was confirmed by the program *blastn* implemented in NCBI BLAST ver.2.6.0 (https://blast.ncbi.nlm.nih.gov/) with default settings (Table S6). There was no significant hit more than 50 bp long, suggesting that small RNAs expressed only from this region can mainly affect the DNA methylation by trans-chromosome interaction.

### 3.4. DNA Methylation in Mutant Lines

In region 1, the *ago4* and *dcl2/3/4* mutants showed a decreased level of non-CpG methylation compared to that of wild type (WT) (Table 3, Figure S5). It may indicate that the DNA methylation in this region is in part dependent on the RdDM machinery. In addition, *ddm1*, *suvh4/kyp*, and *met1* showed a more remarkable decrease of DNA methylation for both CpG and non–CpG contexts (Table 3, Figure S5). It suggests that maintenance methylation and chromatin remodeling are associated with the DNA methylation in this region. In region 2, there was no remarkable decrease of DNA methylation in mutant lines of *ago4* and *dcl2/3/4*, while *ddm1*, *suvh4/kyp*, and *met1* showed a decrease in DNA methylation (Table 3, Figure S6). This suggests that RdDM machinery is not involved in the DNA methylation of region 2.

## 4. Discussion

In previous studies, as well as genetic variation, a high epigenetic diversity is reported for natural population of *A. thaliana* [1,4]. Other plants also have epigenetic diversity in their populations (e.g., [34,35,36]). Some studies indicate the act of natural selection on the epigenetic variations for their relation to heat stress response [37,38] or for different environmental conditions [39]. If the epigenetic modification on DNA or histone is the target of selection, the diversity of such epigenetic modification should be given more attention as a source of variation. While there are some reports focused on the epigenetic variation, the mechanism by which these variations are newly generated, inherited, or maintained, and how they are erased, is still not clear. In addition to the epi-mutation at a single site of *A. thaliana* mutation accumulation lines [15], it is shown that both intra- and interspecific crossing can cause the difference of epigenetic modifications [17,18,19,20]. The insertion of TEs near genes can create TE- and repeat-associated epimutable alleles. During the cross between different strains, such epimutable alleles can be DNA methylated with a transchromosomal effect of other TEs on different chromosomes [16]. In this study, we observed two contrasting trajectories of DNA methylation inheritance and diversification of methylation of organellar DNA-like sequences in the F_1_ and F_2_ populations. While region 2 showed an almost even level of DNA methylation in the F_2_ population for all cytosine motifs, the DNA methylation level in region 1 varied considerably among individuals. In both regions, there might be a trans-chromosomal effect on the methylation level, possibly from the Col-allele chromosome to the L*er*-allele’s. In region 1, methylation levels of Col and L*er* alleles in F_2_ heterozygotes showed a positive correlation, suggesting that the elevation of methylation levels of L*er* alleles could somewhat depend on the methylation levels of Col alleles. There could be a mechanism to balance the level of DNA methylation between alleles within a specific locus. In region 2, the methylation levels of L*er* alleles were elevated in both F_1_ and F_2_, resulting in the same level of DNA methylation as in Col alleles.

A well-known trans-chromosomal epigenetic effect is paramutation, found in the maize *red colour 1* (*r1*) locus involved in anthocyanin biosynthesis [40]. Several loci are reported as paramutable in maize and show 100% penetrance and heritability (e.g., *red1*, *booster1*, *purple plant1*). The paramutations and/or paramutation-like phenomena are also reported in other plants such as *A. thaliana*, tobacco, tomato, etc. [41]. The multiple epigenetic mechanism, including not only the RdDM pathway but also genes indirectly involved in DNA methylation such as *DDM1*, could be associated with the paramutation and paramutation-like phenomena [42]. Because there are several reads of small RNA that match the regions analyzed in this study, the RdDM mechanism might be involved in the methylation pattern of the two regions. Because a decrease in non–CpG DNA methylation in *ago4* and *dcl2/3/4* mutants was observed for region 1 (Table 3, Figure S5), the RdDM machinery may be involved in the change of DNA methylation observed at region 1 in F_1_ and F_2_. The involvement of RdDM machinery in region 1 could cause different trajectories between region 1 and 2. In addition, regions 1 and 2 both showed a substantial decrease in DNA methylation in the *ddm1* and *suvh4/kyp* mutants (Table 3, Figure S5). This suggests that the genes related to the maintenance DNA methylation are involved in the DNA methylation in both regions. As suggested in a previous study [42], they could also be associated with the trans-chromosomal DNA methylation observed in regions 1 and 2. In contrast to region 1, region 2 showed little effect of RdDM machinery on the DNA methylation in the region. Although the details are unknown, this might suggest that the elevation of DNA methylation in region 2 is caused by another mechanism such as chromatin remodeling.

This study demonstrates that crossing individuals with different methylation levels can generate diversity of DNA methylation in the progenies, though the pattern is complex. In region 1, DNA methylation levels were decreased in F_1_ populations, especially for non-CpG motif cytosines. Then, however, the medians of methylation levels were increased with large variance in F_2_ populations. The trans-chromosomal methylation and demethylation in hybrids of *A. thaliana* were also shown in a previous study [17,18]. The difference between F_1_ and F_2_ might indicate the difference in trans-chromosomal effect associated with the expression of small RNAs in F_1_ and F_2_. The result implies the complexity of determining the DNA methylation level of the region after crossing different strains. The process of DNA methylation change was not completed in one generation; rather, it may take several or more generations to reach a new plateau of methylation. In the previous study of *ddm1* mutant, ectopic accumulation of DNA methylation at many loci with the *ddm1* mutant was observed after nine repeated self-pollinations of genome-wide hypomethylated *ddm1* [43]. Instead of being a single state of plateau, the methylation level in this region could be diversified among progenies and retain multiple status of DNA methylation. Both Col and L*er* alleles of homozygotes in F_2_ also showed diversified DNA methylation, indicating that the hybridization and succeeding selfing could cause allelic methylation differences. If the diversification observed in this study is rather common in plants, it could indicate the contribution of integrated organellar DNA-like sequences or other foreign DNA to generate the epigenetic diversity of the plant genome. 

TEs or repetitive sequences near genes can affect the gene expression. In *A. thaliana* and related species, DNA methylations on short interspersed nuclear elements (SINE)-related sequences at 5’ region control the expression of *FWA* [35,44,45]. In *Arabidopsis*-related species, there are intraspecific variations of DNA methylation at the 5’ regions of transcription start site that are associated with the expression of *FWA*, though there is no genetic variation in the region [35,45]. We might hypothesize that these DNA methylation variations within populations are incorporated or maintained by outcrossing between individuals, as previously proposed for population-level diversification via epigenetic phenomena [46]. For outcrossing species such as *Arabidopsis lyrata*, one of the close relatives of *A. thaliana*, the diversifying effect by outcrossing is relatively common, while a selfing species like *A. thaliana* might diversify the pattern of DNA methylation by occasional outcrossing. By using other natural strains containing intraspecific variations of integrated organellar DNA-like sequences near genes and tracking DNA methylation inheritance, the effect of crossing on methylation and expression could be clarified.

## Figures and Tables

**Table 1:** The number of samples used for amplicon-Seq.

Sample Type	Sample Description	N
Parent 1	Col	2
Parent 2	L*er*	2
F_1_	Col × L*er*	18
F_1_	L*er* × Col	19
F_2_	Col × L*er* ind.2 Self	30
F_2_	Col × L*er* ind.4 Self	28
F_2_	L*er* × Col ind.3 Self	30
F_2_	L*er* × Col ind.4 Self	30

Col: Columbia, L*er*: Landsberg *electa*.

**Table 2:** Diversification of DNA methylation level.

Region	Group		Parent		F_1_		F_2_			
	Genotype		Col/Col	L*er*/L*er*	Col/L*er*		Col/Col	Col/L*er*		L*er*/L*er*
	Allele		Col	L*er*	Col	L*er*	Col	Col	L*er*	L*er*
**Region 1**	**Motif:**									
	CpG	median	0.67	0.01	0.42	0.04	0.81	0.61	0.24	0.14
		mean	0.67	0.01	0.42	0.05	0.80	0.61	0.28	0.21
		s.d. ^1^	-	-	0.070	0.031	0.080	0.150	0.200	0.240
	CpHpG	median	0.38	0.01	0.11	0.02	0.45	0.23	0.11	0.066
		mean	0.38	0.01	0.12	0.02	0.45	0.24	0.16	0.13
		s.d.	-	-	0.043	0.014	0.110	0.130	0.130	0.170
	CpHpH	median	0.18	0.01	0.02	0.01	0.29	0.08	0.06	0.06
		mean	0.18	0.01	0.02	0.01	0.28	0.11	0.10	0.11
		s.d.	-	-	0.007	0.003	0.110	0.097	0.100	0.140
**Region 2**	**Motif:**									
	CpG	median	0.86	0.61	0.90	0.81	0.92	0.92	0.91	0.92
		mean	0.86	0.61	0.90	0.79	0.92	0.92	0.90	0.91
		s.d.	-	-	0.014	0.048	0.013	0.014	0.029	0.022
	CpHpG	median	0.51	0.24	0.52	0.41	0.51	0.51	0.48	0.47
		mean	0.51	0.24	0.52	0.41	0.51	0.50	0.47	0.47
		s.d.	-	-	0.011	0.036	0.032	0.034	0.038	0.040
	CpHpH	median	0.08	0.02	0.07	0.05	0.07	0.07	0.06	0.06
		mean	0.08	0.02	0.07	0.04	0.06	0.07	0.06	0.06
		s.d.	-	-	0.007	0.009	0.008	0.009	0.008	0.011

^1^ Standard deviation.

**Table 3:** Level of DNA methylation at mutants associated with epigenetic regulation.

Region	Strain	DNA Methylation Rate		
		CpG	CpHpG	CpHpH
**Region 1**	WT	0.885	0.405	0.147
	*ago4*	0.848	0.332	0.032
	*dcl2/3/4*	0.893	0.292	0.024
	*ddm1*	0.330	0.176	0.044
	*suvh4/kyp*	0.677	0.131	0.045
	*met1*	0.041	0.226	0.014
**Region 2**	WT	0.888	0.526	0.067
	*ago4*	0.902	0.488	0.042
	*dcl2/3/4*	0.845	0.439	0.053
	*ddm1*	0.262	0.287	0.007
	*suvh4/kyp*	0.883	0.068	0.029
	*met1*	0.002	0.210	0.006

**WT: Wild type.**

## Supplementary Materials

The following are available online at http://www.mdpi.com/2073-4425/9/12/602/s1, Figure S1: DNA methylation at parental strains, Figure S2: Level of region 1 DNA methylation in parents, F1, and F2, Figure S3: Level of region 2 DNA methylation in parents, F1, and F2, Figure S4: Small RNA expression at parental strains, Figure S5: DNA methylation of region 1 in mutant lines, Figure S6: DNA methylation of region 2 in mutant lines, Table S1: Primers used in the amplicon-Seq, Table S2: Position and motif (CpG, CpHpG, and CpHpH) in analyzed sequence, Table S3: Mapped data of bisulfite amplicon-Seq, Table S4: Genotype of F2 populations, Table S5: Level of DNA methylation, Table S6: BLASTN search of region 1 & 2 in nuclear genome, Supplementary File 1: Sequences of analyzed regions, Supplementary File 2: Commands of data analysis.
