# Supplementary material for: DNA Methylation Diversification at the Integrated Organellar DNA-Like Sequence
Source: Genes (Basel). 2018 Dec 3;9(12):602. doi: 10.3390/genes9120602 (PMC6316516; doi:10.3390/genes9120602)
Supplement: Supplementary file 1 [file genes-09-00602-s001.zip › Supplementary_Figures_revise1.pdf]

(a) Region 1 (Chr3: 16,574,327..16,574,531)

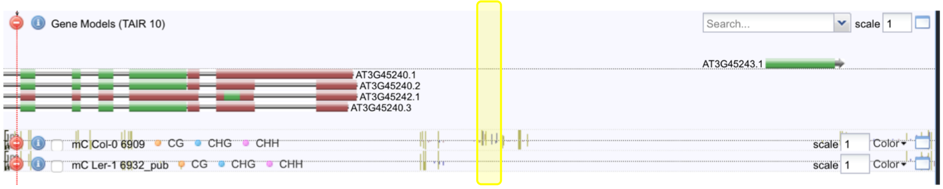

(b) Region 2 (Chr4: 6,341,136..6,341,395)

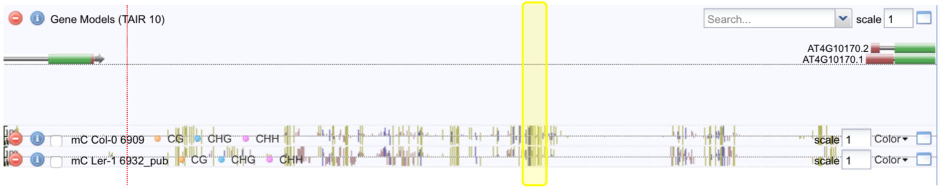

Figure S1 DNA methylation at parental strains. For parental strains Col and L*er*, the genome browser snapshots of level of DNA methylation were shown for region 1 (a) and 2 (b). Each region was highlighted by yellow.

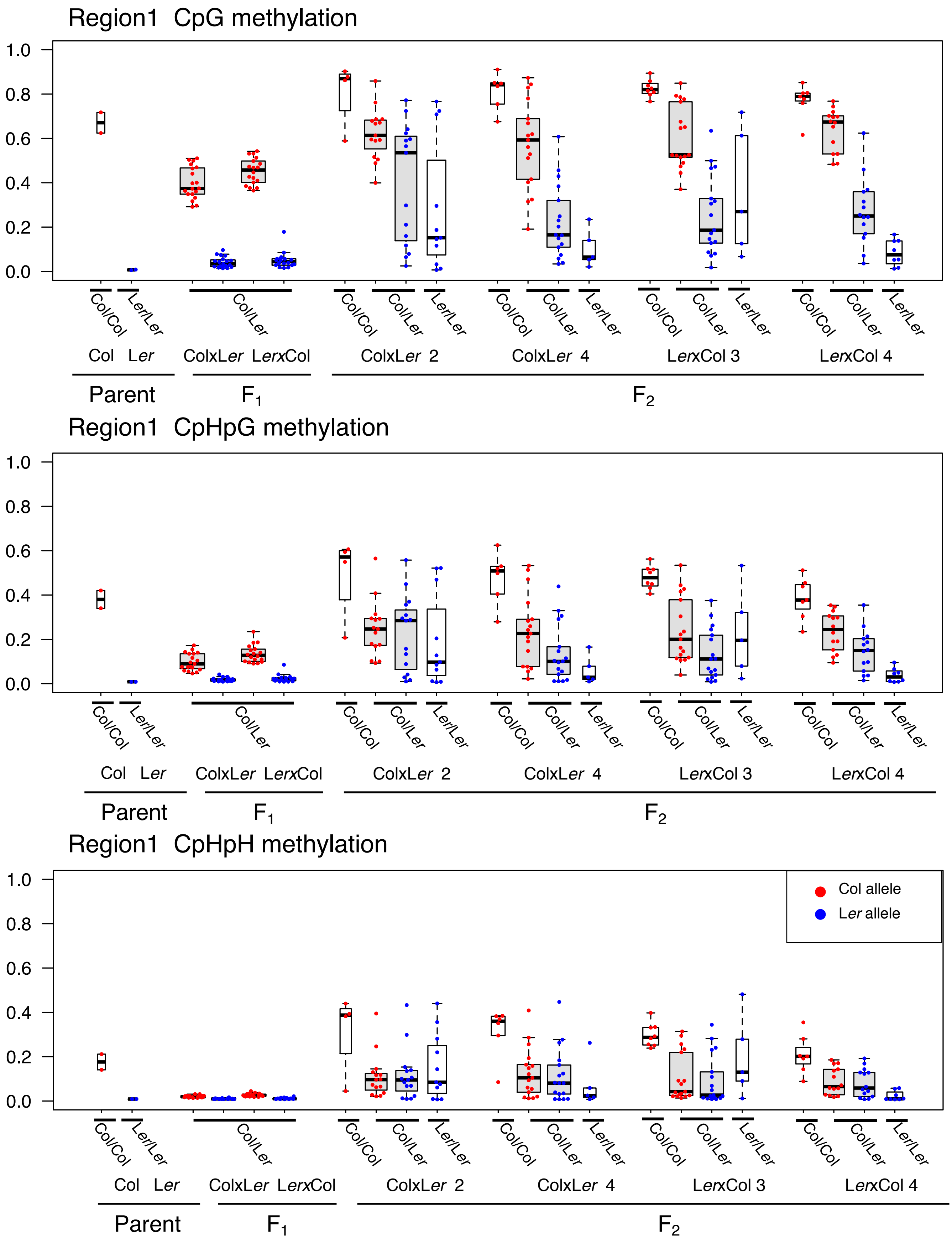

Figure S2 Level of region 1 DNA methylation in parents, F_1, and F_2. The DNA methylation level of each individual for CpG, CpHpG, and CpHpH is plotted as boxplots and dots. In each box, thick line represents median. Dark boxes shows the data of heterozygous individuals. Red and blue dots represent the methylation level of Col and *Ler* allele, respectively.

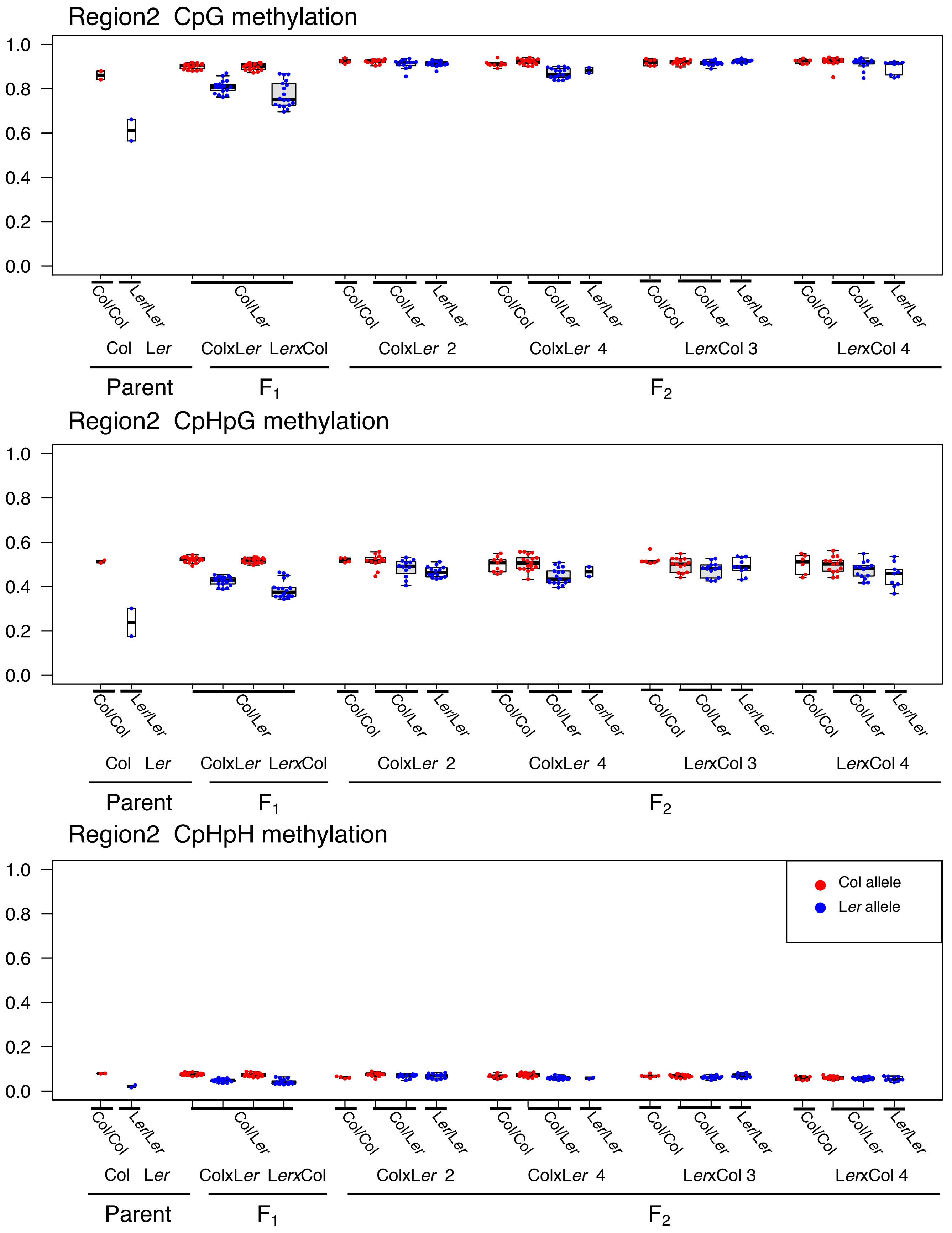

Figure S3 Level of region 2 DNA methylation in parents, F_1, and F_2. The DNA methylation level of each individual for CpG, CpHpG, and CpHpH is plotted as boxplots and dots. In each box, thick line represents median. Dark boxes shows the data of heterozygous individuals. Red and blue dots represent the methylation level of Col and L*er* allele, respectively.

(a) Region 1 (Chr3: 16,574,327..16,574,531)

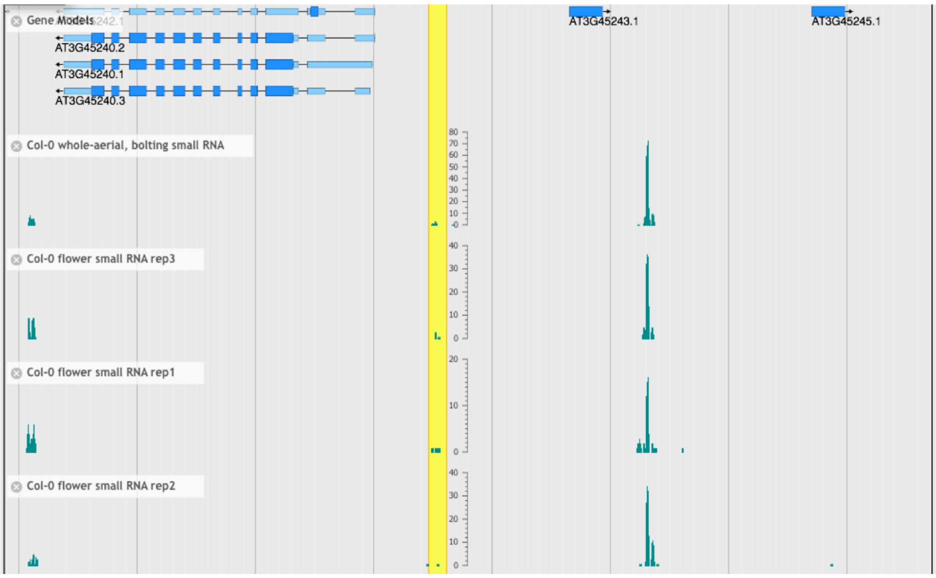

(b) Region 2 (Chr4: 6,341,136..6,341,395)

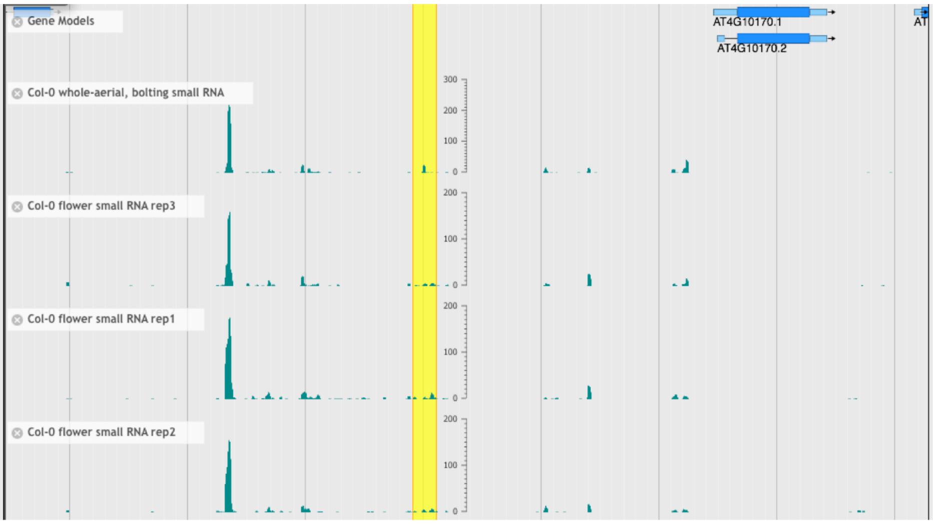

Figure S4 Small RNA expression at parental strains. For parental strains Col and L*er*, the genome browser snapshots of small RNA expression were shown for region 1 (a) and 2 (b). Each region was highlighted by yellow.

Region 1 (Chr3: 16,574,327..16,574,531)

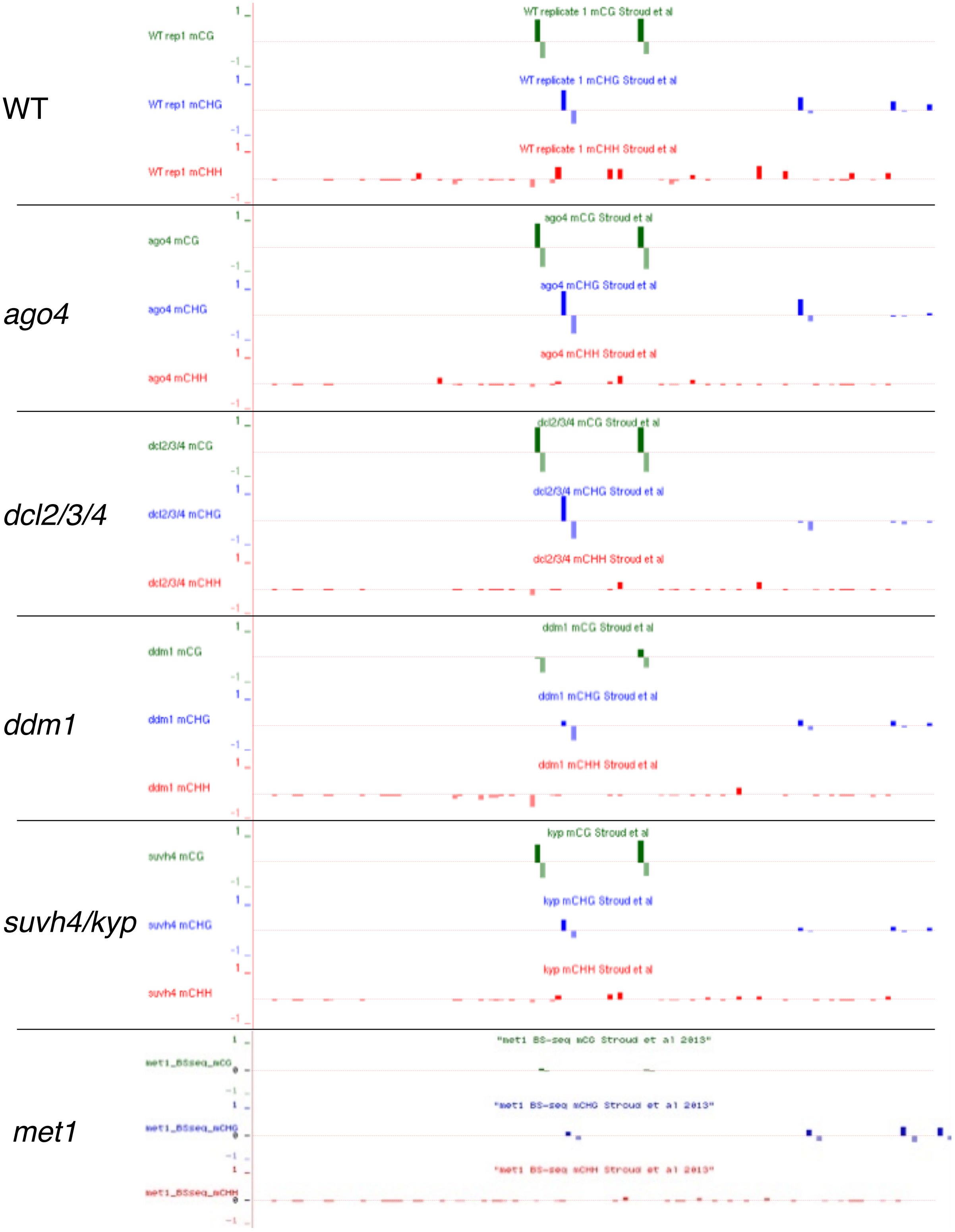

Figure S5 DNA methylation of region 1 in mutant lines. Bar plots of DNA methylation level per site were shown for mutants of genes associated with epigenetic regulation. Green, blue, and red bars represent the methylation level of CpG, CpHpG, and CpHpH sites, respectively.

Region 2 (Chr4: 6,341,136..6,341,395)

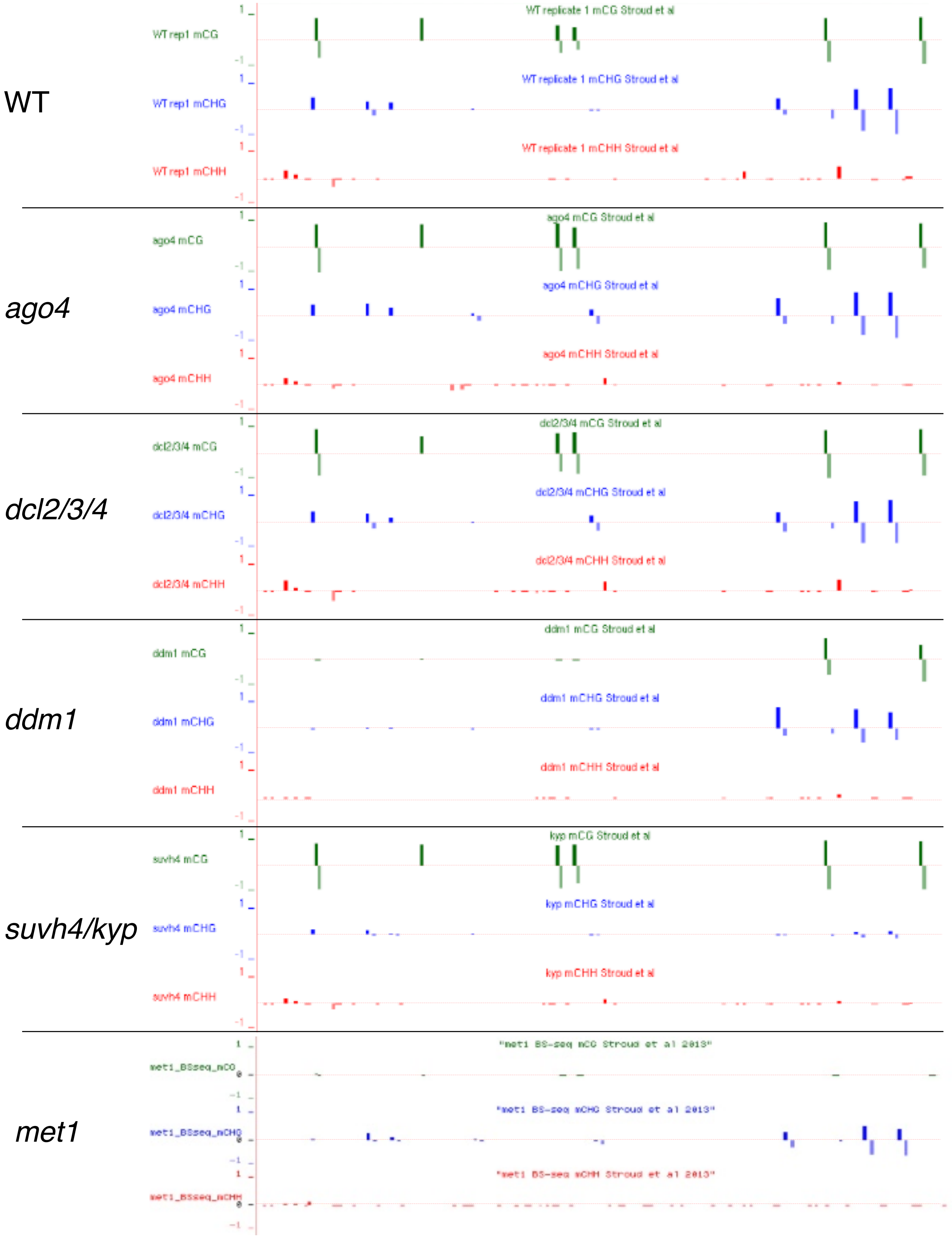

Figure S6 DNA methylation of region 2 in mutant lines. Bar plots of DNA methylation level per site were shown for mutants of genes associated with epigenetic regulation. Green, blue, and red bars represent the methylation level of CpG, CpHpG, and CpHpH sites, respectively.
