# Supplementary material for: DNA Methylation Diversification at the Integrated Organellar DNA-Like Sequence
Source: Genes (Basel). 2018 Dec 3;9(12):602. doi: 10.3390/genes9120602 (PMC6316516; doi:10.3390/genes9120602)
Supplement: Supplementary file 1 [file genes-09-00602-s001.zip › Supplementary_file_1_TYoshida.pdf]

>Region1_Col
ATGGATCATATGTTTCCAAAAGGTAAAACTTTCCCCTTCCTTTCTAGGTATGTGGGTGTT
AGCGTGCCAGATATAACTCAATCGATGTGGTTCATCATCTTCTTTCAATACTTCTGGATG
ACCCTTTGTACCTGTATTCAGGAACATAAACTTCATAAACAAAGAAACCGAGTTCGAACT
GAATTTTGTTTTGT

>Region1_Ler
ATGGATCATATGTTTCCAAAAGGTAAgACTTTCCCCTTCCTTTCTAGGTATGTGGGTGTT
AGCcTGCCAGATATAACTCAATCGATGcGGTTCgTCATCTTCTTTCAGTACTTCTGGATGA
CCCTTCGTACCTGTATTCAGGAACATAAACTTCATAATCAAAGAAACCGAGTTCGaACTG
AATTTTGTTTTGT

>Region2_Col
GGAAAAAAGAAGGGCTGTTATGTAAAGATGGAAGAGTGGCGTCTCGAATCAGTCAAGC
TTTCTCCCAATTTCTTCTATATTTTCATTTGTTTCTCTATTTAGCACAGAAAACTCATCTCG
GTCTTATCAGATGGTATCAGAGCCTTCGATCTTGGTTAAAAACGTACCGTCAAGCAAGA
AACGGAACCGTAAACTAGCCCAACATTCTCGGAAAACTAAAGAATCGAGGGTCCCCCA
ACGAGTAAGGGAGTGAGTCTGTC

>Region2_Ler
GGAAAAAAGAAGGGCTGTTATGTAAAGATGGAAGAGTGGCGTCTCGAATCAGTCAAGC
TTTCTCCCAATTTCTTCTATATTTTCATTTGTTTCTCTATTTAGCACAGAAAACTCATCTCG
GTCTTATCAGATGGTATCAGAGCCTTCGGTCTTGGTTAAAAACGTACCGTCAAGCAAGA
AACGGAACCGTAAACTAGCCCAACATTCTCGGAAAACTAAAAAATCGAGGGTCCCCCA
ACGAGTAAGGGAGTGAGTCTGTC
