# Supplementary material for: DNA Methylation Diversification at the Integrated Organellar DNA-Like Sequence
Source: Genes (Basel). 2018 Dec 3;9(12):602. doi: 10.3390/genes9120602 (PMC6316516; doi:10.3390/genes9120602)
Supplement: Supplementary file 1 [file genes-09-00602-s001.zip › Supplementary_file_2_TYoshida.pdf]

```
# Supplemental File 2
# Commands of data analysis

# Trimming of Fastq file
java -jar trimmomatic-0.35.jar PE -threads 24 -trimlog file.log R1.fastq.gz R2.fastq.gz
R1_paired.fastq.gz R1_unpaired.fastq.gz R2_paired.fastq.gz R2_unpaired.fastq.gz
ILLUMINACLIP:adapter.fasta:2:30:10 HEADCROP:25 TRAILING:3 SLIDINGWINDOW:4:15
MINLEN:123


# Reference sequence preparation
bismark_genome_preparation --bowtie2 --vervose /path/to/bismark_ref_b2/


# Mapping of reads
bismark --bowtie2 -N 1 -L 20 -e 90 -p 4 -o /path/to/result/dir /path/to/bismark_ref_b2/ -1
/path/to/R1_paired.fastq.gz -2 /path/to/R2_paired.fastq.gz


# Methylation data extraction
samtools view -h results_bsmrk_bt2_pe.bam > results_bsmrk_bt2_pe.sam

bismark_methylation_extractor -p --no_overlap --cytosine_report --CX -o out_dir --
genome_folder /path/to/bismark_ref_b2 results_bsmrk_bt2_pe.sam
```
