# Supplementary material for: DNA Methylation Diversification at the Integrated Organellar DNA-Like Sequence
Source: Genes (Basel). 2018 Dec 3;9(12):602. doi: 10.3390/genes9120602 (PMC6316516; doi:10.3390/genes9120602)
Supplement: Supplementary file 1 [file genes-09-00602-s001.zip › Supplementary_Tables_revise1.pdf]

Supplementary Table S1   Primers used in the amplicon-Seq

Primer Name	Primer sequence (5' to 3')	Description
**Region 1:**		
overhang_327F	TCGTCGGCAGCGTCAGATGTGTATAAGAGACAGATGGATYATATGTTTYYAAAAGGTA	PCR using bisulfite-modified gDNA for region 1
overhang_520R	GTCTCGTGGGCTCGGAGATGTGTATAAGAGACAGACAAAACAAAATTCARTTCRAACTC	PCR using bisulfite-modified gDNA for region1
**Region 2:**		
oh_numt1381_136F	TCGTCGGCAGCGTCAGATGTGTATAAGAGACAGGGAAAAAAGAAGGGYTGTTATGTAA	PCR using bisulfite-modified gDNA for region 2
oh_numt1381_395R	GTCTCGTGGGCTCGGAGATGTGTATAAGAGACAGRACARACTCACTCCCTTACTC	PCR using bisulfite-modified gDNA for region2

Supplementary Table S2 Position and motif (CpG, CpHpG, and CpHpH) in analyzed sequence

position	motif	Col	Ler
**Resion 1:**			
29	CHH	CTT	CTT
33	CHH	CCC	CCC
34	CHH	CCC	CCC
35	CHH	CCT	CCT
36	CHH	CTT	CTT
39	CHH	CCT	CCT
40	CHH	CTT	CTT
44	CHH	CTA	CTA
67	CHH	CCA	CCA
68	CHG	CAG	CAG
77	CHH	CTC	CTC
79	CHH	CAA	CAA
83	CG	CGA	CGA
96	CHH	CAT	CAT
99	CHH	CTT	CTT
102	CHH	CTT	CTT
111	CHH	CTT	CTT
114	CHG	CTG	CTG
122	CHH	CCC	CCC
123	CHH	CCT	CCT
124	CHH	CTT	CTT
131	CHH	CCT	CCT
132	CHG	CTG	CTG
139	CHG	CAG	CAG
145	CHH	CAT	CAT
151	CHH	CTT	CTT
154	CHH	CAT	CAT
160	CHH	CAA	CAA
168	CHG	CCG	CCG
169	CG	CGA	CGA
**Resion 2:**			
40	CG	CGT	CGT

45	CG	CGA	CGA
119	CG	CGG	CGG
147	CG	CGA	CGG
163	CG	CGT	CGT
168	CG	CGT	CGT
182	CG	CGG	CGG
188	CG	CGT	CGT
209	CG	CGG	CGG
225	CG	CGA	CGA
50	CHG	CAG	CAG
105	CHG	CAG	CAG
128	CHG	CAG	CAG
138	CHG	CAG	CAG
167	CHG	CCG	CCG
187	CHG	CCG	CCG
43	CHH	CTC	CTC
54	CHH	CAA	CAA
58	CHH	CTT	CTT
62	CHH	CTC	CTC
64	CHH	CCC	CCC
65	CHH	CCA	CCA
66	CHH	CAA	CAA
72	CHH	CTT	CTT
75	CHH	CTA	CTA
84	CHH	CAT	CAT
93	CHH	CTC	CTC
95	CHH	CTA	CTA
103	CHH	CAC	CAC
112	CHH	CTC	CTC
114	CHH	CAT	CAT
117	CHH	CTC	CTC
123	CHH	CTT	CTT
143	CHH	CCT	CCT
144	CHH	CTT	CTT
151	CHH	CTT	CTT
171	CHH	CAA	CAA

175	CHH	CAA	CAA
194	CHH	CTA	CTA
198	CHH	CCC	CCC
199	CHH	CCA	CCA
200	CHH	CAA	CAA
203	CHH	CAT	CAT
207	CHH	CTC	CTC
216	CHH	CTA	CTA
232	CHH	CCC	CCC
233	CHH	CCC	CCC
234	CHH	CCC	CCC
235	CHH	CCA	CCA

Supplementary Table S3   Mapped data of bisulfite amplicon-Seq

Sample	locus	genotype	Col_methylated	Col_unmethylated	Ler_methylated	Ler_unmethylated	Total_Mapped	Name
B-C12	Region 1	CC	60584	145460	232	653	206929	Col_ind1
B-C12	Region 2	CC	98313	232934	969	2508	334724	Col_ind1
B-D12	Region 1	CC	55795	200195	224	2631	258845	Col_ind2
B-D12	Region 2	CC	112102	278500	1309	3230	395141	Col_ind2
A-A01	Region 2	CL	44079	101759	35405	101772	283015	ColLer_ind1
A-B02	Region 2	CL	27517	61918	20907	59371	169713	ColLer_ind10
A-C02	Region 2	CL	22254	51441	17959	51875	143529	ColLer_ind11
A-D02	Region 2	CL	27087	61370	21472	61209	171138	ColLer_ind12
A-E02	Region 2	CL	22159	52310	16620	50327	141416	ColLer_ind13
A-F02	Region 2	CL	27462	63898	22108	61872	175340	ColLer_ind14
A-G02	Region 2	CL	37784	87281	27738	86812	239615	ColLer_ind15
A-H02	Region 2	CL	25411	57560	19549	58191	160711	ColLer_ind16
A-A03	Region 2	CL	42289	102805	34036	104544	283674	ColLer_ind17
A-B03	Region 2	CL	44826	103991	34717	101986	285520	ColLer_ind18
A-C03	Region 2	CL	46478	115605	33176	110575	305834	ColLer_ind19
A-B01	Region 2	CL	48695	115014	37770	113170	314649	ColLer_ind2
A-C01	Region 2	CL	51807	121478	38139	117254	328678	ColLer_ind3
A-D01	Region 2	CL	44119	105375	33898	104757	288149	ColLer_ind4
A-E01	Region 2	CL	38749	88734	30323	92707	250513	ColLer_ind5
A-F01	Region 2	CL	38223	92371	32065	94984	257643	ColLer_ind6
A-G01	Region 2	CL	40495	95772	34996	94181	265444	ColLer_ind7

A-H01	Region 2	CL	39827	97472	33571	113183	284053	ColLer_ind8
A-A02	Region 2	CL	23428	53804	17988	59684	154904	ColLer_ind9
A-A01	Region 1	CL	4350	77063	1879	107518	190810	ColLer_ind1
A-B02	Region 1	CL	3057	47065	676	58933	109731	ColLer_ind10
A-C02	Region 1	CL	2860	40851	915	45609	90235	ColLer_ind11
A-D02	Region 1	CL	2427	51020	614	55330	109391	ColLer_ind12
A-E02	Region 1	CL	2988	35249	545	42977	81759	ColLer_ind13
A-F02	Region 1	CL	2919	51389	1210	54813	110331	ColLer_ind14
A-G02	Region 1	CL	2451	47494	667	62599	113211	ColLer_ind15
A-H02	Region 1	CL	2940	35741	1160	44241	84082	ColLer_ind16
A-A03	Region 1	CL	3629	84739	2391	80411	171170	ColLer_ind17
A-B03	Region 1	CL	4828	74434	1422	89430	170114	ColLer_ind18
A-C03	Region 1	CL	7081	80269	951	76220	164521	ColLer_ind19
A-B01	Region 1	CL	3006	58950	797	75426	138179	ColLer_ind2
A-C01	Region 1	CL	5293	61328	733	82484	149838	ColLer_ind3
A-D01	Region 1	CL	5021	53933	1166	63981	124101	ColLer_ind4
A-E01	Region 1	CL	2685	64180	860	70840	138565	ColLer_ind5
A-F01	Region 1	CL	3917	53392	809	67289	125407	ColLer_ind6
A-G01	Region 1	CL	3094	65847	1579	84695	155215	ColLer_ind7
A-H01	Region 1	CL	3730	68304	1324	88335	161693	ColLer_ind8
A-A02	Region 1	CL	3536	35018	1389	46942	86885	ColLer_ind9
A-F05	Region 2	LL	1543	3577	90069	222037	317226	ColLerF1_2_self_ind1
A-G06	Region 2	CL	42476	109653	35838	98112	286079	ColLerF1_2_self_ind10

A-H06	Region 2	LL	1512	3456	75652	195910	276530	ColLerF1_2_self_ind11
A-A07	Region 2	CL	53570	121169	39996	112111	326846	ColLerF1_2_self_ind12
A-B07	Region 2	CL	54916	127019	46555	112188	340678	ColLerF1_2_self_ind13
A-C07	Region 2	CC	100494	249501	1005	2611	353611	ColLerF1_2_self_ind14
A-D07	Region 2	LL	2289	5033	84205	196385	287912	ColLerF1_2_self_ind15
A-E07	Region 2	LL	1531	3610	74705	190042	269888	ColLerF1_2_self_ind16
A-F07	Region 2	LL	2512	5897	94290	220588	323287	ColLerF1_2_self_ind17
A-G07	Region 2	CL	46742	100685	39476	92500	279403	ColLerF1_2_self_ind18
A-H07	Region 2	CL	49164	112794	45124	106334	313416	ColLerF1_2_self_ind19
A-G05	Region 2	LL	1102	2649	68323	176930	249004	ColLerF1_2_self_ind2
A-A08	Region 2	CC	57303	134938	597	1574	194412	ColLerF1_2_self_ind20
A-B08	Region 2	CL	34786	77981	31359	71886	216012	ColLerF1_2_self_ind21
A-C08	Region 2	CL	35091	80500	32360	76253	224204	ColLerF1_2_self_ind22
A-D08	Region 2	CL	28861	64893	24431	59812	177997	ColLerF1_2_self_ind23
A-E08	Region 2	LL	875	2109	51180	128175	182339	ColLerF1_2_self_ind24
A-F08	Region 2	LL	1466	3251	51196	122617	178530	ColLerF1_2_self_ind25
A-G08	Region 2	CL	30834	73186	26961	65020	196001	ColLerF1_2_self_ind26
A-H08	Region 2	CL	27481	62956	25082	57642	173161	ColLerF1_2_self_ind27
A-A09	Region 2	LL	1619	3687	88411	221705	315422	ColLerF1_2_self_ind28
A-B09	Region 2	LL	1905	4121	97499	222072	325597	ColLerF1_2_self_ind29
A-H05	Region 2	CC	78556	191242	1093	2733	273624	ColLerF1_2_self_ind3
A-C09	Region 2	CL	60853	144497	55601	136599	397550	ColLerF1_2_self_ind30
A-A06	Region 2	LL	1355	3130	80903	190057	275445	ColLerF1_2_self_ind4

A-B06	Region 2	LL	1543	3483	83272	208211	296509	ColLerF1_2_self_ind5
A-C06	Region 2	LL	1178	2791	79653	206298	289920	ColLerF1_2_self_ind6
A-D06	Region 2	CC	83902	196421	943	2192	283458	ColLerF1_2_self_ind7
A-E06	Region 2	LL	1085	2642	67192	174185	245104	ColLerF1_2_self_ind8
A-F06	Region 2	CL	43587	103862	39350	95804	282603	ColLerF1_2_self_ind9
A-F05	Region 1	CL	14354	101264	15914	65008	196540	ColLerF1_2_self_ind1
A-G06	Region 1	CL	13508	96522	14236	58830	183096	ColLerF1_2_self_ind10
A-H06	Region 1	LL	55	108	2415	184670	187248	ColLerF1_2_self_ind11
A-A07	Region 1	CL	18788	98027	21350	62645	200810	ColLerF1_2_self_ind12
A-B07	Region 1	CL	16701	64801	11806	74674	167982	ColLerF1_2_self_ind13
A-C07	Region 1	CL	6670	61629	1954	59205	129458	ColLerF1_2_self_ind14
A-D07	Region 1	CL	15731	71450	9783	71177	168141	ColLerF1_2_self_ind15
A-E07	Region 1	CL	5336	72476	1458	70806	150076	ColLerF1_2_self_ind16
A-F07	Region 1	CL	67773	73986	63248	56539	261546	ColLerF1_2_self_ind17
A-G07	Region 1	LL	55	366	138665	128405	267491	ColLerF1_2_self_ind18
A-H07	Region 1	LL	172	543	52908	102014	155637	ColLerF1_2_self_ind19
A-G05	Region 1	LL	36	322	16468	134357	151183	ColLerF1_2_self_ind2
A-A08	Region 1	LL	56	303	8292	101664	110315	ColLerF1_2_self_ind20
A-B08	Region 1	CC	74550	79362	322	739	154973	ColLerF1_2_self_ind21
A-C08	Region 1	CL	11755	42887	5808	44773	105223	ColLerF1_2_self_ind22
A-D08	Region 1	LL	138	382	61470	71871	133861	ColLerF1_2_self_ind23
A-E08	Region 1	CL	23018	47854	22975	34269	128116	ColLerF1_2_self_ind24
A-F08	Region 1	CC	88415	80653	239	435	169742	ColLerF1_2_self_ind25

A-G08	Region 1	LL	67	190	889	122428	123574	ColLerF1_2_self_ind26
A-H08	Region 1	LL	95	199	44965	108278	153537	ColLerF1_2_self_ind27
A-A09	Region 1	CL	18917	86061	18945	53940	177863	ColLerF1_2_self_ind28
A-B09	Region 1	CC	23326	182428	151	739	206644	ColLerF1_2_self_ind29
A-H05	Region 1	LL	67	388	24749	128883	154087	ColLerF1_2_self_ind3
A-C09	Region 1	CL	7531	95796	2594	82513	188434	ColLerF1_2_self_ind30
A-A06	Region 1	CC	118233	135280	213	775	254501	ColLerF1_2_self_ind4
A-B06	Region 1	LL	109	284	1215	129036	130644	ColLerF1_2_self_ind5
A-C06	Region 1	CL	10720	62095	9064	38858	120737	ColLerF1_2_self_ind6
A-D06	Region 1	LL	74	351	14442	134596	149463	ColLerF1_2_self_ind7
A-E06	Region 1	CL	17062	72055	16427	53404	158948	ColLerF1_2_self_ind8
A-F06	Region 1	CL	6012	78594	891	76019	161516	ColLerF1_2_self_ind9
A-D09	Region 2	CL	55361	124020	48631	118158	346170	ColLerF1_4_self_ind1
A-E10	Region 2	CC	90561	223991	922	2398	317872	ColLerF1_4_self_ind10
A-F10	Region 2	CL	49764	118443	39308	112080	319595	ColLerF1_4_self_ind11
A-G10	Region 2	CL	47816	104603	39773	95721	287913	ColLerF1_4_self_ind12
A-H10	Region 2	CL	33979	77628	29224	75055	215886	ColLerF1_4_self_ind13
A-A11	Region 2	CL	46456	120116	41118	116717	324407	ColLerF1_4_self_ind14
A-B11	Region 2	CL	50083	120313	41645	116399	328440	ColLerF1_4_self_ind15
A-C11	Region 2	CC	105719	230058	669	1585	338031	ColLerF1_4_self_ind16
A-D11	Region 2	CL	44399	101146	38254	97995	281794	ColLerF1_4_self_ind17
A-E11	Region 2	CL	47087	108480	37947	106678	300192	ColLerF1_4_self_ind18
A-F11	Region 2	CL	50124	124520	42342	122607	339593	ColLerF1_4_self_ind19

A-E09	Region 2	CC	102735	254051	1160	2748	360694	ColLerF1_4_self_ind2
A-G11	Region 2	CL	48603	111600	40541	107229	307973	ColLerF1_4_self_ind20
A-H11	Region 2	CL	44342	99833	38592	105330	288097	ColLerF1_4_self_ind21
A-A12	Region 2	CC	96618	236042	812	1888	335360	ColLerF1_4_self_ind22
A-B12	Region 2	CC	109253	255827	831	1998	367909	ColLerF1_4_self_ind23
A-C12	Region 2	CL	60153	129991	49130	127069	366343	ColLerF1_4_self_ind24
A-D12	Region 2	CC	96466	245883	892	2431	345672	ColLerF1_4_self_ind25
A-E12	Region 2	CL	49994	114816	41275	106332	312417	ColLerF1_4_self_ind26
A-F12	Region 2	CC	106690	264598	1324	3261	375873	ColLerF1_4_self_ind27
A-G12	Region 2	CC	106500	262134	1024	2443	372101	ColLerF1_4_self_ind28
A-F09	Region 2	CL	53549	128982	46750	123792	353073	ColLerF1_4_self_ind3
A-G09	Region 2	LL	1284	3211	88527	235378	328400	ColLerF1_4_self_ind4
A-H09	Region 2	CL	48267	115671	40194	107954	312086	ColLerF1_4_self_ind5
A-A10	Region 2	CL	42697	104009	34731	99431	280868	ColLerF1_4_self_ind6
A-B10	Region 2	CC	78380	183031	893	2195	264499	ColLerF1_4_self_ind7
A-C10	Region 2	CL	49194	120843	42131	113639	325807	ColLerF1_4_self_ind8
A-D10	Region 2	LL	1464	3506	80606	206898	292474	ColLerF1_4_self_ind9
A-D09	Region 1	CL	17419	93678	12483	79646	203226	ColLerF1_4_self_ind1
A-E10	Region 1	CL	6029	90832	3162	67914	167937	ColLerF1_4_self_ind10
A-F10	Region 1	LL	83	435	1834	160359	162711	ColLerF1_4_self_ind11
A-G10	Region 1	CL	19069	62392	20243	81138	182842	ColLerF1_4_self_ind12
A-H10	Region 1	CL	26118	48532	20432	42826	137908	ColLerF1_4_self_ind13
A-A11	Region 1	LL	100	523	5175	145448	151246	ColLerF1_4_self_ind14

A-B11	Region 1	LL	44	217	56122	158974	215357	ColLerF1_4_self_ind15
A-C11	Region 1	CL	3149	73891	1014	81667	159721	ColLerF1_4_self_ind16
A-D11	Region 1	CL	8533	61943	4093	67499	142068	ColLerF1_4_self_ind17
A-E11	Region 1	CL	5274	74844	949	75087	156154	ColLerF1_4_self_ind18
A-F11	Region 1	CL	4077	71314	1269	86042	162702	ColLerF1_4_self_ind19
A-E09	Region 1	CL	29971	94935	26239	79474	230619	ColLerF1_4_self_ind2
A-G11	Region 1	CC	32029	162393	156	661	195239	ColLerF1_4_self_ind20
A-H11	Region 1	CC	102639	121279	332	983	225233	ColLerF1_4_self_ind21
A-A12	Region 1	LL	111	312	4040	159511	163974	ColLerF1_4_self_ind22
A-B12	Region 1	CL	13493	60087	10279	86937	170796	ColLerF1_4_self_ind23
A-C12	Region 1	CL	41754	67675	35880	75860	221169	ColLerF1_4_self_ind24
A-D12	Region 1	LL	137	320	12456	142560	155473	ColLerF1_4_self_ind25
A-E12	Region 1	CC	125849	153833	336	719	280737	ColLerF1_4_self_ind26
A-F12	Region 1	CL	20818	70469	15749	109072	216108	ColLerF1_4_self_ind27
A-G12	Region 1	CL	10583	88516	6462	95309	200870	ColLerF1_4_self_ind28
A-F09	Region 1	CL	20070	101992	12308	93960	228330	ColLerF1_4_self_ind3
A-G09	Region 1	CL	72944	77390	77114	76969	304417	ColLerF1_4_self_ind4
A-H09	Region 1	CC	74333	87432	388	672	162825	ColLerF1_4_self_ind5
A-A10	Region 1	CL	17058	57840	12627	80115	167640	ColLerF1_4_self_ind6
A-B10	Region 1	CC	65244	114753	197	603	180797	ColLerF1_4_self_ind7
A-C10	Region 1	CL	3063	85111	1481	62078	151733	ColLerF1_4_self_ind8
A-D10	Region 1	CC	87367	103385	202	494	191448	ColLerF1_4_self_ind9
B-E12	Region 1	LL	36	192	1148	129467	130843	Ler_ind1

B-E12	Region 2	LL	1488	4303	47622	202958	256371	Ler_ind1
B-F12	Region 1	LL	128	525	1567	179963	182183	Ler_ind2
B-F12	Region 2	LL	1567	4952	57436	331267	395222	Ler_ind2
A-D03	Region 1	CL	7856	71194	2700	75205	156955	LerCol_ind1
A-D03	Region 2	CL	41089	98848	28616	100768	269321	LerCol_ind1
A-E04	Region 1	CL	2249	22389	259	18789	43686	LerCol_ind10
A-E04	Region 2	CL	14603	34564	9908	27498	86573	LerCol_ind10
A-F04	Region 1	CL	5439	71533	1630	82884	161486	LerCol_ind11
A-F04	Region 2	CL	42929	103565	29634	106816	282944	LerCol_ind11
A-G04	Region 1	CL	7718	58692	1505	59378	127293	LerCol_ind12
A-G04	Region 2	CL	34121	79189	22993	86043	222346	LerCol_ind12
A-H04	Region 1	CL	4756	71480	1691	82057	159984	LerCol_ind13
A-H04	Region 2	CL	35232	85702	26961	87206	235101	LerCol_ind13
A-A05	Region 1	CL	6375	74822	1660	75509	158366	LerCol_ind14
A-A05	Region 2	CL	48034	113041	33809	123317	318201	LerCol_ind14
A-B05	Region 1	CL	5759	68585	1561	77996	153901	LerCol_ind15
A-B05	Region 2	CL	43448	102549	38913	103505	288415	LerCol_ind15
A-C05	Region 1	CL	4833	71676	1023	63616	141148	LerCol_ind16
A-C05	Region 2	CL	45299	110926	33607	106938	296770	LerCol_ind16
A-D05	Region 1	CL	7813	86431	1278	91731	187253	LerCol_ind17
A-D05	Region 2	CL	48669	111892	32830	112660	306051	LerCol_ind17
A-E05	Region 1	CL	5143	78621	1019	67280	152063	LerCol_ind18
A-E05	Region 2	CL	40309	94195	29815	94454	258773	LerCol_ind18

A-E03	Region 1	CL	7440	81343	1776	67976	158535	LerCol_ind2
A-E03	Region 2	CL	42947	103156	28406	105882	280391	LerCol_ind2
A-F03	Region 1	CL	4979	72681	829	85519	164008	LerCol_ind3
A-F03	Region 2	CL	45722	104441	33152	106407	289722	LerCol_ind3
A-G03	Region 1	CL	4218	65502	682	70688	141090	LerCol_ind4
A-G03	Region 2	CL	42958	96987	28498	105148	273591	LerCol_ind4
A-H03	Region 1	CL	5642	93405	1614	117658	218319	LerCol_ind5
A-H03	Region 2	CL	41609	95677	35519	93205	266010	LerCol_ind5
A-A04	Region 1	CL	4950	59617	1020	67066	132653	LerCol_ind6
A-A04	Region 2	CL	38620	92369	27620	101169	259778	LerCol_ind6
A-B04	Region 1	CL	6248	66723	1512	76779	151262	LerCol_ind7
A-B04	Region 2	CL	40901	98560	27826	102908	270195	LerCol_ind7
A-C04	Region 1	CL	4679	42219	2137	34795	83830	LerCol_ind8
A-C04	Region 2	CL	19598	48555	13389	46828	128370	LerCol_ind8
A-D04	Region 1	CL	4323	60683	590	65584	131180	LerCol_ind9
A-D04	Region 2	CL	38047	91629	31721	87555	248952	LerCol_ind9
B-A01	Region 2	CL	44835	113023	39037	99523	296418	LerColF1_3_self_ind1
B-B02	Region 2	CL	35868	88309	32002	82792	238971	LerColF1_3_self_ind10
B-C02	Region 2	CL	37909	95174	33211	85935	252229	LerColF1_3_self_ind11
B-D02	Region 2	LL	1468	3204	64176	152887	221735	LerColF1_3_self_ind12
B-E02	Region 2	CL	35612	91305	31198	84000	242115	LerColF1_3_self_ind13
B-F02	Region 2	CL	38276	92200	36752	90890	258118	LerColF1_3_self_ind14
B-G02	Region 2	CC	61068	134173	669	1543	197453	LerColF1_3_self_ind15

B-H02	Region 2	CL	20295	46897	17990	44481	129663	LerColF1_3_self_ind16
B-A03	Region 2	LL	1383	3042	91146	202530	298101	LerColF1_3_self_ind17
B-B03	Region 2	LL	1554	3430	82818	183684	271486	LerColF1_3_self_ind18
B-C03	Region 2	CL	48049	110404	41182	98333	297968	LerColF1_3_self_ind19
B-B01	Region 2	CC	98752	246282	1258	3180	349472	LerColF1_3_self_ind2
B-D03	Region 2	CL	42644	96168	37972	88504	265288	LerColF1_3_self_ind20
B-E03	Region 2	LL	1164	2791	74233	190118	268306	LerColF1_3_self_ind21
B-F03	Region 2	LL	1713	3737	83414	186680	275544	LerColF1_3_self_ind22
B-G03	Region 2	CL	42650	98804	35898	86208	263560	LerColF1_3_self_ind23
B-H03	Region 2	CL	43577	102570	38517	94995	279659	LerColF1_3_self_ind24
B-A04	Region 2	LL	1142	2539	67489	164651	235821	LerColF1_3_self_ind25
B-B04	Region 2	LL	1237	2899	71695	184840	260671	LerColF1_3_self_ind26
B-C04	Region 2	CL	41350	103504	37530	96755	279139	LerColF1_3_self_ind27
B-D04	Region 2	CC	71493	168390	846	1952	242681	LerColF1_3_self_ind28
B-E04	Region 2	LL	1169	2730	74362	183012	261273	LerColF1_3_self_ind29
B-C01	Region 2	LL	1526	3587	97680	238423	341216	LerColF1_3_self_ind3
B-F04	Region 2	CC	72441	173600	875	2218	249134	LerColF1_3_self_ind30
B-D01	Region 2	CC	98545	230669	910	2230	332354	LerColF1_3_self_ind4
B-E01	Region 2	CL	50325	116834	41770	99053	307982	LerColF1_3_self_ind5
B-F01	Region 2	CL	55606	128080	51979	124991	360656	LerColF1_3_self_ind6
B-G01	Region 2	CC	91724	221207	1020	2547	316498	LerColF1_3_self_ind7
B-H01	Region 2	LL	1938	4671	87231	210225	304065	LerColF1_3_self_ind8
B-A02	Region 2	CL	37082	89030	35337	85499	246948	LerColF1_3_self_ind9

B-A01	Region 1	CC	80156	169461	221	1089	250927	LerColF1_3_self_ind1
B-B02	Region 1	CL	3398	36212	1531	47528	88669	LerColF1_3_self_ind10
B-C02	Region 1	CL	33530	59622	36363	51661	181176	LerColF1_3_self_ind11
B-D02	Region 1	CL	24885	53013	22375	52585	152858	LerColF1_3_self_ind12
B-E02	Region 1	LL	102	254	21366	82921	104643	LerColF1_3_self_ind13
B-F02	Region 1	CL	4864	64884	3062	63138	135948	LerColF1_3_self_ind14
B-G02	Region 1	LL	131	325	69654	55749	125859	LerColF1_3_self_ind15
B-H02	Region 1	LL	117	243	20594	50927	71881	LerColF1_3_self_ind16
B-A03	Region 1	CL	25173	57431	16105	91169	189878	LerColF1_3_self_ind17
B-B03	Region 1	CL	13418	19700	10121	22047	65286	LerColF1_3_self_ind18
B-C03	Region 1	CL	30794	59947	21251	57818	169810	LerColF1_3_self_ind19
B-B01	Region 1	CL	10575	79757	5425	96429	192186	LerColF1_3_self_ind2
B-D03	Region 1	CL	12248	57609	6960	61614	138431	LerColF1_3_self_ind20
B-E03	Region 1	CC	75364	133791	139	321	209615	LerColF1_3_self_ind21
B-F03	Region 1	CL	7003	72184	1469	79650	160306	LerColF1_3_self_ind22
B-G03	Region 1	CC	91952	128711	380	686	221729	LerColF1_3_self_ind23
B-H03	Region 1	CL	6478	82967	6715	52091	148251	LerColF1_3_self_ind24
B-A04	Region 1	CC	81851	87102	190	586	169729	LerColF1_3_self_ind25
B-B04	Region 1	LL	160	326	17713	118020	136219	LerColF1_3_self_ind26
B-C04	Region 1	CL	1810	40958	424	39247	82439	LerColF1_3_self_ind27
B-D04	Region 1	CC	47102	89961	173	394	137630	LerColF1_3_self_ind28
B-E04	Region 1	CL	5504	70875	1362	58169	135910	LerColF1_3_self_ind29
B-C01	Region 1	CL	8379	100992	5699	76128	191198	LerColF1_3_self_ind3

B-F04	Region 1	CC	52342	82251	166	439	135198	LerColF1_3_self_ind30
B-D01	Region 1	CL	15167	86392	6707	88120	196386	LerColF1_3_self_ind4
B-E01	Region 1	CL	15504	91754	15321	66646	189225	LerColF1_3_self_ind5
B-F01	Region 1	CC	107431	149635	224	724	258014	LerColF1_3_self_ind6
B-G01	Region 1	CC	75303	147705	241	501	223750	LerColF1_3_self_ind7
B-H01	Region 1	CL	8641	89648	4128	107987	210404	LerColF1_3_self_ind8
B-A02	Region 1	LL	30	263	3179	137099	140571	LerColF1_3_self_ind9
B-G04	Region 2	CL	37484	95656	33452	89844	256436	LerColF1_4_self_ind1
B-A06	Region 2	LL	1375	3205	81240	211019	296839	LerColF1_4_self_ind11
B-B06	Region 2	LL	1254	2798	85162	206682	295896	LerColF1_4_self_ind12
B-C06	Region 2	CL	49371	123535	44697	114976	332579	LerColF1_4_self_ind13
B-D06	Region 2	CL	76322	185016	62356	183822	507516	LerColF1_4_self_ind14
B-E06	Region 2	CL	44592	106905	38674	97076	287247	LerColF1_4_self_ind15
B-F06	Region 2	CL	48383	117708	43652	107225	316968	LerColF1_4_self_ind16
B-G06	Region 2	CC	51973	124361	644	1581	178559	LerColF1_4_self_ind17
B-H06	Region 2	CL	41645	98712	37881	92325	270563	LerColF1_4_self_ind18
B-A07	Region 2	CC	83026	220285	1102	3038	307451	LerColF1_4_self_ind19
B-H04	Region 2	CL	28481	73202	26963	69664	198310	LerColF1_4_self_ind2
B-B07	Region 2	CL	42939	104725	38575	97043	283282	LerColF1_4_self_ind20
B-C07	Region 2	LL	1500	3911	76064	230615	312090	LerColF1_4_self_ind21
B-D07	Region 2	LL	2120	5358	76453	225206	309137	LerColF1_4_self_ind22
B-E07	Region 2	CL	40227	111192	36960	93523	281902	LerColF1_4_self_ind23
B-F07	Region 2	CL	45340	114083	41336	106336	307095	LerColF1_4_self_ind24

B-G07	Region 2	LL	1384	3399	69272	185501	259556	LerColF1_4_self_ind25
B-H07	Region 2	LL	1642	4062	71436	205000	282140	LerColF1_4_self_ind26
B-A08	Region 2	CC	59729	156476	691	1808	218704	LerColF1_4_self_ind27
B-B08	Region 2	CC	55972	134710	556	1521	192759	LerColF1_4_self_ind28
B-C08	Region 2	LL	1036	2390	58663	138252	200341	LerColF1_4_self_ind29
B-A05	Region 2	CC	81823	190551	897	2232	275503	LerColF1_4_self_ind3
B-D08	Region 2	CL	33823	78798	28638	69359	210618	LerColF1_4_self_ind30
B-B05	Region 2	CC	89948	212744	748	1896	305336	LerColF1_4_self_ind4
B-C05	Region 2	CL	45599	107295	40068	105722	298684	LerColF1_4_self_ind5
B-D05	Region 2	LL	1931	4593	97491	234009	338024	LerColF1_4_self_ind6
B-E05	Region 2	CL	44506	104878	41965	102287	293636	LerColF1_4_self_ind7
B-F05	Region 2	LL	1787	4047	83655	208749	298238	LerColF1_4_self_ind8
B-G05	Region 2	CL	42766	96421	37193	86521	262901	LerColF1_4_self_ind9
B-G04	Region 1	LL	14	84	2059	103358	105515	LerColF1_4_self_ind1
B-A06	Region 1	LL	76	346	1101	111813	113336	LerColF1_4_self_ind11
B-B06	Region 1	CL	20353	64840	13064	70035	168292	LerColF1_4_self_ind12
B-C06	Region 1	CC	47269	124509	124	446	172348	LerColF1_4_self_ind13
B-D06	Region 1	CL	5674	63484	3805	50006	122969	LerColF1_4_self_ind14
B-E06	Region 1	CL	5186	67292	1707	54737	128922	LerColF1_4_self_ind15
B-F06	Region 1	CC	49257	113963	163	472	163855	LerColF1_4_self_ind16
B-G06	Region 1	CL	7475	45845	5971	32841	92132	LerColF1_4_self_ind17
B-H06	Region 1	LL	67	261	13964	138008	152300	LerColF1_4_self_ind18
B-A07	Region 1	CL	17718	82150	16938	77895	194701	LerColF1_4_self_ind19

B-H04	Region 1	CC	22633	91073	164	791	114661	LerColF1_4_self_ind2
B-B07	Region 1	CL	21935	79636	21313	56552	179436	LerColF1_4_self_ind20
B-C07	Region 1	CL	10019	69051	5390	57492	141952	LerColF1_4_self_ind21
B-D07	Region 1	LL	12	216	978	118866	120072	LerColF1_4_self_ind22
B-E07	Region 1	LL	34	92	4115	100539	104780	LerColF1_4_self_ind23
B-F07	Region 1	CL	5594	80586	886	64588	151654	LerColF1_4_self_ind24
B-G07	Region 1	CC	57180	144085	172	599	202036	LerColF1_4_self_ind25
B-H07	Region 1	CL	9274	73722	3392	72557	158945	LerColF1_4_self_ind26
B-A08	Region 1	CC	23386	105456	65	606	129513	LerColF1_4_self_ind27
B-B08	Region 1	LL	44	217	1407	89602	91270	LerColF1_4_self_ind28
B-C08	Region 1	CL	13650	47597	9622	45689	116558	LerColF1_4_self_ind29
B-A05	Region 1	CL	6802	65809	2548	58339	133498	LerColF1_4_self_ind3
B-D08	Region 1	CL	11162	34314	7514	35115	88105	LerColF1_4_self_ind30
B-B05	Region 1	CL	22643	61984	20032	64645	169304	LerColF1_4_self_ind4
B-C05	Region 1	LL	63	232	7483	102118	109896	LerColF1_4_self_ind5
B-D05	Region 1	LL	63	263	5812	97952	104090	LerColF1_4_self_ind6
B-E05	Region 1	CC	60919	78723	169	320	140131	LerColF1_4_self_ind7
B-F05	Region 1	CC	61921	110176	158	626	172881	LerColF1_4_self_ind8
B-G05	Region 1	CL	11358	59425	8316	52324	131423	LerColF1_4_self_ind9

Supplementary Table S4 Genotype of F_2 populations

Region	Population		Genotype			Total
			Col/Col	Col/L*er*	L*er*/L*er*	
**Region 1**	F_2 (Col x L*er* ind.2 Self)	Observed	4	15	11	30
		Expected	7.5	15	7.5	30
		Chi-square		3.27		
		p-value		0.195		
	F_2 (Col x L*er* ind.4 Self)	Observed	6	17	5	28
		Expected	7	14	7	28
		Chi-square		1.36		
		p-value		0.507		
	F_2 (L*er* x Col ind.3 Self)	Observed	8	17	5	30
		Expected	7.5	15	7.5	30
		Chi-square		1.13		
		p-value		0.567		
	F_2 (L*er* x col ind.4 Self)	Observed	7	14	8	29
		Expected	7.25	14.5	7.25	29
		Chi-square		0.10		
		p-value		0.950		
**Region 2**	F_2 (Col x L*er* ind.2 Self)	Observed	4	12	14	30
		Expected	7.5	15	7.5	30
		Chi-square		7.87		
		p-value		0.0196		
	F_2 (Col x L*er* ind.4 Self)	Observed	9	17	2	28
		Expected	7	14	7	28
		Chi-square		4.79		
		p-value		0.0914		
	F_2 (L*er* x Col ind.3 Self)	Observed	6	14	10	30
		Expected	7.5	15	7.5	30
		Chi-square		1.20		
		p-value		0.549		
	F_2 (L*er* x col ind.4 Self)	Observed	6	14	9	29
		Expected	7.25	14.5	7.25	29
		Chi-square		0.66		
		p-value		0.721		

Supplementary Table S5 Level of DNA methylation

Methylation level	Motif	Allele	Genotype	Locus	Name	Group
0.717263581	CG	Col	CC	Region 1	B-C12	Parent
0.420333164	CHG	Col	CC	Region 1	B-C12	Parent
0.211483334	CHH	Col	CC	Region 1	B-C12	Parent
0.879665171	CG	Col	CC	Region 2	B-C12	Parent
0.518320083	CHG	Col	CC	Region 2	B-C12	Parent
0.079522881	CHH	Col	CC	Region 2	B-C12	Parent
0.624003629	CG	Col	CC	Region 1	B-D12	Parent
0.339938207	CHG	Col	CC	Region 1	B-D12	Parent
0.140719319	CHH	Col	CC	Region 1	B-D12	Parent
0.841668755	CG	Col	CC	Region 2	B-D12	Parent
0.506945316	CHG	Col	CC	Region 2	B-D12	Parent
0.078664375	CHH	Col	CC	Region 2	B-D12	Parent
0.005520287	CG	Ler	LL	Region 1	B-E12	Parent
0.008696125	CHG	Ler	LL	Region 1	B-E12	Parent
0.00900543	CHH	Ler	LL	Region 1	B-E12	Parent
0.660951489	CG	Ler	LL	Region 2	B-E12	Parent
0.301104342	CHG	Ler	LL	Region 2	B-E12	Parent
0.026652907	CHH	Ler	LL	Region 2	B-E12	Parent
0.008155147	CG	Ler	LL	Region 1	B-F12	Parent
0.008455997	CHG	Ler	LL	Region 1	B-F12	Parent
0.008674949	CHH	Ler	LL	Region 1	B-F12	Parent
0.564532392	CG	Ler	LL	Region 2	B-F12	Parent
0.175210807	CHG	Ler	LL	Region 2	B-F12	Parent
0.016405283	CHH	Ler	LL	Region 2	B-F12	Parent
0.374442191	CG	Col	CL	Region 1	A-A01	ColLer
0.076467765	CHG	Col	CL	Region 1	A-A01	ColLer
0.017163441	CHH	Col	CL	Region 1	A-A01	ColLer
0.042602378	CG	Ler	CL	Region 1	A-A01	ColLer
0.016430705	CHG	Ler	CL	Region 1	A-A01	ColLer
0.010053282	CHH	Ler	CL	Region 1	A-A01	ColLer
0.911756811	CG	Col	CL	Region 2	A-A01	ColLer
0.525425627	CHG	Col	CL	Region 2	A-A01	ColLer
0.076616723	CHH	Col	CL	Region 2	A-A01	ColLer
0.835928314	CG	Ler	CL	Region 2	A-A01	ColLer

0.433097124	CHG	Ler	CL	Region 2	A-A01	ColLer
0.05058821	CHH	Ler	CL	Region 2	A-A01	ColLer
0.332177161	CG	Col	CL	Region 1	A-B01	ColLer
0.072066394	CHG	Col	CL	Region 1	A-B01	ColLer
0.015843559	CHH	Col	CL	Region 1	A-B01	ColLer
0.016844603	CG	Ler	CL	Region 1	A-B01	ColLer
0.012399776	CHG	Ler	CL	Region 1	A-B01	ColLer
0.008819469	CHH	Ler	CL	Region 1	A-B01	ColLer
0.881522682	CG	Col	CL	Region 2	A-B01	ColLer
0.530296219	CHG	Col	CL	Region 2	A-B01	ColLer
0.078008073	CHH	Col	CL	Region 2	A-B01	ColLer
0.805911681	CG	Ler	CL	Region 2	A-B01	ColLer
0.429661705	CHG	Ler	CL	Region 2	A-B01	ColLer
0.048623212	CHH	Ler	CL	Region 2	A-B01	ColLer
0.49950446	CG	Col	CL	Region 1	A-C01	ColLer
0.143318017	CHG	Col	CL	Region 1	A-C01	ColLer
0.022143088	CHH	Col	CL	Region 1	A-C01	ColLer
0.014069264	CG	Ler	CL	Region 1	A-C01	ColLer
0.009046684	CHG	Ler	CL	Region 1	A-C01	ColLer
0.008124858	CHH	Ler	CL	Region 1	A-C01	ColLer
0.904677224	CG	Col	CL	Region 2	A-C01	ColLer
0.524305719	CHG	Col	CL	Region 2	A-C01	ColLer
0.07404391	CHH	Col	CL	Region 2	A-C01	ColLer
0.813446225	CG	Ler	CL	Region 2	A-C01	ColLer
0.412154348	CHG	Ler	CL	Region 2	A-C01	ColLer
0.042592451	CHH	Ler	CL	Region 2	A-C01	ColLer
0.464285714	CG	Col	CL	Region 1	A-D01	ColLer
0.154229407	CHG	Col	CL	Region 1	A-D01	ColLer
0.030927835	CHH	Col	CL	Region 1	A-D01	ColLer
0.045643154	CG	Ler	CL	Region 1	A-D01	ColLer
0.016243323	CHG	Ler	CL	Region 1	A-D01	ColLer
0.011675029	CHH	Ler	CL	Region 1	A-D01	ColLer
0.883788552	CG	Col	CL	Region 2	A-D01	ColLer
0.511262985	CHG	Col	CL	Region 2	A-D01	ColLer
0.076712792	CHH	Col	CL	Region 2	A-D01	ColLer
0.790075124	CG	Ler	CL	Region 2	A-D01	ColLer

0.404676365	CHG	Ler	CL	Region 2	A-D01	ColLer
0.049570836	CHH	Ler	CL	Region 2	A-D01	ColLer
0.296689723	CG	Col	CL	Region 1	A-E01	ColLer
0.046020627	CHG	Col	CL	Region 1	A-E01	ColLer
0.014722001	CHH	Col	CL	Region 1	A-E01	ColLer
0.023063424	CG	Ler	CL	Region 1	A-E01	ColLer
0.011399683	CHG	Ler	CL	Region 1	A-E01	ColLer
0.009008812	CHH	Ler	CL	Region 1	A-E01	ColLer
0.900134177	CG	Col	CL	Region 2	A-E01	ColLer
0.529539921	CHG	Col	CL	Region 2	A-E01	ColLer
0.08165097	CHH	Col	CL	Region 2	A-E01	ColLer
0.80604954	CG	Ler	CL	Region 2	A-E01	ColLer
0.411491507	CHG	Ler	CL	Region 2	A-E01	ColLer
0.046094364	CHH	Ler	CL	Region 2	A-E01	ColLer
0.400230681	CG	Col	CL	Region 1	A-F01	ColLer
0.116101888	CHG	Col	CL	Region 1	A-F01	ColLer
0.023421435	CHH	Col	CL	Region 1	A-F01	ColLer
0.024370861	CG	Ler	CL	Region 1	A-F01	ColLer
0.014822547	CHG	Ler	CL	Region 1	A-F01	ColLer
0.008771525	CHH	Ler	CL	Region 1	A-F01	ColLer
0.880760024	CG	Col	CL	Region 2	A-F01	ColLer
0.507449606	CHG	Col	CL	Region 2	A-F01	ColLer
0.074736579	CHH	Col	CL	Region 2	A-F01	ColLer
0.807843288	CG	Ler	CL	Region 2	A-F01	ColLer
0.435851242	CHG	Ler	CL	Region 2	A-F01	ColLer
0.05027946	CHH	Ler	CL	Region 2	A-F01	ColLer
0.348441247	CG	Col	CL	Region 1	A-G01	ColLer
0.058867925	CHG	Col	CL	Region 1	A-G01	ColLer
0.013359455	CHH	Col	CL	Region 1	A-G01	ColLer
0.077965395	CG	Ler	CL	Region 1	A-G01	ColLer
0.020610058	CHG	Ler	CL	Region 1	A-G01	ColLer
0.008050709	CHH	Ler	CL	Region 1	A-G01	ColLer
0.906805531	CG	Col	CL	Region 2	A-G01	ColLer
0.519126994	CHG	Col	CL	Region 2	A-G01	ColLer
0.071798393	CHH	Col	CL	Region 2	A-G01	ColLer
0.870279892	CG	Ler	CL	Region 2	A-G01	ColLer

0.452770582	CHG	Ler	CL	Region 2	A-G01	ColLer
0.055806036	CHH	Ler	CL	Region 2	A-G01	ColLer
0.360284013	CG	Col	CL	Region 1	A-H01	ColLer
0.074977416	CHG	Col	CL	Region 1	A-H01	ColLer
0.017499052	CHH	Col	CL	Region 1	A-H01	ColLer
0.046413502	CG	Ler	CL	Region 1	A-H01	ColLer
0.016876634	CHG	Ler	CL	Region 1	A-H01	ColLer
0.008044728	CHH	Ler	CL	Region 1	A-H01	ColLer
0.882916578	CG	Col	CL	Region 2	A-H01	ColLer
0.504533524	CHG	Col	CL	Region 2	A-H01	ColLer
0.071187541	CHH	Col	CL	Region 2	A-H01	ColLer
0.762494162	CG	Ler	CL	Region 2	A-H01	ColLer
0.391710709	CHG	Ler	CL	Region 2	A-H01	ColLer
0.037195227	CHH	Ler	CL	Region 2	A-H01	ColLer
0.503442341	CG	Col	CL	Region 1	A-A02	ColLer
0.172636225	CHG	Col	CL	Region 1	A-A02	ColLer
0.0310522	CHH	Col	CL	Region 1	A-A02	ColLer
0.073243647	CG	Ler	CL	Region 1	A-A02	ColLer
0.031360424	CHG	Ler	CL	Region 1	A-A02	ColLer
0.016681299	CHH	Ler	CL	Region 1	A-A02	ColLer
0.913255608	CG	Col	CL	Region 2	A-A02	ColLer
0.523860021	CHG	Col	CL	Region 2	A-A02	ColLer
0.078335256	CHH	Col	CL	Region 2	A-A02	ColLer
0.770072304	CG	Ler	CL	Region 2	A-A02	ColLer
0.392113565	CHG	Ler	CL	Region 2	A-A02	ColLer
0.038483619	CHH	Ler	CL	Region 2	A-A02	ColLer
0.363365353	CG	Col	CL	Region 1	A-B02	ColLer
0.104169373	CHG	Col	CL	Region 1	A-B02	ColLer
0.022617819	CHH	Col	CL	Region 1	A-B02	ColLer
0.019951632	CG	Ler	CL	Region 1	A-B02	ColLer
0.009781701	CHG	Ler	CL	Region 1	A-B02	ColLer
0.009546414	CHH	Ler	CL	Region 1	A-B02	ColLer
0.909974872	CG	Col	CL	Region 2	A-B02	ColLer
0.519963454	CHG	Col	CL	Region 2	A-B02	ColLer
0.085856236	CHH	Col	CL	Region 2	A-B02	ColLer
0.818614296	CG	Ler	CL	Region 2	A-B02	ColLer

0.440924832	CHG	Ler	CL	Region 2	A-B02	ColLer
0.058375259	CHH	Ler	CL	Region 2	A-B02	ColLer
0.440620272	CG	Col	CL	Region 1	A-C02	ColLer
0.089269747	CHG	Col	CL	Region 1	A-C02	ColLer
0.023105239	CHH	Col	CL	Region 1	A-C02	ColLer
0.049129594	CG	Ler	CL	Region 1	A-C02	ColLer
0.02293578	CHG	Ler	CL	Region 1	A-C02	ColLer
0.010740703	CHH	Ler	CL	Region 1	A-C02	ColLer
0.91893864	CG	Col	CL	Region 2	A-C02	ColLer
0.52672347	CHG	Col	CL	Region 2	A-C02	ColLer
0.073433907	CHH	Col	CL	Region 2	A-C02	ColLer
0.831714984	CG	Ler	CL	Region 2	A-C02	ColLer
0.445317079	CHG	Ler	CL	Region 2	A-C02	ColLer
0.047970715	CHH	Ler	CL	Region 2	A-C02	ColLer
0.316666667	CG	Col	CL	Region 1	A-D02	ColLer
0.050773164	CHG	Col	CL	Region 1	A-D02	ColLer
0.018609654	CHH	Col	CL	Region 1	A-D02	ColLer
0.014567821	CG	Ler	CL	Region 1	A-D02	ColLer
0.011684023	CHG	Ler	CL	Region 1	A-D02	ColLer
0.009373334	CHH	Ler	CL	Region 1	A-D02	ColLer
0.9024808	CG	Col	CL	Region 2	A-D02	ColLer
0.519125683	CHG	Col	CL	Region 2	A-D02	ColLer
0.086433848	CHH	Col	CL	Region 2	A-D02	ColLer
0.807057989	CG	Ler	CL	Region 2	A-D02	ColLer
0.439549585	CHG	Ler	CL	Region 2	A-D02	ColLer
0.060551052	CHH	Ler	CL	Region 2	A-D02	ColLer
0.482922611	CG	Col	CL	Region 1	A-E02	ColLer
0.13519496	CHG	Col	CL	Region 1	A-E02	ColLer
0.023864106	CHH	Col	CL	Region 1	A-E02	ColLer
0.023563456	CG	Ler	CL	Region 1	A-E02	ColLer
0.012254902	CHG	Ler	CL	Region 1	A-E02	ColLer
0.009240543	CHH	Ler	CL	Region 1	A-E02	ColLer
0.910748246	CG	Col	CL	Region 2	A-E02	ColLer
0.516897081	CHG	Col	CL	Region 2	A-E02	ColLer
0.071049375	CHH	Col	CL	Region 2	A-E02	ColLer
0.820486668	CG	Ler	CL	Region 2	A-E02	ColLer

0.420039005	CHG	Ler	CL	Region 2	A-E02	ColLer
0.042475674	CHH	Ler	CL	Region 2	A-E02	ColLer
0.373020706	CG	Col	CL	Region 1	A-F02	ColLer
0.075779376	CHG	Col	CL	Region 1	A-F02	ColLer
0.017888804	CHH	Col	CL	Region 1	A-F02	ColLer
0.054533721	CG	Ler	CL	Region 1	A-F02	ColLer
0.02310231	CHG	Ler	CL	Region 1	A-F02	ColLer
0.013211781	CHH	Ler	CL	Region 1	A-F02	ColLer
0.916880342	CG	Col	CL	Region 2	A-F02	ColLer
0.531264017	CHG	Col	CL	Region 2	A-F02	ColLer
0.071166043	CHH	Col	CL	Region 2	A-F02	ColLer
0.858273214	CG	Ler	CL	Region 2	A-F02	ColLer
0.447630193	CHG	Ler	CL	Region 2	A-F02	ColLer
0.048922848	CHH	Ler	CL	Region 2	A-F02	ColLer
0.348414795	CG	Col	CL	Region 1	A-G02	ColLer
0.063793777	CHG	Col	CL	Region 1	A-G02	ColLer
0.016724237	CHH	Col	CL	Region 1	A-G02	ColLer
0.02853067	CG	Ler	CL	Region 1	A-G02	ColLer
0.009762118	CHG	Ler	CL	Region 1	A-G02	ColLer
0.008196721	CHH	Ler	CL	Region 1	A-G02	ColLer
0.897267503	CG	Col	CL	Region 2	A-G02	ColLer
0.533071895	CHG	Col	CL	Region 2	A-G02	ColLer
0.079291543	CHH	Col	CL	Region 2	A-G02	ColLer
0.771232351	CG	Ler	CL	Region 2	A-G02	ColLer
0.429416376	CHG	Ler	CL	Region 2	A-G02	ColLer
0.047123544	CHH	Ler	CL	Region 2	A-G02	ColLer
0.469457497	CG	Col	CL	Region 1	A-H02	ColLer
0.135198921	CHG	Col	CL	Region 1	A-H02	ColLer
0.023767965	CHH	Col	CL	Region 1	A-H02	ColLer
0.070747218	CG	Ler	CL	Region 1	A-H02	ColLer
0.031313606	CHG	Ler	CL	Region 1	A-H02	ColLer
0.012255072	CHH	Ler	CL	Region 1	A-H02	ColLer
0.908898055	CG	Col	CL	Region 2	A-H02	ColLer
0.542589857	CHG	Col	CL	Region 2	A-H02	ColLer
0.080151852	CHH	Col	CL	Region 2	A-H02	ColLer
0.816318833	CG	Ler	CL	Region 2	A-H02	ColLer

0.427370191	CHG	Ler	CL	Region 2	A-H02	ColLer
0.047731009	CHH	Ler	CL	Region 2	A-H02	ColLer
0.291424282	CG	Col	CL	Region 1	A-A03	ColLer
0.054888381	CHG	Col	CL	Region 1	A-A03	ColLer
0.013948725	CHH	Col	CL	Region 1	A-A03	ColLer
0.096393443	CG	Ler	CL	Region 1	A-A03	ColLer
0.041684572	CHG	Ler	CL	Region 1	A-A03	ColLer
0.012081097	CHH	Ler	CL	Region 1	A-A03	ColLer
0.887112517	CG	Col	CL	Region 2	A-A03	ColLer
0.51888175	CHG	Col	CL	Region 2	A-A03	ColLer
0.068886408	CHH	Col	CL	Region 2	A-A03	ColLer
0.795601917	CG	Ler	CL	Region 2	A-A03	ColLer
0.427257722	CHG	Ler	CL	Region 2	A-A03	ColLer
0.045174714	CHH	Ler	CL	Region 2	A-A03	ColLer
0.397580309	CG	Col	CL	Region 1	A-B03	ColLer
0.095880273	CHG	Col	CL	Region 1	A-B03	ColLer
0.019150904	CHH	Col	CL	Region 1	A-B03	ColLer
0.033763654	CG	Ler	CL	Region 1	A-B03	ColLer
0.017935464	CHG	Ler	CL	Region 1	A-B03	ColLer
0.010650806	CHH	Ler	CL	Region 1	A-B03	ColLer
0.905018392	CG	Col	CL	Region 2	A-B03	ColLer
0.533238027	CHG	Col	CL	Region 2	A-B03	ColLer
0.075574721	CHH	Col	CL	Region 2	A-B03	ColLer
0.818110321	CG	Ler	CL	Region 2	A-B03	ColLer
0.441684085	CHG	Ler	CL	Region 2	A-B03	ColLer
0.048214907	CHH	Ler	CL	Region 2	A-B03	ColLer
0.509878419	CG	Col	CL	Region 1	A-C03	ColLer
0.140029873	CHG	Col	CL	Region 1	A-C03	ColLer
0.024105306	CHH	Col	CL	Region 1	A-C03	ColLer
0.021988304	CG	Ler	CL	Region 1	A-C03	ColLer
0.012254676	CHG	Ler	CL	Region 1	A-C03	ColLer
0.008648935	CHH	Ler	CL	Region 1	A-C03	ColLer
0.892270109	CG	Col	CL	Region 2	A-C03	ColLer
0.493520246	CHG	Col	CL	Region 2	A-C03	ColLer
0.065135891	CHH	Col	CL	Region 2	A-C03	ColLer
0.77801563	CG	Ler	CL	Region 2	A-C03	ColLer

0.388276097	CHG	Ler	CL	Region 2	A-C03	ColLer
0.035665181	CHH	Ler	CL	Region 2	A-C03	ColLer
0.542138365	CG	Col	CL	Region 1	A-D03	LerCol
0.18649385	CHG	Col	CL	Region 1	A-D03	LerCol
0.033780346	CHH	Col	CL	Region 1	A-D03	LerCol
0.084781098	CG	Ler	CL	Region 1	A-D03	LerCol
0.042085083	CHG	Ler	CL	Region 1	A-D03	LerCol
0.015976761	CHH	Ler	CL	Region 1	A-D03	LerCol
0.91613399	CG	Col	CL	Region 2	A-D03	LerCol
0.504500818	CHG	Col	CL	Region 2	A-D03	LerCol
0.066137285	CHH	Col	CL	Region 2	A-D03	LerCol
0.755176062	CG	Ler	CL	Region 2	A-D03	LerCol
0.356777897	CHG	Ler	CL	Region 2	A-D03	LerCol
0.034136592	CHH	Ler	CL	Region 2	A-D03	LerCol
0.468820015	CG	Col	CL	Region 1	A-E03	LerCol
0.150257164	CHG	Col	CL	Region 1	A-E03	LerCol
0.029285657	CHH	Col	CL	Region 1	A-E03	LerCol
0.064282011	CG	Ler	CL	Region 1	A-E03	LerCol
0.02767848	CHG	Ler	CL	Region 1	A-E03	LerCol
0.013757853	CHH	Ler	CL	Region 1	A-E03	LerCol
0.893001638	CG	Col	CL	Region 2	A-E03	LerCol
0.514388086	CHG	Col	CL	Region 2	A-E03	LerCol
0.071707516	CHH	Col	CL	Region 2	A-E03	LerCol
0.725795901	CG	Ler	CL	Region 2	A-E03	LerCol
0.343871595	CHG	Ler	CL	Region 2	A-E03	LerCol
0.030768379	CHH	Ler	CL	Region 2	A-E03	LerCol
0.364547782	CG	Col	CL	Region 1	A-F03	LerCol
0.109759168	CHG	Col	CL	Region 1	A-F03	LerCol
0.023551831	CHH	Col	CL	Region 1	A-F03	LerCol
0.017521902	CG	Ler	CL	Region 1	A-F03	LerCol
0.008968979	CHG	Ler	CL	Region 1	A-F03	LerCol
0.008533789	CHH	Ler	CL	Region 1	A-F03	LerCol
0.899785114	CG	Col	CL	Region 2	A-F03	LerCol
0.52912067	CHG	Col	CL	Region 2	A-F03	LerCol
0.082684372	CHH	Col	CL	Region 2	A-F03	LerCol
0.774727196	CG	Ler	CL	Region 2	A-F03	LerCol

0.392583494	CHG	Ler	CL	Region 2	A-F03	LerCol
0.045691336	CHH	Ler	CL	Region 2	A-F03	LerCol
0.384816754	CG	Col	CL	Region 1	A-G03	LerCol
0.100673653	CHG	Col	CL	Region 1	A-G03	LerCol
0.019943137	CHH	Col	CL	Region 1	A-G03	LerCol
0.018204804	CG	Ler	CL	Region 1	A-G03	LerCol
0.009370016	CHG	Ler	CL	Region 1	A-G03	LerCol
0.007613599	CHH	Ler	CL	Region 1	A-G03	LerCol
0.897678809	CG	Col	CL	Region 2	A-G03	LerCol
0.533345033	CHG	Col	CL	Region 2	A-G03	LerCol
0.086604064	CHH	Col	CL	Region 2	A-G03	LerCol
0.707415123	CG	Ler	CL	Region 2	A-G03	LerCol
0.356863464	CHG	Ler	CL	Region 2	A-G03	LerCol
0.03672153	CHH	Ler	CL	Region 2	A-G03	LerCol
0.375626881	CG	Col	CL	Region 1	A-H03	LerCol
0.091733474	CHG	Col	CL	Region 1	A-H03	LerCol
0.01821454	CHH	Col	CL	Region 1	A-H03	LerCol
0.038659015	CG	Ler	CL	Region 1	A-H03	LerCol
0.013230036	CHG	Ler	CL	Region 1	A-H03	LerCol
0.008593647	CHH	Ler	CL	Region 1	A-H03	LerCol
0.905178857	CG	Col	CL	Region 2	A-H03	LerCol
0.523196951	CHG	Col	CL	Region 2	A-H03	LerCol
0.080011688	CHH	Col	CL	Region 2	A-H03	LerCol
0.864686218	CG	Ler	CL	Region 2	A-H03	LerCol
0.459951764	CHG	Ler	CL	Region 2	A-H03	LerCol
0.063594289	CHH	Ler	CL	Region 2	A-H03	LerCol
0.468357674	CG	Col	CL	Region 1	A-A04	LerCol
0.131387598	CHG	Col	CL	Region 1	A-A04	LerCol
0.024437471	CHH	Col	CL	Region 1	A-A04	LerCol
0.032061473	CG	Ler	CL	Region 1	A-A04	LerCol
0.015569488	CHG	Ler	CL	Region 1	A-A04	LerCol
0.010127809	CHH	Ler	CL	Region 1	A-A04	LerCol
0.90639713	CG	Col	CL	Region 2	A-A04	LerCol
0.50526447	CHG	Col	CL	Region 2	A-A04	LerCol
0.070914988	CHH	Col	CL	Region 2	A-A04	LerCol
0.729314735	CG	Ler	CL	Region 2	A-A04	LerCol

0.35468948	CHG	Ler	CL	Region 2	A-A04	LerCol
0.032689313	CHH	Ler	CL	Region 2	A-A04	LerCol
0.53117207	CG	Col	CL	Region 1	A-B04	LerCol
0.139715741	CHG	Col	CL	Region 1	A-B04	LerCol
0.028114664	CHH	Col	CL	Region 1	A-B04	LerCol
0.052570635	CG	Ler	CL	Region 1	A-B04	LerCol
0.020341446	CHG	Ler	CL	Region 1	A-B04	LerCol
0.01154016	CHH	Ler	CL	Region 1	A-B04	LerCol
0.916859609	CG	Col	CL	Region 2	A-B04	LerCol
0.506498068	CHG	Col	CL	Region 2	A-B04	LerCol
0.065119896	CHH	Col	CL	Region 2	A-B04	LerCol
0.735750908	CG	Ler	CL	Region 2	A-B04	LerCol
0.348584964	CHG	Ler	CL	Region 2	A-B04	LerCol
0.02926064	CHH	Ler	CL	Region 2	A-B04	LerCol
0.512702893	CG	Col	CL	Region 1	A-C04	LerCol
0.184484916	CHG	Col	CL	Region 1	A-C04	LerCol
0.037561275	CHH	Col	CL	Region 1	A-C04	LerCol
0.178536585	CG	Ler	CL	Region 1	A-C04	LerCol
0.085323575	CHG	Ler	CL	Region 1	A-C04	LerCol
0.021409722	CHH	Ler	CL	Region 1	A-C04	LerCol
0.884967788	CG	Col	CL	Region 2	A-C04	LerCol
0.507496701	CHG	Col	CL	Region 2	A-C04	LerCol
0.06552371	CHH	Col	CL	Region 2	A-C04	LerCol
0.74849862	CG	Ler	CL	Region 2	A-C04	LerCol
0.381897255	CHG	Ler	CL	Region 2	A-C04	LerCol
0.033450921	CHH	Ler	CL	Region 2	A-C04	LerCol
0.409368635	CG	Col	CL	Region 1	A-D04	LerCol
0.109229382	CHG	Col	CL	Region 1	A-D04	LerCol
0.021930891	CHH	Col	CL	Region 1	A-D04	LerCol
0.015355086	CG	Ler	CL	Region 1	A-D04	LerCol
0.009024495	CHG	Ler	CL	Region 1	A-D04	LerCol
0.008327995	CHH	Ler	CL	Region 1	A-D04	LerCol
0.883893859	CG	Col	CL	Region 2	A-D04	LerCol
0.51441004	CHG	Col	CL	Region 2	A-D04	LerCol
0.074126242	CHH	Col	CL	Region 2	A-D04	LerCol
0.836380781	CG	Ler	CL	Region 2	A-D04	LerCol

0.450277188	CHG	Ler	CL	Region 2	A-D04	LerCol
0.059064633	CHH	Ler	CL	Region 2	A-D04	LerCol
0.485541358	CG	Col	CL	Region 1	A-E04	LerCol
0.168560106	CHG	Col	CL	Region 1	A-E04	LerCol
0.032985605	CHH	Col	CL	Region 1	A-E04	LerCol
0.028248588	CG	Ler	CL	Region 1	A-E04	LerCol
0.010074627	CHG	Ler	CL	Region 1	A-E04	LerCol
0.011139533	CHH	Ler	CL	Region 1	A-E04	LerCol
0.910680576	CG	Col	CL	Region 2	A-E04	LerCol
0.507251209	CHG	Col	CL	Region 2	A-E04	LerCol
0.072321914	CHH	Col	CL	Region 2	A-E04	LerCol
0.864116956	CG	Ler	CL	Region 2	A-E04	LerCol
0.439231944	CHG	Ler	CL	Region 2	A-E04	LerCol
0.050673662	CHH	Ler	CL	Region 2	A-E04	LerCol
0.401160292	CG	Col	CL	Region 1	A-F04	LerCol
0.124524152	CHG	Col	CL	Region 1	A-F04	LerCol
0.025855471	CHH	Col	CL	Region 1	A-F04	LerCol
0.056716418	CG	Ler	CL	Region 1	A-F04	LerCol
0.023126735	CHG	Ler	CL	Region 1	A-F04	LerCol
0.011135485	CHH	Ler	CL	Region 1	A-F04	LerCol
0.884065714	CG	Col	CL	Region 2	A-F04	LerCol
0.505797748	CHG	Col	CL	Region 2	A-F04	LerCol
0.074844075	CHH	Col	CL	Region 2	A-F04	LerCol
0.721419846	CG	Ler	CL	Region 2	A-F04	LerCol
0.35608823	CHG	Ler	CL	Region 2	A-F04	LerCol
0.03914676	CHH	Ler	CL	Region 2	A-F04	LerCol
0.531226673	CG	Col	CL	Region 1	A-G04	LerCol
0.234258166	CHG	Col	CL	Region 1	A-G04	LerCol
0.044655027	CHH	Col	CL	Region 1	A-G04	LerCol
0.049569605	CG	Ler	CL	Region 1	A-G04	LerCol
0.030433769	CHG	Ler	CL	Region 1	A-G04	LerCol
0.014918054	CHH	Ler	CL	Region 1	A-G04	LerCol
0.871965156	CG	Col	CL	Region 2	A-G04	LerCol
0.520627835	CHG	Col	CL	Region 2	A-G04	LerCol
0.087497212	CHH	Col	CL	Region 2	A-G04	LerCol
0.696303607	CG	Ler	CL	Region 2	A-G04	LerCol

0.355266113	CHG	Ler	CL	Region 2	A-G04	LerCol
0.037134953	CHH	Ler	CL	Region 2	A-G04	LerCol
0.446660885	CG	Col	CL	Region 1	A-H04	LerCol
0.090512821	CHG	Col	CL	Region 1	A-H04	LerCol
0.018395311	CHH	Col	CL	Region 1	A-H04	LerCol
0.057173679	CG	Ler	CL	Region 1	A-H04	LerCol
0.02230704	CHG	Ler	CL	Region 1	A-H04	LerCol
0.010190485	CHH	Ler	CL	Region 1	A-H04	LerCol
0.912087467	CG	Col	CL	Region 2	A-H04	LerCol
0.521195321	CHG	Col	CL	Region 2	A-H04	LerCol
0.061380352	CHH	Col	CL	Region 2	A-H04	LerCol
0.816076645	CG	Ler	CL	Region 2	A-H04	LerCol
0.391148924	CHG	Ler	CL	Region 2	A-H04	LerCol
0.032443046	CHH	Ler	CL	Region 2	A-H04	LerCol
0.421558601	CG	Col	CL	Region 1	A-A05	LerCol
0.135582329	CHG	Col	CL	Region 1	A-A05	LerCol
0.030752897	CHH	Col	CL	Region 1	A-A05	LerCol
0.050422139	CG	Ler	CL	Region 1	A-A05	LerCol
0.027004608	CHG	Ler	CL	Region 1	A-A05	LerCol
0.011764945	CHH	Ler	CL	Region 1	A-A05	LerCol
0.875775265	CG	Col	CL	Region 2	A-A05	LerCol
0.528728637	CHG	Col	CL	Region 2	A-A05	LerCol
0.081152098	CHH	Col	CL	Region 2	A-A05	LerCol
0.719960061	CG	Ler	CL	Region 2	A-A05	LerCol
0.360915128	CHG	Ler	CL	Region 2	A-A05	LerCol
0.03570956	CHH	Ler	CL	Region 2	A-A05	LerCol
0.497882772	CG	Col	CL	Region 1	A-B05	LerCol
0.119575699	CHG	Col	CL	Region 1	A-B05	LerCol
0.025333333	CHH	Col	CL	Region 1	A-B05	LerCol
0.057875624	CG	Ler	CL	Region 1	A-B05	LerCol
0.02162452	CHG	Ler	CL	Region 1	A-B05	LerCol
0.009953983	CHH	Ler	CL	Region 1	A-B05	LerCol
0.918325556	CG	Col	CL	Region 2	A-B05	LerCol
0.521629779	CHG	Col	CL	Region 2	A-B05	LerCol
0.068749809	CHH	Col	CL	Region 2	A-B05	LerCol
0.867185081	CG	Ler	CL	Region 2	A-B05	LerCol

0.463655125	CHG	Ler	CL	Region 2	A-B05	LerCol
0.058616843	CHH	Ler	CL	Region 2	A-B05	LerCol
0.426808142	CG	Col	CL	Region 1	A-C05	LerCol
0.096296296	CHG	Col	CL	Region 1	A-C05	LerCol
0.019771306	CHH	Col	CL	Region 1	A-C05	LerCol
0.037604457	CG	Ler	CL	Region 1	A-C05	LerCol
0.017366454	CHG	Ler	CL	Region 1	A-C05	LerCol
0.009022374	CHH	Ler	CL	Region 1	A-C05	LerCol
0.918742957	CG	Col	CL	Region 2	A-C05	LerCol
0.496728948	CHG	Col	CL	Region 2	A-C05	LerCol
0.061386327	CHH	Col	CL	Region 2	A-C05	LerCol
0.823863438	CG	Ler	CL	Region 2	A-C05	LerCol
0.390922827	CHG	Ler	CL	Region 2	A-C05	LerCol
0.034143145	CHH	Ler	CL	Region 2	A-C05	LerCol
0.472548676	CG	Col	CL	Region 1	A-D05	LerCol
0.155789328	CHG	Col	CL	Region 1	A-D05	LerCol
0.028356252	CHH	Col	CL	Region 1	A-D05	LerCol
0.028042843	CG	Ler	CL	Region 1	A-D05	LerCol
0.013678741	CHG	Ler	CL	Region 1	A-D05	LerCol
0.010635604	CHH	Ler	CL	Region 1	A-D05	LerCol
0.913072612	CG	Col	CL	Region 2	A-D05	LerCol
0.531515491	CHG	Col	CL	Region 2	A-D05	LerCol
0.076273225	CHH	Col	CL	Region 2	A-D05	LerCol
0.748679829	CG	Ler	CL	Region 2	A-D05	LerCol
0.36623668	CHG	Ler	CL	Region 2	A-D05	LerCol
0.041266275	CHH	Ler	CL	Region 2	A-D05	LerCol
0.376085241	CG	Col	CL	Region 1	A-E05	LerCol
0.098718447	CHG	Col	CL	Region 1	A-E05	LerCol
0.021961053	CHH	Col	CL	Region 1	A-E05	LerCol
0.036199095	CG	Ler	CL	Region 1	A-E05	LerCol
0.016451479	CHG	Ler	CL	Region 1	A-E05	LerCol
0.009216908	CHH	Ler	CL	Region 1	A-E05	LerCol
0.893233903	CG	Col	CL	Region 2	A-E05	LerCol
0.525235348	CHG	Col	CL	Region 2	A-E05	LerCol
0.07847858	CHH	Col	CL	Region 2	A-E05	LerCol
0.801399756	CG	Ler	CL	Region 2	A-E05	LerCol

0.396126067	CHG	Ler	CL	Region 2	A-E05	LerCol
0.040668397	CHH	Ler	CL	Region 2	A-E05	LerCol
0.515893471	CG	Col	CL	Region 1	A-F05	ColLer2F2
0.170042313	CHG	Col	CL	Region 1	A-F05	ColLer2F2
0.072949801	CHH	Col	CL	Region 1	A-F05	ColLer2F2
0.564642617	CG	Ler	CL	Region 1	A-F05	ColLer2F2
0.287929515	CHG	Ler	CL	Region 1	A-F05	ColLer2F2
0.095228852	CHH	Ler	CL	Region 1	A-F05	ColLer2F2
0.909078074	CG	Ler	LL	Region 2	A-F05	ColLer2F2
0.453756243	CHG	Ler	LL	Region 2	A-F05	ColLer2F2
0.070271711	CHH	Ler	LL	Region 2	A-F05	ColLer2F2
0.151780362	CG	Ler	LL	Region 1	A-G05	ColLer2F2
0.097218943	CHG	Ler	LL	Region 1	A-G05	ColLer2F2
0.084896327	CHH	Ler	LL	Region 1	A-G05	ColLer2F2
0.909069136	CG	Ler	LL	Region 2	A-G05	ColLer2F2
0.44010382	CHG	Ler	LL	Region 2	A-G05	ColLer2F2
0.05783477	CHH	Ler	LL	Region 2	A-G05	ColLer2F2
0.186815774	CG	Ler	LL	Region 1	A-H05	ColLer2F2
0.127552915	CHG	Ler	LL	Region 1	A-H05	ColLer2F2
0.143371301	CHH	Ler	LL	Region 1	A-H05	ColLer2F2
0.92442388	CG	Col	CC	Region 2	A-H05	ColLer2F2
0.507556713	CHG	Col	CC	Region 2	A-H05	ColLer2F2
0.059741175	CHH	Col	CC	Region 2	A-H05	ColLer2F2
0.861605973	CG	Col	CC	Region 1	A-A06	ColLer2F2
0.549253422	CHG	Col	CC	Region 1	A-A06	ColLer2F2
0.382394099	CHH	Col	CC	Region 1	A-A06	ColLer2F2
0.905263918	CG	Ler	LL	Region 2	A-A06	ColLer2F2
0.488966598	CHG	Ler	LL	Region 2	A-A06	ColLer2F2
0.079691869	CHH	Ler	LL	Region 2	A-A06	ColLer2F2
0.012446411	CG	Ler	LL	Region 1	A-B06	ColLer2F2
0.008628693	CHG	Ler	LL	Region 1	A-B06	ColLer2F2
0.00798591	CHH	Ler	LL	Region 1	A-B06	ColLer2F2
0.918408778	CG	Ler	LL	Region 2	A-B06	ColLer2F2
0.479120571	CHG	Ler	LL	Region 2	A-B06	ColLer2F2
0.058244212	CHH	Ler	LL	Region 2	A-B06	ColLer2F2
0.686705989	CG	Col	CL	Region 1	A-C06	ColLer2F2

0.25224336	CHG	Col	CL	Region 1	A-C06	ColLer2F2
0.064871481	CHH	Col	CL	Region 1	A-C06	ColLer2F2
0.623166604	CG	Ler	CL	Region 1	A-C06	ColLer2F2
0.294555192	CHG	Ler	CL	Region 1	A-C06	ColLer2F2
0.068590164	CHH	Ler	CL	Region 1	A-C06	ColLer2F2
0.923616217	CG	Ler	LL	Region 2	A-C06	ColLer2F2
0.453784872	CHG	Ler	LL	Region 2	A-C06	ColLer2F2
0.050647711	CHH	Ler	LL	Region 2	A-C06	ColLer2F2
0.145538201	CG	Ler	LL	Region 1	A-D06	ColLer2F2
0.085480709	CHG	Ler	LL	Region 1	A-D06	ColLer2F2
0.075675505	CHH	Ler	LL	Region 1	A-D06	ColLer2F2
0.938708157	CG	Col	CC	Region 2	A-D06	ColLer2F2
0.528989943	CHG	Col	CC	Region 2	A-D06	ColLer2F2
0.063566573	CHH	Col	CC	Region 2	A-D06	ColLer2F2
0.66629485	CG	Col	CL	Region 1	A-E06	ColLer2F2
0.287778756	CHG	Col	CL	Region 1	A-E06	ColLer2F2
0.116654309	CHH	Col	CL	Region 1	A-E06	ColLer2F2
0.535262206	CG	Ler	CL	Region 1	A-E06	ColLer2F2
0.309092769	CHG	Ler	CL	Region 1	A-E06	ColLer2F2
0.139104424	CHH	Ler	CL	Region 1	A-E06	ColLer2F2
0.90169313	CG	Ler	LL	Region 2	A-E06	ColLer2F2
0.437721939	CHG	Ler	LL	Region 2	A-E06	ColLer2F2
0.059885364	CHH	Ler	LL	Region 2	A-E06	ColLer2F2
0.504892368	CG	Col	CL	Region 1	A-F06	ColLer2F2
0.101916712	CHG	Col	CL	Region 1	A-F06	ColLer2F2
0.023269244	CHH	Col	CL	Region 1	A-F06	ColLer2F2
0.024505184	CG	Ler	CL	Region 1	A-F06	ColLer2F2
0.009981516	CHG	Ler	CL	Region 1	A-F06	ColLer2F2
0.00922901	CHH	Ler	CL	Region 1	A-F06	ColLer2F2
0.916204825	CG	Col	CL	Region 2	A-F06	ColLer2F2
0.512157297	CHG	Col	CL	Region 2	A-F06	ColLer2F2
0.067821882	CHH	Col	CL	Region 2	A-F06	ColLer2F2
0.9209469	CG	Ler	CL	Region 2	A-F06	ColLer2F2
0.489125181	CHG	Ler	CL	Region 2	A-F06	ColLer2F2
0.063877499	CHH	Ler	CL	Region 2	A-F06	ColLer2F2
0.592972402	CG	Col	CL	Region 1	A-G06	ColLer2F2

0.175783797	CHG	Col	CL	Region 1	A-G06	ColLer2F2
0.062096795	CHH	Col	CL	Region 1	A-G06	ColLer2F2
0.59654321	CG	Ler	CL	Region 1	A-G06	ColLer2F2
0.284305108	CHG	Ler	CL	Region 1	A-G06	ColLer2F2
0.084540647	CHH	Ler	CL	Region 1	A-G06	ColLer2F2
0.91851304	CG	Col	CL	Region 2	A-G06	ColLer2F2
0.446526361	CHG	Col	CL	Region 2	A-G06	ColLer2F2
0.054646303	CHH	Col	CL	Region 2	A-G06	ColLer2F2
0.908166624	CG	Ler	CL	Region 2	A-G06	ColLer2F2
0.403925872	CHG	Ler	CL	Region 2	A-G06	ColLer2F2
0.048557682	CHH	Ler	CL	Region 2	A-G06	ColLer2F2
0.032248721	CG	Ler	LL	Region 1	A-H06	ColLer2F2
0.010943081	CHG	Ler	LL	Region 1	A-H06	ColLer2F2
0.008827556	CHH	Ler	LL	Region 1	A-H06	ColLer2F2
0.91215401	CG	Ler	LL	Region 2	A-H06	ColLer2F2
0.444267516	CHG	Ler	LL	Region 2	A-H06	ColLer2F2
0.055891784	CHH	Ler	LL	Region 2	A-H06	ColLer2F2
0.595028409	CG	Col	CL	Region 1	A-A07	ColLer2F2
0.229831039	CHG	Col	CL	Region 1	A-A07	ColLer2F2
0.097721152	CHH	Col	CL	Region 1	A-A07	ColLer2F2
0.641374838	CG	Ler	CL	Region 1	A-A07	ColLer2F2
0.369897959	CHG	Ler	CL	Region 1	A-A07	ColLer2F2
0.135299284	CHH	Ler	CL	Region 1	A-A07	ColLer2F2
0.932950406	CG	Col	CL	Region 2	A-A07	ColLer2F2
0.524450678	CHG	Col	CL	Region 2	A-A07	ColLer2F2
0.076960343	CHH	Col	CL	Region 2	A-A07	ColLer2F2
0.855321615	CG	Ler	CL	Region 2	A-A07	ColLer2F2
0.423773443	CHG	Ler	CL	Region 2	A-A07	ColLer2F2
0.054102073	CHH	Ler	CL	Region 2	A-A07	ColLer2F2
0.670803662	CG	Col	CL	Region 1	A-B07	ColLer2F2
0.294561206	CHG	Col	CL	Region 1	A-B07	ColLer2F2
0.12997109	CHH	Col	CL	Region 1	A-B07	ColLer2F2
0.210581566	CG	Ler	CL	Region 1	A-B07	ColLer2F2
0.133338831	CHG	Ler	CL	Region 1	A-B07	ColLer2F2
0.105654762	CHH	Ler	CL	Region 1	A-B07	ColLer2F2
0.903688965	CG	Col	CL	Region 2	A-B07	ColLer2F2

0.517309506	CHG	Col	CL	Region 2	A-B07	ColLer2F2
0.079895813	CHH	Col	CL	Region 2	A-B07	ColLer2F2
0.89179024	CG	Ler	CL	Region 2	A-B07	ColLer2F2
0.493179595	CHG	Ler	CL	Region 2	A-B07	ColLer2F2
0.075285619	CHH	Ler	CL	Region 2	A-B07	ColLer2F2
0.613735996	CG	Col	CL	Region 1	A-C07	ColLer2F2
0.179509214	CHG	Col	CL	Region 1	A-C07	ColLer2F2
0.027865991	CHH	Col	CL	Region 1	A-C07	ColLer2F2
0.116952156	CG	Ler	CL	Region 1	A-C07	ColLer2F2
0.04128387	CHG	Ler	CL	Region 1	A-C07	ColLer2F2
0.012257799	CHH	Ler	CL	Region 1	A-C07	ColLer2F2
0.912272359	CG	Col	CC	Region 2	A-C07	ColLer2F2
0.513173261	CHG	Col	CC	Region 2	A-C07	ColLer2F2
0.056435736	CHH	Col	CC	Region 2	A-C07	ColLer2F2
0.68627451	CG	Col	CL	Region 1	A-D07	ColLer2F2
0.313147231	CHG	Col	CL	Region 1	A-D07	ColLer2F2
0.096351592	CHH	Col	CL	Region 1	A-D07	ColLer2F2
0.297491039	CG	Ler	CL	Region 1	A-D07	ColLer2F2
0.157399261	CHG	Ler	CL	Region 1	A-D07	ColLer2F2
0.067844605	CHH	Ler	CL	Region 1	A-D07	ColLer2F2
0.922607102	CG	Ler	LL	Region 2	A-D07	ColLer2F2
0.498195051	CHG	Ler	LL	Region 2	A-D07	ColLer2F2
0.075275362	CHH	Ler	LL	Region 2	A-D07	ColLer2F2
0.488391906	CG	Col	CL	Region 1	A-E07	ColLer2F2
0.100435219	CHG	Col	CL	Region 1	A-E07	ColLer2F2
0.020972953	CHH	Col	CL	Region 1	A-E07	ColLer2F2
0.079	CG	Ler	CL	Region 1	A-E07	ColLer2F2
0.016445101	CHG	Ler	CL	Region 1	A-E07	ColLer2F2
0.010368213	CHH	Ler	CL	Region 1	A-E07	ColLer2F2
0.929453914	CG	Ler	LL	Region 2	A-E07	ColLer2F2
0.454885013	CHG	Ler	LL	Region 2	A-E07	ColLer2F2
0.054097735	CHH	Ler	LL	Region 2	A-E07	ColLer2F2
0.858992467	CG	Col	CL	Region 1	A-F07	ColLer2F2
0.56459308	CHG	Col	CL	Region 1	A-F07	ColLer2F2
0.394643002	CHH	Col	CL	Region 1	A-F07	ColLer2F2
0.771828415	CG	Ler	CL	Region 1	A-F07	ColLer2F2

0.557441721	CHG	Ler	CL	Region 1	A-F07	ColLer2F2
0.432773879	CHH	Ler	CL	Region 1	A-F07	ColLer2F2
0.906880577	CG	Ler	LL	Region 2	A-F07	ColLer2F2
0.474948133	CHG	Ler	LL	Region 2	A-F07	ColLer2F2
0.083211865	CHH	Ler	LL	Region 2	A-F07	ColLer2F2
0.723659952	CG	Ler	LL	Region 1	A-G07	ColLer2F2
0.521591031	CHG	Ler	LL	Region 1	A-G07	ColLer2F2
0.439989196	CHH	Ler	LL	Region 1	A-G07	ColLer2F2
0.930111277	CG	Col	CL	Region 2	A-G07	ColLer2F2
0.547718072	CHG	Col	CL	Region 2	A-G07	ColLer2F2
0.089276542	CHH	Col	CL	Region 2	A-G07	ColLer2F2
0.921937639	CG	Ler	CL	Region 2	A-G07	ColLer2F2
0.505449591	CHG	Ler	CL	Region 2	A-G07	ColLer2F2
0.072868618	CHH	Ler	CL	Region 2	A-G07	ColLer2F2
0.708503919	CG	Ler	LL	Region 1	A-H07	ColLer2F2
0.468885606	CHG	Ler	LL	Region 1	A-H07	ColLer2F2
0.219575701	CHH	Ler	LL	Region 1	A-H07	ColLer2F2
0.923014121	CG	Col	CL	Region 2	A-H07	ColLer2F2
0.53636961	CHG	Col	CL	Region 2	A-H07	ColLer2F2
0.073581845	CHH	Col	CL	Region 2	A-H07	ColLer2F2
0.90929368	CG	Ler	CL	Region 2	A-H07	ColLer2F2
0.516450917	CHG	Ler	CL	Region 2	A-H07	ColLer2F2
0.072757938	CHH	Ler	CL	Region 2	A-H07	ColLer2F2
0.115822472	CG	Ler	LL	Region 1	A-A08	ColLer2F2
0.063001813	CHG	Ler	LL	Region 1	A-A08	ColLer2F2
0.060941828	CHH	Ler	LL	Region 1	A-A08	ColLer2F2
0.921371173	CG	Col	CC	Region 2	A-A08	ColLer2F2
0.528718079	CHG	Col	CC	Region 2	A-A08	ColLer2F2
0.066545539	CHH	Col	CC	Region 2	A-A08	ColLer2F2
0.877509173	CG	Col	CC	Region 1	A-B08	ColLer2F2
0.60634663	CHG	Col	CC	Region 1	A-B08	ColLer2F2
0.393035429	CHH	Col	CC	Region 1	A-B08	ColLer2F2
0.929745436	CG	Col	CL	Region 2	A-B08	ColLer2F2
0.556812414	CHG	Col	CL	Region 2	A-B08	ColLer2F2
0.074664418	CHH	Col	CL	Region 2	A-B08	ColLer2F2
0.923456088	CG	Ler	CL	Region 2	A-B08	ColLer2F2

0.531217852	CHG	Ler	CL	Region 2	A-B08	ColLer2F2
0.074265901	CHH	Ler	CL	Region 2	A-B08	ColLer2F2
0.678787879	CG	Col	CL	Region 1	A-C08	ColLer2F2
0.292954709	CHG	Col	CL	Region 1	A-C08	ColLer2F2
0.144787696	CHH	Col	CL	Region 1	A-C08	ColLer2F2
0.159612626	CG	Ler	CL	Region 1	A-C08	ColLer2F2
0.088309859	CHG	Ler	CL	Region 1	A-C08	ColLer2F2
0.100201769	CHH	Ler	CL	Region 1	A-C08	ColLer2F2
0.92268303	CG	Col	CL	Region 2	A-C08	ColLer2F2
0.512610385	CHG	Col	CL	Region 2	A-C08	ColLer2F2
0.077516291	CHH	Col	CL	Region 2	A-C08	ColLer2F2
0.918094724	CG	Ler	CL	Region 2	A-C08	ColLer2F2
0.508621339	CHG	Ler	CL	Region 2	A-C08	ColLer2F2
0.071042587	CHH	Ler	CL	Region 2	A-C08	ColLer2F2
0.766000815	CG	Ler	LL	Region 1	A-D08	ColLer2F2
0.520021471	CHG	Ler	LL	Region 1	A-D08	ColLer2F2
0.355818907	CHH	Ler	LL	Region 1	A-D08	ColLer2F2
0.933556031	CG	Col	CL	Region 2	A-D08	ColLer2F2
0.517418568	CHG	Col	CL	Region 2	A-D08	ColLer2F2
0.079761697	CHH	Col	CL	Region 2	A-D08	ColLer2F2
0.918942475	CG	Ler	CL	Region 2	A-D08	ColLer2F2
0.473388814	CHG	Ler	CL	Region 2	A-D08	ColLer2F2
0.065681329	CHH	Ler	CL	Region 2	A-D08	ColLer2F2
0.761671335	CG	Col	CL	Region 1	A-E08	ColLer2F2
0.40776699	CHG	Col	CL	Region 1	A-E08	ColLer2F2
0.246385334	CHH	Col	CL	Region 1	A-E08	ColLer2F2
0.723842739	CG	Ler	CL	Region 1	A-E08	ColLer2F2
0.449117537	CHG	Ler	CL	Region 1	A-E08	ColLer2F2
0.29842401	CHH	Ler	CL	Region 1	A-E08	ColLer2F2
0.913427706	CG	Ler	LL	Region 2	A-E08	ColLer2F2
0.434458292	CHG	Ler	LL	Region 2	A-E08	ColLer2F2
0.067428855	CHH	Ler	LL	Region 2	A-E08	ColLer2F2
0.902453444	CG	Col	CC	Region 1	A-F08	ColLer2F2
0.593893427	CHG	Col	CC	Region 1	A-F08	ColLer2F2
0.439369744	CHH	Col	CC	Region 1	A-F08	ColLer2F2
0.917433257	CG	Ler	LL	Region 2	A-F08	ColLer2F2

0.485519511	CHG	Ler	LL	Region 2	A-F08	ColLer2F2
0.070578079	CHH	Ler	LL	Region 2	A-F08	ColLer2F2
0.006455399	CG	Ler	LL	Region 1	A-G08	ColLer2F2
0.006859185	CHG	Ler	LL	Region 1	A-G08	ColLer2F2
0.007233457	CHH	Ler	LL	Region 1	A-G08	ColLer2F2
0.929874894	CG	Col	CL	Region 2	A-G08	ColLer2F2
0.506094205	CHG	Col	CL	Region 2	A-G08	ColLer2F2
0.06606225	CHH	Col	CL	Region 2	A-G08	ColLer2F2
0.935624601	CG	Ler	CL	Region 2	A-G08	ColLer2F2
0.486479298	CHG	Ler	CL	Region 2	A-G08	ColLer2F2
0.062806927	CHH	Ler	CL	Region 2	A-G08	ColLer2F2
0.295546079	CG	Ler	LL	Region 1	A-H08	ColLer2F2
0.204343765	CHG	Ler	LL	Region 1	A-H08	ColLer2F2
0.281036583	CHH	Ler	LL	Region 1	A-H08	ColLer2F2
0.924060232	CG	Col	CL	Region 2	A-H08	ColLer2F2
0.526963446	CHG	Col	CL	Region 2	A-H08	ColLer2F2
0.075455903	CHH	Col	CL	Region 2	A-H08	ColLer2F2
0.931654251	CG	Ler	CL	Region 2	A-H08	ColLer2F2
0.520893016	CHG	Ler	CL	Region 2	A-H08	ColLer2F2
0.072748496	CHH	Ler	CL	Region 2	A-H08	ColLer2F2
0.589407115	CG	Col	CL	Region 1	A-A09	ColLer2F2
0.246250545	CHG	Col	CL	Region 1	A-A09	ColLer2F2
0.118093856	CHH	Col	CL	Region 1	A-A09	ColLer2F2
0.590094574	CG	Ler	CL	Region 1	A-A09	ColLer2F2
0.356091819	CHG	Ler	CL	Region 1	A-A09	ColLer2F2
0.153344244	CHH	Ler	CL	Region 1	A-A09	ColLer2F2
0.878234086	CG	Ler	LL	Region 2	A-A09	ColLer2F2
0.471439111	CHG	Ler	LL	Region 2	A-A09	ColLer2F2
0.071424809	CHH	Ler	LL	Region 2	A-A09	ColLer2F2
0.588471742	CG	Col	CC	Region 1	A-B09	ColLer2F2
0.207201777	CHG	Col	CC	Region 1	A-B09	ColLer2F2
0.045021264	CHH	Col	CC	Region 1	A-B09	ColLer2F2
0.927376525	CG	Ler	LL	Region 2	A-B09	ColLer2F2
0.511763955	CHG	Ler	LL	Region 2	A-B09	ColLer2F2
0.07845234	CHH	Ler	LL	Region 2	A-B09	ColLer2F2
0.399228544	CG	Col	CL	Region 1	A-C09	ColLer2F2

0.092938209	CHG	Col	CL	Region 1	A-C09	ColLer2F2
0.036365406	CHH	Col	CL	Region 1	A-C09	ColLer2F2
0.064413265	CG	Ler	CL	Region 1	A-C09	ColLer2F2
0.028230185	CHG	Ler	CL	Region 1	A-C09	ColLer2F2
0.022675277	CHH	Ler	CL	Region 1	A-C09	ColLer2F2
0.908603048	CG	Col	CL	Region 2	A-C09	ColLer2F2
0.463851593	CHG	Col	CL	Region 2	A-C09	ColLer2F2
0.080154798	CHH	Col	CL	Region 2	A-C09	ColLer2F2
0.899872774	CG	Ler	CL	Region 2	A-C09	ColLer2F2
0.44500616	CHG	Ler	CL	Region 2	A-C09	ColLer2F2
0.075456092	CHH	Ler	CL	Region 2	A-C09	ColLer2F2
0.592808117	CG	Col	CL	Region 1	A-D09	ColLer4F2
0.226286386	CHG	Col	CL	Region 1	A-D09	ColLer4F2
0.093372988	CHH	Col	CL	Region 1	A-D09	ColLer4F2
0.320188346	CG	Ler	CL	Region 1	A-D09	ColLer4F2
0.166202198	CHG	Ler	CL	Region 1	A-D09	ColLer4F2
0.080400211	CHH	Ler	CL	Region 1	A-D09	ColLer4F2
0.919262452	CG	Col	CL	Region 2	A-D09	ColLer4F2
0.556903155	CHG	Col	CL	Region 2	A-D09	ColLer4F2
0.078189164	CHH	Col	CL	Region 2	A-D09	ColLer4F2
0.897390743	CG	Ler	CL	Region 2	A-D09	ColLer4F2
0.508100436	CHG	Ler	CL	Region 2	A-D09	ColLer4F2
0.068351635	CHH	Ler	CL	Region 2	A-D09	ColLer4F2
0.780180419	CG	Col	CL	Region 1	A-E09	ColLer4F2
0.364990298	CHG	Col	CL	Region 1	A-E09	ColLer4F2
0.148194601	CHH	Col	CL	Region 1	A-E09	ColLer4F2
0.42962455	CG	Ler	CL	Region 1	A-E09	ColLer4F2
0.305624958	CHG	Ler	CL	Region 1	A-E09	ColLer4F2
0.162484037	CHH	Ler	CL	Region 1	A-E09	ColLer4F2
0.892708276	CG	Col	CC	Region 2	A-E09	ColLer4F2
0.520143835	CHG	Col	CC	Region 2	A-E09	ColLer4F2
0.06210213	CHH	Col	CC	Region 2	A-E09	ColLer4F2
0.619157609	CG	Col	CL	Region 1	A-F09	ColLer4F2
0.21038266	CHG	Col	CL	Region 1	A-F09	ColLer4F2
0.104499394	CHH	Col	CL	Region 1	A-F09	ColLer4F2
0.248761319	CG	Ler	CL	Region 1	A-F09	ColLer4F2

0.114435203	CHG	Ler	CL	Region 1	A-F09	ColLer4F2
0.082686003	CHH	Ler	CL	Region 1	A-F09	ColLer4F2
0.929294824	CG	Col	CL	Region 2	A-F09	ColLer4F2
0.480352273	CHG	Col	CL	Region 2	A-F09	ColLer4F2
0.06609572	CHH	Col	CL	Region 2	A-F09	ColLer4F2
0.900430663	CG	Ler	CL	Region 2	A-F09	ColLer4F2
0.434150077	CHG	Ler	CL	Region 2	A-F09	ColLer4F2
0.055009755	CHH	Ler	CL	Region 2	A-F09	ColLer4F2
0.873175586	CG	Col	CL	Region 1	A-G09	ColLer4F2
0.532776855	CHG	Col	CL	Region 1	A-G09	ColLer4F2
0.408567115	CHH	Col	CL	Region 1	A-G09	ColLer4F2
0.607755054	CG	Ler	CL	Region 1	A-G09	ColLer4F2
0.438909818	CHG	Ler	CL	Region 1	A-G09	ColLer4F2
0.446850955	CHH	Ler	CL	Region 1	A-G09	ColLer4F2
0.870654505	CG	Ler	LL	Region 2	A-G09	ColLer4F2
0.445416499	CHG	Ler	LL	Region 2	A-G09	ColLer4F2
0.060577011	CHH	Ler	LL	Region 2	A-G09	ColLer4F2
0.84781491	CG	Col	CC	Region 1	A-H09	ColLer4F2
0.518479451	CHG	Col	CC	Region 1	A-H09	ColLer4F2
0.382598966	CHH	Col	CC	Region 1	A-H09	ColLer4F2
0.914190138	CG	Col	CL	Region 2	A-H09	ColLer4F2
0.476771399	CHG	Col	CL	Region 2	A-H09	ColLer4F2
0.073243214	CHH	Col	CL	Region 2	A-H09	ColLer4F2
0.863136335	CG	Ler	CL	Region 2	A-H09	ColLer4F2
0.417549084	CHG	Ler	CL	Region 2	A-H09	ColLer4F2
0.065116699	CHH	Ler	CL	Region 2	A-H09	ColLer4F2
0.688741722	CG	Col	CL	Region 1	A-A10	ColLer4F2
0.290542311	CHG	Col	CL	Region 1	A-A10	ColLer4F2
0.158465436	CHH	Col	CL	Region 1	A-A10	ColLer4F2
0.162731707	CG	Ler	CL	Region 1	A-A10	ColLer4F2
0.100531546	CHG	Ler	CL	Region 1	A-A10	ColLer4F2
0.123870444	CHH	Ler	CL	Region 1	A-A10	ColLer4F2
0.914916317	CG	Col	CL	Region 2	A-A10	ColLer4F2
0.479485609	CHG	Col	CL	Region 2	A-A10	ColLer4F2
0.067261441	CHH	Col	CL	Region 2	A-A10	ColLer4F2
0.846613691	CG	Ler	CL	Region 2	A-A10	ColLer4F2

0.403042288	CHG	Ler	CL	Region 2	A-A10	ColLer4F2
0.054057347	CHH	Ler	CL	Region 2	A-A10	ColLer4F2
0.754795278	CG	Col	CC	Region 1	A-B10	ColLer4F2
0.404493973	CHG	Col	CC	Region 1	A-B10	ColLer4F2
0.295517313	CHH	Col	CC	Region 1	A-B10	ColLer4F2
0.917055944	CG	Col	CC	Region 2	A-B10	ColLer4F2
0.518957642	CHG	Col	CC	Region 2	A-B10	ColLer4F2
0.072258886	CHH	Col	CC	Region 2	A-B10	ColLer4F2
0.315019689	CG	Col	CL	Region 1	A-C10	ColLer4F2
0.021546635	CHG	Col	CL	Region 1	A-C10	ColLer4F2
0.01277185	CHH	Col	CL	Region 1	A-C10	ColLer4F2
0.109116809	CG	Ler	CL	Region 1	A-C10	ColLer4F2
0.01676727	CHG	Ler	CL	Region 1	A-C10	ColLer4F2
0.009616811	CHH	Ler	CL	Region 1	A-C10	ColLer4F2
0.922465895	CG	Col	CL	Region 2	A-C10	ColLer4F2
0.470006244	CHG	Col	CL	Region 2	A-C10	ColLer4F2
0.064262501	CHH	Col	CL	Region 2	A-C10	ColLer4F2
0.890100592	CG	Ler	CL	Region 2	A-C10	ColLer4F2
0.418270491	CHG	Ler	CL	Region 2	A-C10	ColLer4F2
0.054864199	CHH	Ler	CL	Region 2	A-C10	ColLer4F2
0.910703364	CG	Col	CC	Region 1	A-D10	ColLer4F2
0.624840699	CHG	Col	CC	Region 1	A-D10	ColLer4F2
0.350516085	CHH	Col	CC	Region 1	A-D10	ColLer4F2
0.895003571	CG	Ler	LL	Region 2	A-D10	ColLer4F2
0.489205772	CHG	Ler	LL	Region 2	A-D10	ColLer4F2
0.055588287	CHH	Ler	LL	Region 2	A-D10	ColLer4F2
0.190753425	CG	Col	CL	Region 1	A-E10	ColLer4F2
0.076612632	CHG	Col	CL	Region 1	A-E10	ColLer4F2
0.039915748	CHH	Col	CL	Region 1	A-E10	ColLer4F2
0.074159021	CG	Ler	CL	Region 1	A-E10	ColLer4F2
0.042663996	CHG	Ler	CL	Region 1	A-E10	ColLer4F2
0.031413034	CHH	Ler	CL	Region 1	A-E10	ColLer4F2
0.911611731	CG	Col	CC	Region 2	A-E10	ColLer4F2
0.466791394	CHG	Col	CC	Region 2	A-E10	ColLer4F2
0.065582523	CHH	Col	CC	Region 2	A-E10	ColLer4F2
0.020310766	CG	Ler	LL	Region 1	A-F10	ColLer4F2

0.009684063	CHG	Ler	LL	Region 1	A-F10	ColLer4F2
0.008149936	CHH	Ler	LL	Region 1	A-F10	ColLer4F2
0.931970811	CG	Col	CL	Region 2	A-F10	ColLer4F2
0.493735431	CHG	Col	CL	Region 2	A-F10	ColLer4F2
0.066597184	CHH	Col	CL	Region 2	A-F10	ColLer4F2
0.852630559	CG	Ler	CL	Region 2	A-F10	ColLer4F2
0.415642518	CHG	Ler	CL	Region 2	A-F10	ColLer4F2
0.051016001	CHH	Ler	CL	Region 2	A-F10	ColLer4F2
0.40199268	CG	Col	CL	Region 1	A-G10	ColLer4F2
0.259345607	CHG	Col	CL	Region 1	A-G10	ColLer4F2
0.193548956	CHH	Col	CL	Region 1	A-G10	ColLer4F2
0.229724902	CG	Ler	CL	Region 1	A-G10	ColLer4F2
0.146841327	CHG	Ler	CL	Region 1	A-G10	ColLer4F2
0.183348301	CHH	Ler	CL	Region 1	A-G10	ColLer4F2
0.940831249	CG	Col	CL	Region 2	A-G10	ColLer4F2
0.529806299	CHG	Col	CL	Region 2	A-G10	ColLer4F2
0.083871031	CHH	Col	CL	Region 2	A-G10	ColLer4F2
0.899844972	CG	Ler	CL	Region 2	A-G10	ColLer4F2
0.489958386	CHG	Ler	CL	Region 2	A-G10	ColLer4F2
0.073376182	CHH	Ler	CL	Region 2	A-G10	ColLer4F2
0.829170384	CG	Col	CL	Region 1	A-H10	ColLer4F2
0.470758693	CHG	Col	CL	Region 1	A-H10	ColLer4F2
0.255889599	CHH	Col	CL	Region 1	A-H10	ColLer4F2
0.456210647	CG	Ler	CL	Region 1	A-H10	ColLer4F2
0.328846154	CHG	Ler	CL	Region 1	A-H10	ColLer4F2
0.263178785	CHH	Ler	CL	Region 1	A-H10	ColLer4F2
0.929766912	CG	Col	CL	Region 2	A-H10	ColLer4F2
0.552728073	CHG	Col	CL	Region 2	A-H10	ColLer4F2
0.069477158	CHH	Col	CL	Region 2	A-H10	ColLer4F2
0.883438744	CG	Ler	CL	Region 2	A-H10	ColLer4F2
0.499490556	CHG	Ler	CL	Region 2	A-H10	ColLer4F2
0.056914856	CHH	Ler	CL	Region 2	A-H10	ColLer4F2
0.064527758	CG	Ler	LL	Region 1	A-A11	ColLer4F2
0.028958806	CHG	Ler	LL	Region 1	A-A11	ColLer4F2
0.024980964	CHH	Ler	LL	Region 1	A-A11	ColLer4F2
0.908559511	CG	Col	CL	Region 2	A-A11	ColLer4F2

0.433179724	CHG	Col	CL	Region 2	A-A11	ColLer4F2
0.059655037	CHH	Col	CL	Region 2	A-A11	ColLer4F2
0.861479782	CG	Ler	CL	Region 2	A-A11	ColLer4F2
0.395431761	CHG	Ler	CL	Region 2	A-A11	ColLer4F2
0.053512933	CHH	Ler	CL	Region 2	A-A11	ColLer4F2
0.234827382	CG	Ler	LL	Region 1	A-B11	ColLer4F2
0.165248604	CHG	Ler	LL	Region 1	A-B11	ColLer4F2
0.262344125	CHH	Ler	LL	Region 1	A-B11	ColLer4F2
0.901511668	CG	Col	CL	Region 2	A-B11	ColLer4F2
0.492684097	CHG	Col	CL	Region 2	A-B11	ColLer4F2
0.073575419	CHH	Col	CL	Region 2	A-B11	ColLer4F2
0.839048826	CG	Ler	CL	Region 2	A-B11	ColLer4F2
0.427190488	CHG	Ler	CL	Region 2	A-B11	ColLer4F2
0.058736143	CHH	Ler	CL	Region 2	A-B11	ColLer4F2
0.324185249	CG	Col	CL	Region 1	A-C11	ColLer4F2
0.055353979	CHG	Col	CL	Region 1	A-C11	ColLer4F2
0.011998731	CHH	Col	CL	Region 1	A-C11	ColLer4F2
0.039232781	CG	Ler	CL	Region 1	A-C11	ColLer4F2
0.010994674	CHG	Ler	CL	Region 1	A-C11	ColLer4F2
0.008443151	CHH	Ler	CL	Region 1	A-C11	ColLer4F2
0.940444302	CG	Col	CC	Region 2	A-C11	ColLer4F2
0.55013992	CHG	Col	CC	Region 2	A-C11	ColLer4F2
0.081908816	CHH	Col	CC	Region 2	A-C11	ColLer4F2
0.560734463	CG	Col	CL	Region 1	A-D11	ColLer4F2
0.190639481	CHG	Col	CL	Region 1	A-D11	ColLer4F2
0.060286652	CHH	Col	CL	Region 1	A-D11	ColLer4F2
0.122789288	CG	Ler	CL	Region 1	A-D11	ColLer4F2
0.054975644	CHG	Ler	CL	Region 1	A-D11	ColLer4F2
0.034603063	CHH	Ler	CL	Region 1	A-D11	ColLer4F2
0.924147202	CG	Col	CL	Region 2	A-D11	ColLer4F2
0.524973313	CHG	Col	CL	Region 2	A-D11	ColLer4F2
0.077121688	CHH	Col	CL	Region 2	A-D11	ColLer4F2
0.894405695	CG	Ler	CL	Region 2	A-D11	ColLer4F2
0.465251544	CHG	Ler	CL	Region 2	A-D11	ColLer4F2
0.061226936	CHH	Ler	CL	Region 2	A-D11	ColLer4F2
0.511234797	CG	Col	CL	Region 1	A-E11	ColLer4F2

0.077223533	CHG	Col	CL	Region 1	A-E11	ColLer4F2
0.019877292	CHH	Col	CL	Region 1	A-E11	ColLer4F2
0.033625385	CG	Ler	CL	Region 1	A-E11	ColLer4F2
0.010935601	CHG	Ler	CL	Region 1	A-E11	ColLer4F2
0.007934212	CHH	Ler	CL	Region 1	A-E11	ColLer4F2
0.937415649	CG	Col	CL	Region 2	A-E11	ColLer4F2
0.506089878	CHG	Col	CL	Region 2	A-E11	ColLer4F2
0.072418208	CHH	Col	CL	Region 2	A-E11	ColLer4F2
0.852434128	CG	Ler	CL	Region 2	A-E11	ColLer4F2
0.416356248	CHG	Ler	CL	Region 2	A-E11	ColLer4F2
0.054521714	CHH	Ler	CL	Region 2	A-E11	ColLer4F2
0.415519126	CG	Col	CL	Region 1	A-F11	ColLer4F2
0.068381972	CHG	Col	CL	Region 1	A-F11	ColLer4F2
0.016213639	CHH	Col	CL	Region 1	A-F11	ColLer4F2
0.057490212	CG	Ler	CL	Region 1	A-F11	ColLer4F2
0.011235955	CHG	Ler	CL	Region 1	A-F11	ColLer4F2
0.008370988	CHH	Ler	CL	Region 1	A-F11	ColLer4F2
0.918039216	CG	Col	CL	Region 2	A-F11	ColLer4F2
0.487718149	CHG	Col	CL	Region 2	A-F11	ColLer4F2
0.058910087	CHH	Col	CL	Region 2	A-F11	ColLer4F2
0.837777778	CG	Ler	CL	Region 2	A-F11	ColLer4F2
0.427743374	CHG	Ler	CL	Region 2	A-F11	ColLer4F2
0.048931647	CHH	Ler	CL	Region 2	A-F11	ColLer4F2
0.675319149	CG	Col	CC	Region 1	A-G11	ColLer4F2
0.278757939	CHG	Col	CC	Region 1	A-G11	ColLer4F2
0.08514004	CHH	Col	CC	Region 1	A-G11	ColLer4F2
0.902490451	CG	Col	CL	Region 2	A-G11	ColLer4F2
0.546271706	CHG	Col	CL	Region 2	A-G11	ColLer4F2
0.077602506	CHH	Col	CL	Region 2	A-G11	ColLer4F2
0.851957248	CG	Ler	CL	Region 2	A-G11	ColLer4F2
0.48682102	CHG	Ler	CL	Region 2	A-G11	ColLer4F2
0.0601627	CHH	Ler	CL	Region 2	A-G11	ColLer4F2
0.850322008	CG	Col	CC	Region 1	A-H11	ColLer4F2
0.498299519	CHG	Col	CC	Region 1	A-H11	ColLer4F2
0.384138046	CHH	Col	CC	Region 1	A-H11	ColLer4F2
0.911376066	CG	Col	CL	Region 2	A-H11	ColLer4F2

0.522231662	CHG	Col	CL	Region 2	A-H11	ColLer4F2
0.085398768	CHH	Col	CL	Region 2	A-H11	ColLer4F2
0.838134384	CG	Ler	CL	Region 2	A-H11	ColLer4F2
0.445877607	CHG	Ler	CL	Region 2	A-H11	ColLer4F2
0.062672887	CHH	Ler	CL	Region 2	A-H11	ColLer4F2
0.053251183	CG	Ler	LL	Region 1	A-A12	ColLer4F2
0.01990785	CHG	Ler	LL	Region 1	A-A12	ColLer4F2
0.016699014	CHH	Ler	LL	Region 1	A-A12	ColLer4F2
0.909787484	CG	Col	CC	Region 2	A-A12	ColLer4F2
0.480731917	CHG	Col	CC	Region 2	A-A12	ColLer4F2
0.067802059	CHH	Col	CC	Region 2	A-A12	ColLer4F2
0.612997526	CG	Col	CL	Region 1	A-B12	ColLer4F2
0.242273535	CHG	Col	CL	Region 1	A-B12	ColLer4F2
0.121178378	CHH	Col	CL	Region 1	A-B12	ColLer4F2
0.201899795	CG	Ler	CL	Region 1	A-B12	ColLer4F2
0.10301121	CHG	Ler	CL	Region 1	A-B12	ColLer4F2
0.080765813	CHH	Ler	CL	Region 1	A-B12	ColLer4F2
0.905866906	CG	Col	CC	Region 2	A-B12	ColLer4F2
0.536936776	CHG	Col	CC	Region 2	A-B12	ColLer4F2
0.07178377	CHH	Col	CC	Region 2	A-B12	ColLer4F2
0.843783209	CG	Col	CL	Region 1	A-C12	ColLer4F2
0.513451974	CHG	Col	CL	Region 1	A-C12	ColLer4F2
0.285986241	CHH	Col	CL	Region 1	A-C12	ColLer4F2
0.384103812	CG	Ler	CL	Region 1	A-C12	ColLer4F2
0.291474654	CHG	Ler	CL	Region 1	A-C12	ColLer4F2
0.276705711	CHH	Ler	CL	Region 1	A-C12	ColLer4F2
0.933088084	CG	Col	CL	Region 2	A-C12	ColLer4F2
0.556734238	CHG	Col	CL	Region 2	A-C12	ColLer4F2
0.085314685	CHH	Col	CL	Region 2	A-C12	ColLer4F2
0.880042139	CG	Ler	CL	Region 2	A-C12	ColLer4F2
0.470034762	CHG	Ler	CL	Region 2	A-C12	ColLer4F2
0.061105534	CHH	Ler	CL	Region 2	A-C12	ColLer4F2
0.140244613	CG	Ler	LL	Region 1	A-D12	ColLer4F2
0.078812081	CHG	Ler	LL	Region 1	A-D12	ColLer4F2
0.058705459	CHH	Ler	LL	Region 1	A-D12	ColLer4F2
0.917734265	CG	Col	CC	Region 2	A-D12	ColLer4F2

0.466972017	CHG	Col	CC	Region 2	A-D12	ColLer4F2
0.054833166	CHH	Col	CC	Region 2	A-D12	ColLer4F2
0.836111442	CG	Col	CC	Region 1	A-E12	ColLer4F2
0.529538685	CHG	Col	CC	Region 1	A-E12	ColLer4F2
0.369367935	CHH	Col	CC	Region 1	A-E12	ColLer4F2
0.925917132	CG	Col	CL	Region 2	A-E12	ColLer4F2
0.516105055	CHG	Col	CL	Region 2	A-E12	ColLer4F2
0.075566773	CHH	Col	CL	Region 2	A-E12	ColLer4F2
0.881473148	CG	Ler	CL	Region 2	A-E12	ColLer4F2
0.463732862	CHG	Ler	CL	Region 2	A-E12	ColLer4F2
0.062922978	CHH	Ler	CL	Region 2	A-E12	ColLer4F2
0.667995651	CG	Col	CL	Region 1	A-F12	ColLer4F2
0.283473028	CHG	Col	CL	Region 1	A-F12	ColLer4F2
0.164160302	CHH	Col	CL	Region 1	A-F12	ColLer4F2
0.164830386	CG	Ler	CL	Region 1	A-F12	ColLer4F2
0.096413068	CHG	Ler	CL	Region 1	A-F12	ColLer4F2
0.114631815	CHH	Ler	CL	Region 1	A-F12	ColLer4F2
0.90803143	CG	Col	CC	Region 2	A-F12	ColLer4F2
0.456635572	CHG	Col	CC	Region 2	A-F12	ColLer4F2
0.068242551	CHH	Col	CC	Region 2	A-F12	ColLer4F2
0.528802805	CG	Col	CL	Region 1	A-G12	ColLer4F2
0.147573326	CHG	Col	CL	Region 1	A-G12	ColLer4F2
0.05428302	CHH	Col	CL	Region 1	A-G12	ColLer4F2
0.149370232	CG	Ler	CL	Region 1	A-G12	ColLer4F2
0.066414989	CHG	Ler	CL	Region 1	A-G12	ColLer4F2
0.038970259	CHH	Ler	CL	Region 1	A-G12	ColLer4F2
0.906138477	CG	Col	CC	Region 2	A-G12	ColLer4F2
0.507390909	CHG	Col	CC	Region 2	A-G12	ColLer4F2
0.061673316	CHH	Col	CC	Region 2	A-G12	ColLer4F2
0.766276907	CG	Col	CC	Region 1	B-A01	LerCol3F2
0.404818139	CHG	Col	CC	Region 1	B-A01	LerCol3F2
0.238679479	CHH	Col	CC	Region 1	B-A01	LerCol3F2
0.910792439	CG	Col	CL	Region 2	B-A01	LerCol3F2
0.491041839	CHG	Col	CL	Region 2	B-A01	LerCol3F2
0.056488779	CHH	Col	CL	Region 2	B-A01	LerCol3F2
0.912327124	CG	Ler	CL	Region 2	B-A01	LerCol3F2

0.471462264	CHG	Ler	CL	Region 2	B-A01	LerCol3F2
0.05556747	CHH	Ler	CL	Region 2	B-A01	LerCol3F2
0.646757054	CG	Col	CL	Region 1	B-B01	LerCol3F2
0.222230237	CHG	Col	CL	Region 1	B-B01	LerCol3F2
0.038924015	CHH	Col	CL	Region 1	B-B01	LerCol3F2
0.146250665	CG	Ler	CL	Region 1	B-B01	LerCol3F2
0.061744779	CHG	Ler	CL	Region 1	B-B01	LerCol3F2
0.026504983	CHH	Ler	CL	Region 1	B-B01	LerCol3F2
0.903710849	CG	Col	CC	Region 2	B-B01	LerCol3F2
0.504945809	CHG	Col	CC	Region 2	B-B01	LerCol3F2
0.05900815	CHH	Col	CC	Region 2	B-B01	LerCol3F2
0.524803388	CG	Col	CL	Region 1	B-C01	LerCol3F2
0.130393325	CHG	Col	CL	Region 1	B-C01	LerCol3F2
0.020342725	CHH	Col	CL	Region 1	B-C01	LerCol3F2
0.317046208	CG	Ler	CL	Region 1	B-C01	LerCol3F2
0.111169057	CHG	Ler	CL	Region 1	B-C01	LerCol3F2
0.014895863	CHH	Ler	CL	Region 1	B-C01	LerCol3F2
0.928870902	CG	Ler	LL	Region 2	B-C01	LerCol3F2
0.510167348	CHG	Ler	LL	Region 2	B-C01	LerCol3F2
0.05700574	CHH	Ler	LL	Region 2	B-C01	LerCol3F2
0.65078329	CG	Col	CL	Region 1	B-D01	LerCol3F2
0.235127843	CHG	Col	CL	Region 1	B-D01	LerCol3F2
0.076661712	CHH	Col	CL	Region 1	B-D01	LerCol3F2
0.133511859	CG	Ler	CL	Region 1	B-D01	LerCol3F2
0.068592871	CHG	Ler	CL	Region 1	B-D01	LerCol3F2
0.048936346	CHH	Ler	CL	Region 1	B-D01	LerCol3F2
0.930600119	CG	Col	CC	Region 2	B-D01	LerCol3F2
0.513101087	CHG	Col	CC	Region 2	B-D01	LerCol3F2
0.068673731	CHH	Col	CC	Region 2	B-D01	LerCol3F2
0.522450239	CG	Col	CL	Region 1	B-E01	LerCol3F2
0.200255412	CHG	Col	CL	Region 1	B-E01	LerCol3F2
0.091058871	CHH	Col	CL	Region 1	B-E01	LerCol3F2
0.465578111	CG	Ler	CL	Region 1	B-E01	LerCol3F2
0.244214373	CHG	Ler	CL	Region 1	B-E01	LerCol3F2
0.108853578	CHH	Ler	CL	Region 1	B-E01	LerCol3F2
0.920193152	CG	Col	CL	Region 2	B-E01	LerCol3F2

0.500024425	CHG	Col	CL	Region 2	B-E01	LerCol3F2
0.076832834	CHH	Col	CL	Region 2	B-E01	LerCol3F2
0.918764978	CG	Ler	CL	Region 2	B-E01	LerCol3F2
0.480060283	CHG	Ler	CL	Region 2	B-E01	LerCol3F2
0.074219451	CHH	Ler	CL	Region 2	B-E01	LerCol3F2
0.858397366	CG	Col	CC	Region 1	B-F01	LerCol3F2
0.501646206	CHG	Col	CC	Region 1	B-F01	LerCol3F2
0.333523362	CHH	Col	CC	Region 1	B-F01	LerCol3F2
0.935523439	CG	Col	CL	Region 2	B-F01	LerCol3F2
0.526037501	CHG	Col	CL	Region 2	B-F01	LerCol3F2
0.070208207	CHH	Col	CL	Region 2	B-F01	LerCol3F2
0.927232513	CG	Ler	CL	Region 2	B-F01	LerCol3F2
0.497206962	CHG	Ler	CL	Region 2	B-F01	LerCol3F2
0.064387517	CHH	Ler	CL	Region 2	B-F01	LerCol3F2
0.797915891	CG	Col	CC	Region 1	B-G01	LerCol3F2
0.449583909	CHG	Col	CC	Region 1	B-G01	LerCol3F2
0.250215831	CHH	Col	CC	Region 1	B-G01	LerCol3F2
0.903319716	CG	Col	CC	Region 2	B-G01	LerCol3F2
0.508177448	CHG	Col	CC	Region 2	B-G01	LerCol3F2
0.069077917	CHH	Col	CC	Region 2	B-G01	LerCol3F2
0.518961739	CG	Col	CL	Region 1	B-H01	LerCol3F2
0.145943796	CHG	Col	CL	Region 1	B-H01	LerCol3F2
0.033262665	CHH	Col	CL	Region 1	B-H01	LerCol3F2
0.127837707	CG	Ler	CL	Region 1	B-H01	LerCol3F2
0.039161016	CHG	Ler	CL	Region 1	B-H01	LerCol3F2
0.016594254	CHH	Ler	CL	Region 1	B-H01	LerCol3F2
0.925824085	CG	Ler	LL	Region 2	B-H01	LerCol3F2
0.487519565	CHG	Ler	LL	Region 2	B-H01	LerCol3F2
0.066140459	CHH	Ler	LL	Region 2	B-H01	LerCol3F2
0.066331788	CG	Ler	LL	Region 1	B-A02	LerCol3F2
0.022750304	CHG	Ler	LL	Region 1	B-A02	LerCol3F2
0.011616444	CHH	Ler	LL	Region 1	B-A02	LerCol3F2
0.928992477	CG	Col	CL	Region 2	B-A02	LerCol3F2
0.525137496	CHG	Col	CL	Region 2	B-A02	LerCol3F2
0.059043938	CHH	Col	CL	Region 2	B-A02	LerCol3F2
0.924055247	CG	Ler	CL	Region 2	B-A02	LerCol3F2

0.521331173	CHG	Ler	CL	Region 2	B-A02	LerCol3F2
0.059017361	CHH	Ler	CL	Region 2	B-A02	LerCol3F2
0.443888194	CG	Col	CL	Region 1	B-B02	LerCol3F2
0.117801909	CHG	Col	CL	Region 1	B-B02	LerCol3F2
0.042259141	CHH	Col	CL	Region 1	B-B02	LerCol3F2
0.070874862	CG	Ler	CL	Region 1	B-B02	LerCol3F2
0.027850305	CHG	Ler	CL	Region 1	B-B02	LerCol3F2
0.02241937	CHH	Ler	CL	Region 1	B-B02	LerCol3F2
0.898586113	CG	Col	CL	Region 2	B-B02	LerCol3F2
0.463620201	CHG	Col	CL	Region 2	B-B02	LerCol3F2
0.071903634	CHH	Col	CL	Region 2	B-B02	LerCol3F2
0.889693356	CG	Ler	CL	Region 2	B-B02	LerCol3F2
0.439235741	CHG	Ler	CL	Region 2	B-B02	LerCol3F2
0.063807748	CHH	Ler	CL	Region 2	B-B02	LerCol3F2
0.792580703	CG	Col	CL	Region 1	B-C02	LerCol3F2
0.378325123	CHG	Col	CL	Region 1	B-C02	LerCol3F2
0.294502376	CHH	Col	CL	Region 1	B-C02	LerCol3F2
0.634413039	CG	Ler	CL	Region 1	B-C02	LerCol3F2
0.375203252	CHG	Ler	CL	Region 1	B-C02	LerCol3F2
0.344026805	CHH	Ler	CL	Region 1	B-C02	LerCol3F2
0.914585635	CG	Col	CL	Region 2	B-C02	LerCol3F2
0.440891235	CHG	Col	CL	Region 2	B-C02	LerCol3F2
0.065763052	CHH	Col	CL	Region 2	B-C02	LerCol3F2
0.907258396	CG	Ler	CL	Region 2	B-C02	LerCol3F2
0.424878616	CHG	Ler	CL	Region 2	B-C02	LerCol3F2
0.061113745	CHH	Ler	CL	Region 2	B-C02	LerCol3F2
0.786109928	CG	Col	CL	Region 1	B-D02	LerCol3F2
0.427177935	CHG	Col	CL	Region 1	B-D02	LerCol3F2
0.23381295	CHH	Col	CL	Region 1	B-D02	LerCol3F2
0.498910675	CG	Ler	CL	Region 1	B-D02	LerCol3F2
0.30772164	CHG	Ler	CL	Region 1	B-D02	LerCol3F2
0.231094548	CHH	Ler	CL	Region 1	B-D02	LerCol3F2
0.917690556	CG	Ler	LL	Region 2	B-D02	LerCol3F2
0.488585505	CHG	Ler	LL	Region 2	B-D02	LerCol3F2
0.071717974	CHH	Ler	LL	Region 2	B-D02	LerCol3F2
0.61259542	CG	Ler	LL	Region 1	B-E02	LerCol3F2

0.322112753	CHG	Ler	LL	Region 1	B-E02	LerCol3F2
0.090253414	CHH	Ler	LL	Region 1	B-E02	LerCol3F2
0.910513938	CG	Col	CL	Region 2	B-E02	LerCol3F2
0.457973999	CHG	Col	CL	Region 2	B-E02	LerCol3F2
0.057115436	CHH	Col	CL	Region 2	B-E02	LerCol3F2
0.912173359	CG	Ler	CL	Region 2	B-E02	LerCol3F2
0.425136535	CHG	Ler	CL	Region 2	B-E02	LerCol3F2
0.048168538	CHH	Ler	CL	Region 2	B-E02	LerCol3F2
0.48961566	CG	Col	CL	Region 1	B-F02	LerCol3F2
0.106231898	CHG	Col	CL	Region 1	B-F02	LerCol3F2
0.021100521	CHH	Col	CL	Region 1	B-F02	LerCol3F2
0.253820961	CG	Ler	CL	Region 1	B-F02	LerCol3F2
0.052987962	CHG	Ler	CL	Region 1	B-F02	LerCol3F2
0.013427914	CHH	Ler	CL	Region 1	B-F02	LerCol3F2
0.924701733	CG	Col	CL	Region 2	B-F02	LerCol3F2
0.502976004	CHG	Col	CL	Region 2	B-F02	LerCol3F2
0.063749872	CHH	Col	CL	Region 2	B-F02	LerCol3F2
0.924053546	CG	Ler	CL	Region 2	B-F02	LerCol3F2
0.487399258	CHG	Ler	CL	Region 2	B-F02	LerCol3F2
0.058659933	CHH	Ler	CL	Region 2	B-F02	LerCol3F2
0.717874675	CG	Ler	LL	Region 1	B-G02	LerCol3F2
0.532735375	CHG	Ler	LL	Region 1	B-G02	LerCol3F2
0.481476384	CHH	Ler	LL	Region 1	B-G02	LerCol3F2
0.927511158	CG	Col	CC	Region 2	B-G02	LerCol3F2
0.569418858	CHG	Col	CC	Region 2	B-G02	LerCol3F2
0.079682366	CHH	Col	CC	Region 2	B-G02	LerCol3F2
0.269688529	CG	Ler	LL	Region 1	B-H02	LerCol3F2
0.195490982	CHG	Ler	LL	Region 1	B-H02	LerCol3F2
0.27879785	CHH	Ler	LL	Region 1	B-H02	LerCol3F2
0.922282806	CG	Col	CL	Region 2	B-H02	LerCol3F2
0.49756454	CHG	Col	CL	Region 2	B-H02	LerCol3F2
0.077901114	CHH	Col	CL	Region 2	B-H02	LerCol3F2
0.913629595	CG	Ler	CL	Region 2	B-H02	LerCol3F2
0.478368841	CHG	Ler	CL	Region 2	B-H02	LerCol3F2
0.063550558	CHH	Ler	CL	Region 2	B-H02	LerCol3F2
0.765201686	CG	Col	CL	Region 1	B-A03	LerCol3F2

0.415535983	CHG	Col	CL	Region 1	B-A03	LerCol3F2
0.219819788	CHH	Col	CL	Region 1	B-A03	LerCol3F2
0.185822785	CG	Ler	CL	Region 1	B-A03	LerCol3F2
0.129514073	CHG	Ler	CL	Region 1	B-A03	LerCol3F2
0.131320379	CHH	Ler	CL	Region 1	B-A03	LerCol3F2
0.929464241	CG	Ler	LL	Region 2	B-A03	LerCol3F2
0.536393771	CHG	Ler	LL	Region 2	B-A03	LerCol3F2
0.081163247	CHH	Ler	LL	Region 2	B-A03	LerCol3F2
0.849424712	CG	Col	CL	Region 1	B-B03	LerCol3F2
0.534616148	CHG	Col	CL	Region 1	B-B03	LerCol3F2
0.313855631	CHH	Col	CL	Region 1	B-B03	LerCol3F2
0.328836425	CG	Ler	CL	Region 1	B-B03	LerCol3F2
0.262736374	CHG	Ler	CL	Region 1	B-B03	LerCol3F2
0.281699761	CHH	Ler	CL	Region 1	B-B03	LerCol3F2
0.928588471	CG	Ler	LL	Region 2	B-B03	LerCol3F2
0.531300801	CHG	Ler	LL	Region 2	B-B03	LerCol3F2
0.083050923	CHH	Ler	LL	Region 2	B-B03	LerCol3F2
0.776780809	CG	Col	CL	Region 1	B-C03	LerCol3F2
0.443962087	CHG	Col	CL	Region 1	B-C03	LerCol3F2
0.256671626	CHH	Col	CL	Region 1	B-C03	LerCol3F2
0.305823017	CG	Ler	CL	Region 1	B-C03	LerCol3F2
0.218594555	CHG	Ler	CL	Region 1	B-C03	LerCol3F2
0.240054788	CHH	Ler	CL	Region 1	B-C03	LerCol3F2
0.92103478	CG	Col	CL	Region 2	B-C03	LerCol3F2
0.526378095	CHG	Col	CL	Region 2	B-C03	LerCol3F2
0.074585558	CHH	Col	CL	Region 2	B-C03	LerCol3F2
0.908676759	CG	Ler	CL	Region 2	B-C03	LerCol3F2
0.49809641	CHG	Ler	CL	Region 2	B-C03	LerCol3F2
0.071859093	CHH	Ler	CL	Region 2	B-C03	LerCol3F2
0.675303066	CG	Col	CL	Region 1	B-D03	LerCol3F2
0.30382708	CHG	Col	CL	Region 1	B-D03	LerCol3F2
0.091617813	CHH	Col	CL	Region 1	B-D03	LerCol3F2
0.171792153	CG	Ler	CL	Region 1	B-D03	LerCol3F2
0.112405116	CHG	Ler	CL	Region 1	B-D03	LerCol3F2
0.068680627	CHH	Ler	CL	Region 1	B-D03	LerCol3F2
0.934962499	CG	Col	CL	Region 2	B-D03	LerCol3F2

0.547818396	CHG	Col	CL	Region 2	B-D03	LerCol3F2
0.072775329	CHH	Col	CL	Region 2	B-D03	LerCol3F2
0.933140097	CG	Ler	CL	Region 2	B-D03	LerCol3F2
0.525614489	CHG	Ler	CL	Region 2	B-D03	LerCol3F2
0.066961874	CHH	Ler	CL	Region 2	B-D03	LerCol3F2
0.808507249	CG	Col	CC	Region 1	B-E03	LerCol3F2
0.431568056	CHG	Col	CC	Region 1	B-E03	LerCol3F2
0.282656908	CHH	Col	CC	Region 1	B-E03	LerCol3F2
0.91553804	CG	Ler	LL	Region 2	B-E03	LerCol3F2
0.430300784	CHG	Ler	LL	Region 2	B-E03	LerCol3F2
0.060791709	CHH	Ler	LL	Region 2	B-E03	LerCol3F2
0.52579698	CG	Col	CL	Region 1	B-F03	LerCol3F2
0.162079763	CHG	Col	CL	Region 1	B-F03	LerCol3F2
0.02961821	CHH	Col	CL	Region 1	B-F03	LerCol3F2
0.086191537	CG	Ler	CL	Region 1	B-F03	LerCol3F2
0.012798036	CHG	Ler	CL	Region 1	B-F03	LerCol3F2
0.00840678	CHH	Ler	CL	Region 1	B-F03	LerCol3F2
0.937683364	CG	Ler	LL	Region 2	B-F03	LerCol3F2
0.535185297	CHG	Ler	LL	Region 2	B-F03	LerCol3F2
0.076637457	CHH	Ler	LL	Region 2	B-F03	LerCol3F2
0.838186482	CG	Col	CC	Region 1	B-G03	LerCol3F2
0.515021715	CHG	Col	CC	Region 1	B-G03	LerCol3F2
0.331172466	CHH	Col	CC	Region 1	B-G03	LerCol3F2
0.926158735	CG	Col	CL	Region 2	B-G03	LerCol3F2
0.517872611	CHG	Col	CL	Region 2	B-G03	LerCol3F2
0.072136246	CHH	Col	CL	Region 2	B-G03	LerCol3F2
0.917170423	CG	Ler	CL	Region 2	B-G03	LerCol3F2
0.485014718	CHG	Ler	CL	Region 2	B-G03	LerCol3F2
0.06982404	CHH	Ler	CL	Region 2	B-G03	LerCol3F2
0.514937836	CG	Col	CL	Region 1	B-H03	LerCol3F2
0.107897421	CHG	Col	CL	Region 1	B-H03	LerCol3F2
0.021269076	CHH	Col	CL	Region 1	B-H03	LerCol3F2
0.472162412	CG	Ler	CL	Region 1	B-H03	LerCol3F2
0.199321048	CHG	Ler	CL	Region 1	B-H03	LerCol3F2
0.022518431	CHH	Ler	CL	Region 1	B-H03	LerCol3F2
0.927885741	CG	Col	CL	Region 2	B-H03	LerCol3F2

0.507056452	CHG	Col	CL	Region 2	B-H03	LerCol3F2
0.068929769	CHH	Col	CL	Region 2	B-H03	LerCol3F2
0.927611093	CG	Ler	CL	Region 2	B-H03	LerCol3F2
0.482444336	CHG	Ler	CL	Region 2	B-H03	LerCol3F2
0.05907619	CHH	Ler	CL	Region 2	B-H03	LerCol3F2
0.894488189	CG	Col	CC	Region 1	B-A04	LerCol3F2
0.562329886	CHG	Col	CC	Region 1	B-A04	LerCol3F2
0.397434153	CHH	Col	CC	Region 1	B-A04	LerCol3F2
0.927414934	CG	Ler	LL	Region 2	B-A04	LerCol3F2
0.479146903	CHG	Ler	LL	Region 2	B-A04	LerCol3F2
0.063113631	CHH	Ler	LL	Region 2	B-A04	LerCol3F2
0.125433218	CG	Ler	LL	Region 1	B-B04	LerCol3F2
0.079327555	CHG	Ler	LL	Region 1	B-B04	LerCol3F2
0.130287394	CHH	Ler	LL	Region 1	B-B04	LerCol3F2
0.917454989	CG	Ler	LL	Region 2	B-B04	LerCol3F2
0.436736641	CHG	Ler	LL	Region 2	B-B04	LerCol3F2
0.057108437	CHH	Ler	LL	Region 2	B-B04	LerCol3F2
0.37039907	CG	Col	CL	Region 1	B-C04	LerCol3F2
0.039093398	CHG	Col	CL	Region 1	B-C04	LerCol3F2
0.013400054	CHH	Col	CL	Region 1	B-C04	LerCol3F2
0.017319964	CG	Ler	CL	Region 1	B-C04	LerCol3F2
0.008616047	CHG	Ler	CL	Region 1	B-C04	LerCol3F2
0.00843197	CHH	Ler	CL	Region 1	B-C04	LerCol3F2
0.91399195	CG	Col	CL	Region 2	B-C04	LerCol3F2
0.461806924	CHG	Col	CL	Region 2	B-C04	LerCol3F2
0.062778798	CHH	Col	CL	Region 2	B-C04	LerCol3F2
0.910051753	CG	Ler	CL	Region 2	B-C04	LerCol3F2
0.434502462	CHG	Ler	CL	Region 2	B-C04	LerCol3F2
0.05987123	CHH	Ler	CL	Region 2	B-C04	LerCol3F2
0.814792398	CG	Col	CC	Region 1	B-D04	LerCol3F2
0.454380173	CHG	Col	CC	Region 1	B-D04	LerCol3F2
0.255367326	CHH	Col	CC	Region 1	B-D04	LerCol3F2
0.935546436	CG	Col	CC	Region 2	B-D04	LerCol3F2
0.512988118	CHG	Col	CC	Region 2	B-D04	LerCol3F2
0.065631926	CHH	Col	CC	Region 2	B-D04	LerCol3F2
0.473982147	CG	Col	CL	Region 1	B-E04	LerCol3F2

0.114124872	CHG	Col	CL	Region 1	B-E04	LerCol3F2
0.024954398	CHH	Col	CL	Region 1	B-E04	LerCol3F2
0.080133051	CG	Ler	CL	Region 1	B-E04	LerCol3F2
0.020415473	CHG	Ler	CL	Region 1	B-E04	LerCol3F2
0.010980599	CHH	Ler	CL	Region 1	B-E04	LerCol3F2
0.912513527	CG	Ler	LL	Region 2	B-E04	LerCol3F2
0.472683483	CHG	Ler	LL	Region 2	B-E04	LerCol3F2
0.065744105	CHH	Ler	LL	Region 2	B-E04	LerCol3F2
0.825653031	CG	Col	CC	Region 1	B-F04	LerCol3F2
0.518339909	CHG	Col	CC	Region 1	B-F04	LerCol3F2
0.291990133	CHH	Col	CC	Region 1	B-F04	LerCol3F2
0.913636002	CG	Col	CC	Region 2	B-F04	LerCol3F2
0.518077484	CHG	Col	CC	Region 2	B-F04	LerCol3F2
0.065806078	CHH	Col	CC	Region 2	B-F04	LerCol3F2
0.053337922	CG	Ler	LL	Region 1	B-G04	LerCol4F2
0.015591253	CHG	Ler	LL	Region 1	B-G04	LerCol4F2
0.008568533	CHH	Ler	LL	Region 1	B-G04	LerCol4F2
0.919975004	CG	Col	CL	Region 2	B-G04	LerCol4F2
0.443429762	CHG	Col	CL	Region 2	B-G04	LerCol4F2
0.058268244	CHH	Col	CL	Region 2	B-G04	LerCol4F2
0.920556107	CG	Ler	CL	Region 2	B-G04	LerCol4F2
0.416319238	CHG	Ler	CL	Region 2	B-G04	LerCol4F2
0.048018311	CHH	Ler	CL	Region 2	B-G04	LerCol4F2
0.615755627	CG	Col	CC	Region 1	B-H04	LerCol4F2
0.233985049	CHG	Col	CC	Region 1	B-H04	LerCol4F2
0.143113379	CHH	Col	CC	Region 1	B-H04	LerCol4F2
0.914274706	CG	Col	CL	Region 2	B-H04	LerCol4F2
0.440035344	CHG	Col	CL	Region 2	B-H04	LerCol4F2
0.058699812	CHH	Col	CL	Region 2	B-H04	LerCol4F2
0.903428687	CG	Ler	CL	Region 2	B-H04	LerCol4F2
0.435142905	CHG	Ler	CL	Region 2	B-H04	LerCol4F2
0.061535861	CHH	Ler	CL	Region 2	B-H04	LerCol4F2
0.583237921	CG	Col	CL	Region 1	B-A05	LerCol4F2
0.190955677	CHG	Col	CL	Region 1	B-A05	LerCol4F2
0.024783417	CHH	Col	CL	Region 1	B-A05	LerCol4F2
0.169889914	CG	Ler	CL	Region 1	B-A05	LerCol4F2

0.056892523	CHG	Ler	CL	Region 1	B-A05	LerCol4F2
0.013305485	CHH	Ler	CL	Region 1	B-A05	LerCol4F2
0.927306684	CG	Col	CC	Region 2	B-A05	LerCol4F2
0.549891866	CHG	Col	CC	Region 2	B-A05	LerCol4F2
0.064769071	CHH	Col	CC	Region 2	B-A05	LerCol4F2
0.76787117	CG	Col	CL	Region 1	B-B05	LerCol4F2
0.353869969	CHG	Col	CL	Region 1	B-B05	LerCol4F2
0.185464425	CHH	Col	CL	Region 1	B-B05	LerCol4F2
0.368635002	CG	Ler	CL	Region 1	B-B05	LerCol4F2
0.213442402	CHG	Ler	CL	Region 1	B-B05	LerCol4F2
0.192182229	CHH	Ler	CL	Region 1	B-B05	LerCol4F2
0.927710064	CG	Col	CC	Region 2	B-B05	LerCol4F2
0.500148444	CHG	Col	CC	Region 2	B-B05	LerCol4F2
0.068835388	CHH	Col	CC	Region 2	B-B05	LerCol4F2
0.095992083	CG	Ler	LL	Region 1	B-C05	LerCol4F2
0.049474094	CHG	Ler	LL	Region 1	B-C05	LerCol4F2
0.058075237	CHH	Ler	LL	Region 1	B-C05	LerCol4F2
0.917935269	CG	Col	CL	Region 2	B-C05	LerCol4F2
0.537635557	CHG	Col	CL	Region 2	B-C05	LerCol4F2
0.06640105	CHH	Col	CL	Region 2	B-C05	LerCol4F2
0.873445305	CG	Ler	CL	Region 2	B-C05	LerCol4F2
0.487947079	CHG	Ler	CL	Region 2	B-C05	LerCol4F2
0.054117959	CHH	Ler	CL	Region 2	B-C05	LerCol4F2
0.136768559	CG	Ler	LL	Region 1	B-D05	LerCol4F2
0.065664883	CHG	Ler	LL	Region 1	B-D05	LerCol4F2
0.026162087	CHH	Ler	LL	Region 1	B-D05	LerCol4F2
0.916134268	CG	Ler	LL	Region 2	B-D05	LerCol4F2
0.515124621	CHG	Ler	LL	Region 2	B-D05	LerCol4F2
0.065000246	CHH	Ler	LL	Region 2	B-D05	LerCol4F2
0.851344918	CG	Col	CC	Region 1	B-E05	LerCol4F2
0.511636654	CHG	Col	CC	Region 1	B-E05	LerCol4F2
0.354599788	CHH	Col	CC	Region 1	B-E05	LerCol4F2
0.931635959	CG	Col	CL	Region 2	B-E05	LerCol4F2
0.503939162	CHG	Col	CL	Region 2	B-E05	LerCol4F2
0.06789927	CHH	Col	CL	Region 2	B-E05	LerCol4F2
0.925033921	CG	Ler	CL	Region 2	B-E05	LerCol4F2

0.478204257	CHG	Ler	CL	Region 2	B-E05	LerCol4F2
0.064342722	CHH	Ler	CL	Region 2	B-E05	LerCol4F2
0.803694033	CG	Col	CC	Region 1	B-F05	LerCol4F2
0.43839738	CHG	Col	CC	Region 1	B-F05	LerCol4F2
0.280238721	CHH	Col	CC	Region 1	B-F05	LerCol4F2
0.918776371	CG	Ler	LL	Region 2	B-F05	LerCol4F2
0.465310337	CHG	Ler	LL	Region 2	B-F05	LerCol4F2
0.061515718	CHH	Ler	LL	Region 2	B-F05	LerCol4F2
0.695539906	CG	Col	CL	Region 1	B-G05	LerCol4F2
0.30553758	CHG	Col	CL	Region 1	B-G05	LerCol4F2
0.070919683	CHH	Col	CL	Region 1	B-G05	LerCol4F2
0.314643603	CG	Ler	CL	Region 1	B-G05	LerCol4F2
0.203077284	CHG	Ler	CL	Region 1	B-G05	LerCol4F2
0.064570437	CHH	Ler	CL	Region 1	B-G05	LerCol4F2
0.927999157	CG	Col	CL	Region 2	B-G05	LerCol4F2
0.562177382	CHG	Col	CL	Region 2	B-G05	LerCol4F2
0.072299317	CHH	Col	CL	Region 2	B-G05	LerCol4F2
0.9219022	CG	Ler	CL	Region 2	B-G05	LerCol4F2
0.548271988	CHG	Ler	CL	Region 2	B-G05	LerCol4F2
0.067152443	CHH	Ler	CL	Region 2	B-G05	LerCol4F2
0.016123883	CG	Ler	LL	Region 1	B-A06	LerCol4F2
0.008879652	CHG	Ler	LL	Region 1	B-A06	LerCol4F2
0.007334317	CHH	Ler	LL	Region 1	B-A06	LerCol4F2
0.914451215	CG	Ler	LL	Region 2	B-A06	LerCol4F2
0.458199492	CHG	Ler	LL	Region 2	B-A06	LerCol4F2
0.051915902	CHH	Ler	LL	Region 2	B-A06	LerCol4F2
0.701829506	CG	Col	CL	Region 1	B-B06	LerCol4F2
0.298096115	CHG	Col	CL	Region 1	B-B06	LerCol4F2
0.170496727	CHH	Col	CL	Region 1	B-B06	LerCol4F2
0.244546248	CG	Ler	CL	Region 1	B-B06	LerCol4F2
0.145751073	CHG	Ler	CL	Region 1	B-B06	LerCol4F2
0.127812305	CHH	Ler	CL	Region 1	B-B06	LerCol4F2
0.922444321	CG	Ler	LL	Region 2	B-B06	LerCol4F2
0.477771871	CHG	Ler	LL	Region 2	B-B06	LerCol4F2
0.066780317	CHH	Ler	LL	Region 2	B-B06	LerCol4F2
0.778228533	CG	Col	CC	Region 1	B-C06	LerCol4F2

0.369200244	CHG	Col	CC	Region 1	B-C06	LerCol4F2
0.191622275	CHH	Col	CC	Region 1	B-C06	LerCol4F2
0.934516732	CG	Col	CL	Region 2	B-C06	LerCol4F2
0.46922496	CHG	Col	CL	Region 2	B-C06	LerCol4F2
0.055164652	CHH	Col	CL	Region 2	B-C06	LerCol4F2
0.931058795	CG	Ler	CL	Region 2	B-C06	LerCol4F2
0.449915717	CHG	Ler	CL	Region 2	B-C06	LerCol4F2
0.051325474	CHH	Ler	CL	Region 2	B-C06	LerCol4F2
0.529920692	CG	Col	CL	Region 1	B-D06	LerCol4F2
0.129402901	CHG	Col	CL	Region 1	B-D06	LerCol4F2
0.028269359	CHH	Col	CL	Region 1	B-D06	LerCol4F2
0.288941335	CG	Ler	CL	Region 1	B-D06	LerCol4F2
0.097270077	CHG	Ler	CL	Region 1	B-D06	LerCol4F2
0.020022182	CHH	Ler	CL	Region 1	B-D06	LerCol4F2
0.939685511	CG	Col	CL	Region 2	B-D06	LerCol4F2
0.500140656	CHG	Col	CL	Region 2	B-D06	LerCol4F2
0.057536209	CHH	Col	CL	Region 2	B-D06	LerCol4F2
0.848178459	CG	Ler	CL	Region 2	B-D06	LerCol4F2
0.418066183	CHG	Ler	CL	Region 2	B-D06	LerCol4F2
0.042470343	CHH	Ler	CL	Region 2	B-D06	LerCol4F2
0.483207676	CG	Col	CL	Region 1	B-E06	LerCol4F2
0.119000539	CHG	Col	CL	Region 1	B-E06	LerCol4F2
0.021311183	CHH	Col	CL	Region 1	B-E06	LerCol4F2
0.151534527	CG	Ler	CL	Region 1	B-E06	LerCol4F2
0.035187287	CHG	Ler	CL	Region 1	B-E06	LerCol4F2
0.009435013	CHH	Ler	CL	Region 1	B-E06	LerCol4F2
0.923506439	CG	Col	CL	Region 2	B-E06	LerCol4F2
0.51800345	CHG	Col	CL	Region 2	B-E06	LerCol4F2
0.062466285	CHH	Col	CL	Region 2	B-E06	LerCol4F2
0.915790232	CG	Ler	CL	Region 2	B-E06	LerCol4F2
0.485165794	CHG	Ler	CL	Region 2	B-E06	LerCol4F2
0.056759478	CHH	Ler	CL	Region 2	B-E06	LerCol4F2
0.80509165	CG	Col	CC	Region 1	B-F06	LerCol4F2
0.454706071	CHG	Col	CC	Region 1	B-F06	LerCol4F2
0.203946037	CHH	Col	CC	Region 1	B-F06	LerCol4F2
0.916848371	CG	Col	CL	Region 2	B-F06	LerCol4F2

0.516771592	CHG	Col	CL	Region 2	B-F06	LerCol4F2
0.060454302	CHH	Col	CL	Region 2	B-F06	LerCol4F2
0.911845819	CG	Ler	CL	Region 2	B-F06	LerCol4F2
0.509088942	CHG	Ler	CL	Region 2	B-F06	LerCol4F2
0.06043123	CHH	Ler	CL	Region 2	B-F06	LerCol4F2
0.698200993	CG	Col	CL	Region 1	B-G06	LerCol4F2
0.258384333	CHG	Col	CL	Region 1	B-G06	LerCol4F2
0.055038948	CHH	Col	CL	Region 1	B-G06	LerCol4F2
0.459383754	CG	Ler	CL	Region 1	B-G06	LerCol4F2
0.259537784	CHG	Ler	CL	Region 1	B-G06	LerCol4F2
0.052488871	CHH	Ler	CL	Region 1	B-G06	LerCol4F2
0.913671266	CG	Col	CC	Region 2	B-G06	LerCol4F2
0.523061962	CHG	Col	CC	Region 2	B-G06	LerCol4F2
0.06597079	CHH	Col	CC	Region 2	B-G06	LerCol4F2
0.166507406	CG	Ler	LL	Region 1	B-H06	LerCol4F2
0.095414479	CHG	Ler	LL	Region 1	B-H06	LerCol4F2
0.055122178	CHH	Ler	LL	Region 1	B-H06	LerCol4F2
0.941871818	CG	Col	CL	Region 2	B-H06	LerCol4F2
0.535317067	CHG	Col	CL	Region 2	B-H06	LerCol4F2
0.057573673	CHH	Col	CL	Region 2	B-H06	LerCol4F2
0.938559242	CG	Ler	CL	Region 2	B-H06	LerCol4F2
0.515971133	CHG	Ler	CL	Region 2	B-H06	LerCol4F2
0.053422313	CHH	Ler	CL	Region 2	B-H06	LerCol4F2
0.671162171	CG	Col	CL	Region 1	B-A07	LerCol4F2
0.22987302	CHG	Col	CL	Region 1	B-A07	LerCol4F2
0.109432333	CHH	Col	CL	Region 1	B-A07	LerCol4F2
0.359267735	CG	Ler	CL	Region 1	B-A07	LerCol4F2
0.187020605	CHG	Ler	CL	Region 1	B-A07	LerCol4F2
0.122646664	CHH	Ler	CL	Region 1	B-A07	LerCol4F2
0.910379412	CG	Col	CC	Region 2	B-A07	LerCol4F2
0.440797629	CHG	Col	CC	Region 2	B-A07	LerCol4F2
0.049994368	CHH	Col	CC	Region 2	B-A07	LerCol4F2
0.676624225	CG	Col	CL	Region 1	B-B07	LerCol4F2
0.295915085	CHG	Col	CL	Region 1	B-B07	LerCol4F2
0.143014862	CHH	Col	CL	Region 1	B-B07	LerCol4F2
0.623866015	CG	Ler	CL	Region 1	B-B07	LerCol4F2

0.354708684	CHG	Ler	CL	Region 1	B-B07	LerCol4F2
0.16879058	CHH	Ler	CL	Region 1	B-B07	LerCol4F2
0.931985172	CG	Col	CL	Region 2	B-B07	LerCol4F2
0.466957916	CHG	Col	CL	Region 2	B-B07	LerCol4F2
0.063765549	CHH	Col	CL	Region 2	B-B07	LerCol4F2
0.927541456	CG	Ler	CL	Region 2	B-B07	LerCol4F2
0.447148106	CHG	Ler	CL	Region 2	B-B07	LerCol4F2
0.059392598	CHH	Ler	CL	Region 2	B-B07	LerCol4F2
0.652958456	CG	Col	CL	Region 1	B-C07	LerCol4F2
0.190472259	CHG	Col	CL	Region 1	B-C07	LerCol4F2
0.057214518	CHH	Col	CL	Region 1	B-C07	LerCol4F2
0.21277205	CG	Ler	CL	Region 1	B-C07	LerCol4F2
0.093495014	CHG	Ler	CL	Region 1	B-C07	LerCol4F2
0.044458328	CHH	Ler	CL	Region 1	B-C07	LerCol4F2
0.861897024	CG	Ler	LL	Region 2	B-C07	LerCol4F2
0.367560514	CHG	Ler	LL	Region 2	B-C07	LerCol4F2
0.039534388	CHH	Ler	LL	Region 2	B-C07	LerCol4F2
0.012060908	CG	Ler	LL	Region 1	B-D07	LerCol4F2
0.007600499	CHG	Ler	LL	Region 1	B-D07	LerCol4F2
0.00703893	CHH	Ler	LL	Region 1	B-D07	LerCol4F2
0.849671298	CG	Ler	LL	Region 2	B-D07	LerCol4F2
0.40045457	CHG	Ler	LL	Region 2	B-D07	LerCol4F2
0.045230117	CHH	Ler	LL	Region 2	B-D07	LerCol4F2
0.138209983	CG	Ler	LL	Region 1	B-E07	LerCol4F2
0.045439109	CHG	Ler	LL	Region 1	B-E07	LerCol4F2
0.010845726	CHH	Ler	LL	Region 1	B-E07	LerCol4F2
0.851843489	CG	Col	CL	Region 2	B-E07	LerCol4F2
0.481565575	CHG	Col	CL	Region 2	B-E07	LerCol4F2
0.048185324	CHH	Col	CL	Region 2	B-E07	LerCol4F2
0.920586141	CG	Ler	CL	Region 2	B-E07	LerCol4F2
0.494615577	CHG	Ler	CL	Region 2	B-E07	LerCol4F2
0.051181327	CHH	Ler	CL	Region 2	B-E07	LerCol4F2
0.486081782	CG	Col	CL	Region 1	B-F07	LerCol4F2
0.094348188	CHG	Col	CL	Region 1	B-F07	LerCol4F2
0.017741021	CHH	Col	CL	Region 1	B-F07	LerCol4F2
0.035674779	CG	Ler	CL	Region 1	B-F07	LerCol4F2

0.014557306	CHG	Ler	CL	Region 1	B-F07	LerCol4F2
0.007986378	CHH	Ler	CL	Region 1	B-F07	LerCol4F2
0.926577614	CG	Col	CL	Region 2	B-F07	LerCol4F2
0.476749551	CHG	Col	CL	Region 2	B-F07	LerCol4F2
0.054652495	CHH	Col	CL	Region 2	B-F07	LerCol4F2
0.924253966	CG	Ler	CL	Region 2	B-F07	LerCol4F2
0.456284908	CHG	Ler	CL	Region 2	B-F07	LerCol4F2
0.052104712	CHH	Ler	CL	Region 2	B-F07	LerCol4F2
0.788644054	CG	Col	CC	Region 1	B-G07	LerCol4F2
0.377540386	CHG	Col	CC	Region 1	B-G07	LerCol4F2
0.201223517	CHH	Col	CC	Region 1	B-G07	LerCol4F2
0.916321204	CG	Ler	LL	Region 2	B-G07	LerCol4F2
0.418059023	CHG	Ler	LL	Region 2	B-G07	LerCol4F2
0.04939215	CHH	Ler	LL	Region 2	B-G07	LerCol4F2
0.528825055	CG	Col	CL	Region 1	B-H07	LerCol4F2
0.152695789	CHG	Col	CL	Region 1	B-H07	LerCol4F2
0.059081228	CHH	Col	CL	Region 1	B-H07	LerCol4F2
0.070851165	CG	Ler	CL	Region 1	B-H07	LerCol4F2
0.036978094	CHG	Ler	CL	Region 1	B-H07	LerCol4F2
0.034546959	CHH	Ler	CL	Region 1	B-H07	LerCol4F2
0.85495306	CG	Ler	LL	Region 2	B-H07	LerCol4F2
0.410728536	CHG	Ler	LL	Region 2	B-H07	LerCol4F2
0.049355243	CHH	Ler	LL	Region 2	B-H07	LerCol4F2
0.759294995	CG	Col	CC	Region 1	B-A08	LerCol4F2
0.30427498	CHG	Col	CC	Region 1	B-A08	LerCol4F2
0.088768156	CHH	Col	CC	Region 1	B-A08	LerCol4F2
0.924934918	CG	Col	CC	Region 2	B-A08	LerCol4F2
0.454998678	CHG	Col	CC	Region 2	B-A08	LerCol4F2
0.046877362	CHH	Col	CC	Region 2	B-A08	LerCol4F2
0.052015088	CG	Ler	LL	Region 1	B-B08	LerCol4F2
0.011328125	CHG	Ler	LL	Region 1	B-B08	LerCol4F2
0.008355488	CHH	Ler	LL	Region 1	B-B08	LerCol4F2
0.936983842	CG	Col	CC	Region 2	B-B08	LerCol4F2
0.539608414	CHG	Col	CC	Region 2	B-B08	LerCol4F2
0.053322945	CHH	Col	CC	Region 2	B-B08	LerCol4F2
0.719740049	CG	Col	CL	Region 1	B-C08	LerCol4F2

0.347257926	CHG	Col	CL	Region 1	B-C08	LerCol4F2
0.136816679	CHH	Col	CL	Region 1	B-C08	LerCol4F2
0.245481433	CG	Ler	CL	Region 1	B-C08	LerCol4F2
0.176159794	CHG	Ler	CL	Region 1	B-C08	LerCol4F2
0.128537769	CHH	Ler	CL	Region 1	B-C08	LerCol4F2
0.910597341	CG	Ler	LL	Region 2	B-C08	LerCol4F2
0.53498506	CHG	Ler	LL	Region 2	B-C08	LerCol4F2
0.06840445	CHH	Ler	LL	Region 2	B-C08	LerCol4F2
0.743702081	CG	Col	CL	Region 1	B-D08	LerCol4F2
0.329011812	CHG	Col	CL	Region 1	B-D08	LerCol4F2
0.169009635	CHH	Col	CL	Region 1	B-D08	LerCol4F2
0.255010661	CG	Ler	CL	Region 1	B-D08	LerCol4F2
0.152596315	CHG	Ler	CL	Region 1	B-D08	LerCol4F2
0.143230605	CHH	Ler	CL	Region 1	B-D08	LerCol4F2
0.93491767	CG	Col	CL	Region 2	B-D08	LerCol4F2
0.514256693	CHG	Col	CL	Region 2	B-D08	LerCol4F2
0.068798143	CHH	Col	CL	Region 2	B-D08	LerCol4F2
0.928567866	CG	Ler	CL	Region 2	B-D08	LerCol4F2
0.488030695	CHG	Ler	CL	Region 2	B-D08	LerCol4F2
0.063251414	CHH	Ler	CL	Region 2	B-D08	LerCol4F2

Supplementary Table S6   BLASTN search of region 1 & 2 in nuclear genome

Query	Subject	%homology	Length	query start	query end	subject start	subject end	evalue	score
Region 1	NC_003074.8	100	194	1	194	16574327	16574520	3.94E-96	351
(Chr3: 16574327..16574520)	NC_003074.8	85.714	28	88	115	10957907	10957880	1.4	33.7
	NC_003074.8	95	20	148	167	1310505	1310524	4.8	31.9
	NC_003074.8	88	25	92	116	8498072	8498096	4.8	31.9
	NC_003074.8	82.857	35	95	127	11797694	11797726	4.8	31.9
	NC_003074.8	95	20	173	192	19588363	19588382	4.8	31.9
	NC_003076.8	83.784	37	131	167	4530896	4530931	0.11	37.4
	NC_003076.8	100	18	91	108	5269926	5269909	1.4	33.7
	NC_003076.8	91.304	23	8	30	8442048	8442070	1.4	33.7
	NC_003076.8	86.667	30	122	150	14250474	14250503	1.4	33.7
	NC_003076.8	91.304	23	92	114	16930225	16930247	1.4	33.7
	NC_003076.8	91.304	23	130	152	17847755	17847777	1.4	33.7
	NC_003076.8	88.462	26	8	33	25050969	25050944	1.4	33.7
	NC_003076.8	88.889	27	159	184	875451	875425	4.8	31.9
	NC_003076.8	100	17	172	188	6321088	6321072	4.8	31.9
	NC_003076.8	95	20	86	105	9788535	9788516	4.8	31.9
	NC_003076.8	85.185	27	145	171	12630759	12630733	4.8	31.9
	NC_003076.8	88	25	91	115	21412086	21412062	4.8	31.9
	NC_003076.8	88	25	82	106	24816273	24816297	4.8	31.9
	NC_003070.9	89.286	28	68	95	21197751	21197724	0.11	37.4
	NC_003070.9	91.667	24	21	44	539007	539030	0.4	35.6

NC_003070.9	91.667	24	69	92	784731	784708	0.4	35.6
NC_003070.9	89.286	28	91	116	18238124	18238151	0.4	35.6
NC_003070.9	79.545	44	146	188	23294086	23294129	0.4	35.6
NC_003070.9	90.909	22	85	106	2316432	2316411	4.8	31.9
NC_003070.9	95	20	84	103	2993857	2993838	4.8	31.9
NC_003070.9	95	20	14	33	7277405	7277386	4.8	31.9
NC_003070.9	88	25	143	167	7682737	7682761	4.8	31.9
NC_003070.9	80.556	36	150	185	11411221	11411253	4.8	31.9
NC_003070.9	80	35	137	171	14457508	14457542	4.8	31.9
NC_003070.9	100	17	177	193	19683805	19683789	4.8	31.9
NC_003070.9	95	20	15	34	19965363	19965382	4.8	31.9
NC_003070.9	100	17	25	41	26468731	26468715	4.8	31.9
NC_003071.7	91.667	24	85	108	8267106	8267129	0.4	35.6
NC_003071.7	81.081	37	131	167	18286713	18286679	1.4	33.7
NC_003071.7	90.909	22	86	107	899921	899942	4.8	31.9
NC_003071.7	78.571	42	91	132	8458064	8458104	4.8	31.9
NC_003071.7	100	17	178	194	19052605	19052589	4.8	31.9
NC_003075.7	95.238	21	146	166	7797147	7797167	1.4	33.7
NC_003075.7	88	25	160	184	3632363	3632339	4.8	31.9
NC_003075.7	95	20	75	94	8123542	8123523	4.8	31.9
NC_003075.7	100	17	135	151	10167761	10167745	4.8	31.9
NC_003075.7	85.714	28	161	188	10489418	10489442	4.8	31.9

Region 2	NC_003075.7	100	260	1	260	6341136	6341395	8.15E-132	470
(Chr4: 6341136..6341395)	NC_003075.7	81.818	33	69	101	12071441	12071473	1.9	33.7
	NC_003075.7	85.714	28	68	95	15808007	15808034	1.9	33.7
	NC_003075.7	95	20	125	144	62004	62023	6.7	31.9
	NC_003075.7	84.375	32	63	93	4167889	4167920	6.7	31.9
	NC_003075.7	95	20	73	92	5598753	5598734	6.7	31.9
	NC_003075.7	90.909	22	79	100	6640708	6640687	6.7	31.9
	NC_003075.7	85.714	28	59	86	7855492	7855516	6.7	31.9
	NC_003075.7	90.909	22	83	104	13691361	13691340	6.7	31.9
	NC_003071.7	90.26	154	1	153	3294203	3294050	1.48E-52	206
	NC_003071.7	95.833	48	151	198	3416715	3416762	5.17E-14	78.8
	NC_003071.7	96.296	27	234	260	3259261	3259287	0.001	44.6
	NC_003071.7	87.097	31	66	96	13656321	13656351	0.045	39.2
	NC_003071.7	91.667	24	77	100	11628536	11628559	0.55	35.6
	NC_003071.7	85.714	28	70	97	8345227	8345200	1.9	33.7
	NC_003071.7	83.333	36	58	91	11647753	11647786	1.9	33.7
	NC_003071.7	100	17	70	86	1589727	1589743	6.7	31.9
	NC_003071.7	81.25	32	53	84	4415403	4415372	6.7	31.9
	NC_003071.7	90.909	22	80	101	4476727	4476748	6.7	31.9
	NC_003071.7	90.909	22	131	152	8820713	8820692	6.7	31.9
	NC_003071.7	90.909	22	70	91	17907668	17907689	6.7	31.9
	NC_003074.8	87.5	32	70	100	16292665	16292696	0.16	37.4
	NC_003074.8	91.667	24	77	100	5417544	5417521	0.55	35.6

NC_003074.8	79.487	39	124	162	10583081	10583119	0.55	35.6
NC_003074.8	89.286	28	124	151	11120973	11120948	0.55	35.6
NC_003074.8	85.714	28	192	219	18117042	18117015	1.9	33.7
NC_003074.8	88.462	26	131	156	18831080	18831105	1.9	33.7
NC_003074.8	90.909	22	18	39	3060486	3060465	6.7	31.9
NC_003074.8	86.207	29	67	94	4681053	4681081	6.7	31.9
NC_003074.8	90.909	22	78	99	8545415	8545436	6.7	31.9
NC_003074.8	88	25	67	91	9058074	9058050	6.7	31.9
NC_003074.8	95.455	22	67	88	16208070	16208050	6.7	31.9
NC_003074.8	100	17	56	72	18821135	18821151	6.7	31.9
NC_003074.8	95	20	205	224	19085785	19085804	6.7	31.9
NC_003076.8	100	19	86	104	7958151	7958133	0.55	35.6
NC_003076.8	95.455	22	73	94	16409766	16409745	0.55	35.6
NC_003076.8	95.455	22	122	143	18146787	18146766	0.55	35.6
NC_003076.8	86.207	29	161	189	21758572	21758544	0.55	35.6
NC_003076.8	100	18	85	102	18337399	18337382	1.9	33.7
NC_003076.8	88.462	26	131	156	21814724	21814749	1.9	33.7
NC_003076.8	81.25	32	6	37	2341249	2341280	6.7	31.9
NC_003076.8	90.909	22	80	101	12569493	12569514	6.7	31.9
NC_003076.8	88.462	26	75	100	14903690	14903667	6.7	31.9
NC_003076.8	90.909	22	62	83	18172201	18172180	6.7	31.9
NC_003076.8	95	20	81	100	20821360	20821341	6.7	31.9
NC_003076.8	81.579	38	67	101	22537802	22537765	6.7	31.9

NC_003070.9	91.304	23	79	101	11063045	11063023	1.9	33.7
NC_003070.9	91.304	23	79	101	24013078	24013056	1.9	33.7
NC_003070.9	95.652	23	175	197	24439859	24439880	1.9	33.7
NC_003070.9	88.462	26	131	156	24918439	24918414	1.9	33.7
NC_003070.9	88.462	26	115	140	28953545	28953570	1.9	33.7
NC_003070.9	90.909	22	79	100	3318818	3318839	6.7	31.9
NC_003070.9	83.333	30	116	145	17443217	17443246	6.7	31.9
NC_003070.9	90.909	22	74	95	17648101	17648080	6.7	31.9
NC_003070.9	86.207	29	68	95	20446559	20446531	6.7	31.9
NC_003070.9	95	20	84	103	22324791	22324772	6.7	31.9
NC_003070.9	90.909	22	67	88	22501897	22501876	6.7	31.9
NC_003070.9	100	17	66	82	23714121	23714105	6.7	31.9
NC_003070.9	100	17	71	87	25261407	25261423	6.7	31.9
NC_003070.9	88.889	27	66	92	28123874	28123899	6.7	31.9
